# Sevoflurane Aggravates the Progress of Alzheimer’s Disease Through NLRP3/Caspase-1/Gasdermin D Pathway

**DOI:** 10.3389/fcell.2021.801422

**Published:** 2022-01-19

**Authors:** Di Tian, Yanmei Xing, Wenli Gao, Hongyan Zhang, Yifeng Song, Ya Tian, Zhongliang Dai

**Affiliations:** ^1^ Department of Anesthesiology, Shenzhen People's Hospital (The Second Clinical Medical College, Jinan University), Shenzhen, China; ^2^ Department of Anesthesiology, The First Affiliated Hospital, Southern University of Science and Technology, Shenzhen, China; ^3^ Shenzhen Engineering Research Center of Anesthesiology, Shenzhen, China

**Keywords:** gasdermin D, pyroptosis, sevoflurane, tau pathology, VX-765

## Abstract

**Background:** Alzheimer’s disease (AD) is the most common form of dementia worldwide. Previous studies have reported that sevoflurane, a frequently used anesthetic, can induce cognitive impairment in preclinical and clinical settings. However, the mechanism underlying the development of this neurotoxicity is currently unclear.

**Methods:** Seven-month-old APP/PS1 mice were placed in an anesthesia induction box containing 3% sevoflurane in 100% O_2_ for 6 h, while BV2 cells were cultured with 4% sevoflurane for 6 h. Pyroptosis and tau protein expression in excised hippocampus tissues and cells were measured using Western blotting and immunofluorescence assay. Caspase-1 and NLRP3 were knocked out in BV2 microglia using CRISPR/Cas9 technology to determine whether they mediate the effects induced by sevoflurane.

**Results:** Sevoflurane directly activated caspase-1 to induce pyroptosis in the mouse model of AD via NLRP3 and AIM2 activation. In addition, sevoflurane mediated cleavage of gasdermin D (GSDMD) but not gasdermin E (GSDME), promoted the biosynthesis of downstream interleukin-1*β* and interleukin-18, and increased *β*-amyloid (A*β*) deposition and tau phosphorylation. The nontoxic caspase-1 small-molecule inhibitor VX-765 significantly inhibited this activation process in microglia, while NLRP3 deletion suppressed sevoflurane-induced caspase-1 cleavage and subsequently pyroptosis, as well as tau pathology. Furthermore, silencing caspase-1 alleviated the sevoflurane-induced release of IL-1β and IL-18 and inhibited tau-related enzymes in microglia.

**Conclusion:** This study is the first to report that clinical doses of sevoflurane aggravate the progression of AD via the NLRP3/caspase-1/GSDMD axis. Collectively, our findings elucidate the crucial mechanisms of NLRP3/caspase-1 in pyroptosis and tau pathogenesis induced by sevoflurane and suggest that VX-765 could represent a novel therapeutic intervention for treating AD.

## Introduction

Alzheimer’s disease (AD) is a degenerative brain disorder that involves the deterioration of neurons in the entorhinal cortex, basal forebrain, cortex, and hippocampus (Douchamps and Mathis, 2017; Adewale et al., 2021). The two main pathological hallmarks of AD include the deposition of amyloid-*β* (A*β*) in senile plaques and the formation of neurofibrillary tangles due to pathological changes in microtubule-associated proteins, such as tau (Maloney and Lahiri, 2011; Douchamps and Mathis, 2017; Flores et al., 2018). Unfortunately, there are currently no approved treatments that can significantly slow the early degenerative events during the development of AD or cure the associated cognitive impairment or pathologies.

Sevoflurane is a colorless and volatile liquid anesthetic with a peculiar smell that is used to maintain clinical general anesthesia (Lu et al., 2010; Jiang et al., 2017; Yukina et al., 2019). Although sevoflurane has numerous advantages, such as universal applicability and safety over other anesthetics, several studies have demonstrated that sevoflurane administration can cause cognitive impairment including postoperative cognitive dysfunction (POCD) and AD (Lu et al., 2010; Xu et al., 2016). Certain inhaled anesthetics have been reported to enhance the formation of Aβ plaques and neurofibrillary tangles in animal models (Xie et al., 2006; Ikeda et al., 2007; Planel et al., 2007; Bianchi et al., 2008; Zheng et al., 2013). In addition, sevoflurane anesthesia has been shown to not only lead to neurotoxicity in the brains of pregnant and neonatal mice (Lu et al., 2010; Jiang et al., 2017) but also promote neuropathogenesis by inducing apoptosis and causing Aβ deposition (Xie et al., 2006). However, the mechanisms underlying these effects remain controversial and largely unclear.

Mouse models of AD have provided increasing evidence of the role of inflammation in AD. For instance, Grimaldi *et al.* identified tau protein tangles, A*β* deposition, astrogliosis, and neurodegeneration in pre-symptomatic 3xTg-AD mice by using new retinal biomarkers (Alfonso et al., 2018; Adewale et al., 2021). The formation of A*β* plaques followed by sustained neuroinflammation was also found to be a crucial cause of cognitive disorder in an App-KI mouse model of AD (Maloney and Lahiri, 2011). The innate immune response is a complex physiological process that occurs around microglia, which plays a key role in the development of some of the main pathological features of AD (Li et al., 2017; Dong et al., 2021). Under normal conditions, intracerebral microglia maintain the balance between tau and Aβ deposition and clearance (Tang et al., 2011; Xue et al., 2020); and several studies have implicated microglial dysfunction in the pathogenesis of AD (He et al., 2015; Sung et al., 2019).

Pyroptosis is a form of programmed cell death that relies on caspase-1 activation, ultimately causes plasma membrane rupture, and promotes inflammatory reactions (Heneka et al., 2013). Previous studies have reported that the development of pyroptosis is closely related to the activation of inflammasomes (Shi et al., 2017), which are multiprotein complexes formed by Nod-like receptors (NLR) and apoptosis-associated speck-like proteins, including the caspase-1 precursor and a caspase recruitment domain (ASC) (Peng et al., 2021). Caspase-1 undergoes auto-activation following dimerization, allowing it to cleave pro-inflammatory cytokines. Gasdermin D (GSDMD) is a pyroptosis executioner protein that can be cleaved by caspase-1, leading to the formation of pores in the plasma membrane and subsequent cell lysis due to ion flux and cytoplasmic swelling (Sanders et al., 2015). During the pathogenesis of common neurodegenerative diseases, continuous stimulation by misfolded proteins (e.g., *a*-synuclein or A*β*) induces chronic NLRP3 inflammasome activation, leading to neuropathology (Broz, 2015); however, it remains unclear whether NLRP3-dependent pyroptosis plays a role in sevoflurane-induced neuropathy.

In this study, we aimed to investigate whether clinical doses of sevoflurane could induce AD and elucidate the underlying mechanism using *in vivo* and *in vitro* models of AD. Collectively, our findings suggest for the first time that sevoflurane induces pyroptosis via caspase-1 and GSDMD in APP/PS1 mice and microglia. Furthermore, we propose that VX-765 could potentially be a promising new therapeutic drug for treating AD.

## Materials and Methods

### Chemicals and Reagents

Sevoflurane was provided by the Department of Anesthesiology at Shenzhen People’s Hospital (Shenzhen, China). Lipopolysaccharide (LPS; 124S032) was purchased from Solarbio (Beijing, China). VX-765 (HY13205) was purchased from Sigma-Aldrich (Darmstadt, Germany). Anti-GSDMD (ab209845), anti-NLRP1 (ab98181), anti-NLRP3 (ab270449), anti-Tau (phosphor T231; ab151559), anti-Tau (ab32057), anti-GAPDH (ab8245), anti-IL-18 (ab207323), anti-IL-1β (ab234437), anti-pro caspase-1 + p10 + p12 (ab179515), and anti-caspase-3 (ab179517) antibodies were purchased from Abcam (Cambridge, UK). Anti-D-PP2A (#3281733) antibodies were purchased from EMD Millipore (MA, Burlington, United States). Anti-ASC/TMS1 (#67824), anti-PP2A (#2259), anti-GSK 3β (#12456), anti-p-GSK 3β (#9323), anti-CaMKII-α (#50049), anti-p-CaMKII-α (#12716), and anti-PP2A C subunit (52F8; #2259) antibodies were purchased from Cell Signaling Technology (Beverly, MA, United States). Goat anti-rabbit (A0208) and goat anti-mouse (A0216) antibodies were purchased from Beyotime (Shanghai, China).

### Animal Model and Sevoflurane Exposure

Seven-month-old APP/PS1 mice were obtained from Guangdong Medical Laboratory Animal Center (Guangzhou, China). Before the experiments, the mice were maintained under standard conditions (12/12 h light–dark cycle, 55% ± 5% humidity, 23°C ± 1°C) with free access to food and water. The mice were then randomly divided into a control group and a sevoflurane group (*n* = 9 per group). The control group received 100% O_2_ for 6 h, whereas the sevoflurane group received 3% sevoflurane in O_2_ for 6 h in the anesthesia induction box. The temperature of the box was controlled at 24°C–28°C using warming mats, as monitored by a thermometer. O_2_ and anesthetic concentrations were monitored continuously. After sevoflurane exposure, all animals were euthanized, and their hippocampi were dissected for Western blotting analysis.

### Cell Culture and Sevoflurane Administration

BV2 microglia were purchased from Procell Life Science & Technology (Wuhan, China) and cultured in Dulbecco’s modified Eagle’s medium (DMEM; Gibco, Grand Island, NY, United States), containing 10% fetal bovine serum (FBS) and 1% antibiotics (100 μg/ml of streptomycin and 100 U/ml of penicillin). The cells in the model group were pretreated with LPS (100 ng/ml) for 12 h and then cultured in dishes or plates at 37°C with 5% CO_2_. Next, the cells were incubated in a 37°C water bath for 6 h within a sealed acrylic box containing 21% oxygen, 5% CO_2_, and 4% sevoflurane delivered using a vaporizer (RWD R582S, Shenzhen, China). The outlet port was connected to a compact anesthesia monitor (Helsinki, Finland) that constantly monitored the O_2_, CO_2_, and sevoflurane concentrations.

### CRISPR/Cas9 Gene Knockout

Caspase-1 and NLRP3 genes were knocked out in BV2 cells using CRISPR/Cas9 technology. Two pairs of CRISPR–Cas9 sgRNAs were designed to target exon 1 of caspase-1 (NM_009,807.2) and exon 1 of NLRP3 (NM_001359638.1) using the crisp r.mit.edu website, as follows: Casp1-sgRNA: 5′-TCT CTA AAA AAG GGC CCC-3′; NLRP3-sgRNA: 5′-GAT ACT GAG CCA GCT TGC AA-3′. After the sgRNAs had annealed, they were inserted into plasmids (LentiCRISPR-V2-GFP; Qijing-Biology, Wuhan, China) to express sgRNA and Cas9. The recombinant plasmids and lentivirus helper plasmids were cotransfected into HEK 293T cells and incubated for 48–72 h. The supernatants were then collected, filtered using a 0.45-μm polyvinylidene fluoride (PVDF) membrane, and centrifuged at 50,000 × *g* and 4°C for 2 h. BV2 cells were transfected with the collected lentivirus, cultured at 37°C with 5% CO_2_ for 48 h, and observed using a microscope. Single-cell suspensions were sorted using a Sony flow cytometer (Tokyo, Japan, LE-SH800ZBP). Positive cells were separated and seeded into 96-well plates. After cell proliferation, protein expression was detected using Western blotting analysis, and genomic DNA was subjected to Sanger sequencing to confirm the sgRNA cut site.

### Lactate Dehydrogenase Release Assay

Cell death was detected using a CytoTox 96 Non-Radioactive Cytotoxicity Assay kit (Promega, Madison, WI, United States) according to the manufacturer’s protocol. Briefly, 100 μl of supernatant was removed from sevoflurane-treated BV2 cells and transferred into a clear flat-bottomed 96-well plate. A freshly prepared reaction mixture (100 μl) was then added and incubated for no longer than 30 min at 20–25°C. Lactate dehydrogenase (LDH) activity was detected using a Tecan microplate reader (Spark 10M, Männedorf, Switzerland).

### Cell Counting Kit-8 Assay

Cell viability was calculated using a Cell Counting Kit-8 (CCK-8) assay (Dojindo, Tokyo, Japan) according to the manufacturer’s protocol. Briefly, cells were cultured in 96-well plates to a density of 5 × 10^3^ cells per well, exposed to 4% sevoflurane for 6 h, and then incubated with CCK-8 solution (10 μl/well) for 20–30 min. Absorbance at 450 nm was measured using a Tecan microplate reader (Spark 10M), and cell viability was calculated as follows: cell viability (%) = [(As − Ab)/(Ac − Ab) × 100%. As, Ac, and Ab were measured in the sevoflurane-treated, control, and blank groups, respectively.

### Caspase-1 Assay

To detect pyroptotic cell death, BV2 cells were cultured on glass coverslips and treated with sevoflurane. Caspase-1 activity was measured using a FAM-fluorescent labelled inhibitors of caspase (FLICA) caspase-1 detection kit according to the manufacturer’s protocol. FLICA probes bind covalently to active caspase enzymes. The FAM-FLICA working solution was mixed with culture medium at a ratio of 1:30 v/v and added directly to adherent cells. The cells were incubated at 37°C for 60 min, with gentle mixing every 20 min to disperse the reagent. After the culture medium had been removed carefully, the cells were incubated with 1× Apoptosis Wash Buffer at 37°C for 10 min to wash away surplus FAM-FLICA. This step was repeated three times. To observe nuclear morphology, cells were incubated with Hoechst 33,342 (blue DNA binding dye) for 5 min at 37°C. To distinguish between apoptosis and necrosis, the cells were stained with propidium iodide (PI). Cells were then observed using a fluorescence microscope (Leica DMi8, Heidelberg, Germany). FAM-FLICA has an optimal excitation wavelength of 488–492 nm and peak emission at 515–535 nm. Images were analyzed using ImageJ software.

### Western Blotting

Cells cultured at 37°C with 5% CO_2_ were washed with phosphate-buffered saline (PBS) three times, resuspended in radioimmunoprecipitation assay (RIPA) buffer containing phenylmethylsulfonyl fluoride (PMSF) and phosphatase inhibitors, and incubated at 4°C for 30 min. After centrifugation at 12,000 rpm and 4°C for 15 min, the supernatant was collected, mixed with sodium dodecyl sulfate (SDS) protein loading buffer, and incubated at 95°C for 10 min. After 8% or 12% SDS–polyacrylamide gel electrophoresis, the samples were transferred to PVDF membranes, blocked with 5% skim milk powder at 20°C–25°C, shaken for 2 h, and incubated with primary antibodies at 4°C shaken overnight. Following this, the membranes were washed thrice with TBST buffer for 10 min and incubated with secondary antibodies at room temperature for 1 h. Protein bands were visualized and quantified using ECL reagent (Thermo Fisher Scientific, Waltham, MA, United States) with a chemiluminescence imaging system (ClinX Sciences, Shanghai, China).

### Immunofluorescence Staining

For the *in vitro* experiments, adherent BV2 cells plated on coverslips in six-well plates were treated with or without sevoflurane for 6 h, washed three times with PBS, and fixed with 4% polyformaldehyde for approximately 30 min. The cells were then permeabilized with 1% Triton X-100 for 20 min, blocked with 2% bovine serum albumin at room temperature for 1 h, and incubated with anti-ASC (Cell Signaling, #67824, 1:300) and anti-IBA1 (Thermo Fisher Scientific, MA5-27726, 1:300) antibodies at 4°C overnight. The cells were then incubated with 488 (Beyotime, A0423, 1:500) and cyc-conjugated (Beyotime, A0521, 1:500) secondary antibodies for 1 h at 37°C. Nuclei were stained using DAPI. Cells were observed directly using a fluorescence microscope (Leica DMi8).

For the *in vivo* experiments, fresh hippocampal tissues from sevoflurane-treated APP/PS1 mice were fixed with 4% polyformaldehyde. Frozen sections (10 μm) were processed for standard immunofluorescence staining. After being thawed for 30 min, the sections were washed with PBS seven times, blocked with 2% donkey serum at 20°C–25°C for 1 h, and incubated overnight at 4°C with the cleaved-GSDMD (1:100 dilution) and Iba1 (1:800) primary antibodies. The sections were then washed with PBS seven times (7 min each) and incubated with biotin-conjugated secondary antibodies (Alexa Fluor 488-labelled goat anti-rabbit IgG, Cy3-labelled goat anti-mouse IgG, 1:500) at room temperature for 1 h. Nuclear staining was performed using Hoechst, and fluorescent images were captured using a confocal microscope (Leica).

### Statistical Analysis

All experiments were repeated independently at least three times, and data were expressed as the mean ± standard error (SE). Significant differences between different groups were analyzed using Student’s *t*-tests using Origin 9.0 software (OriginLab, Northampton, MA, United States). *p*-Values of <0.05 were considered statistically significant.

## Results

### Sevoflurane Induces Pyroptosis and Tau Pathology in APP/PS1 Mice

**FIGURE 1:**
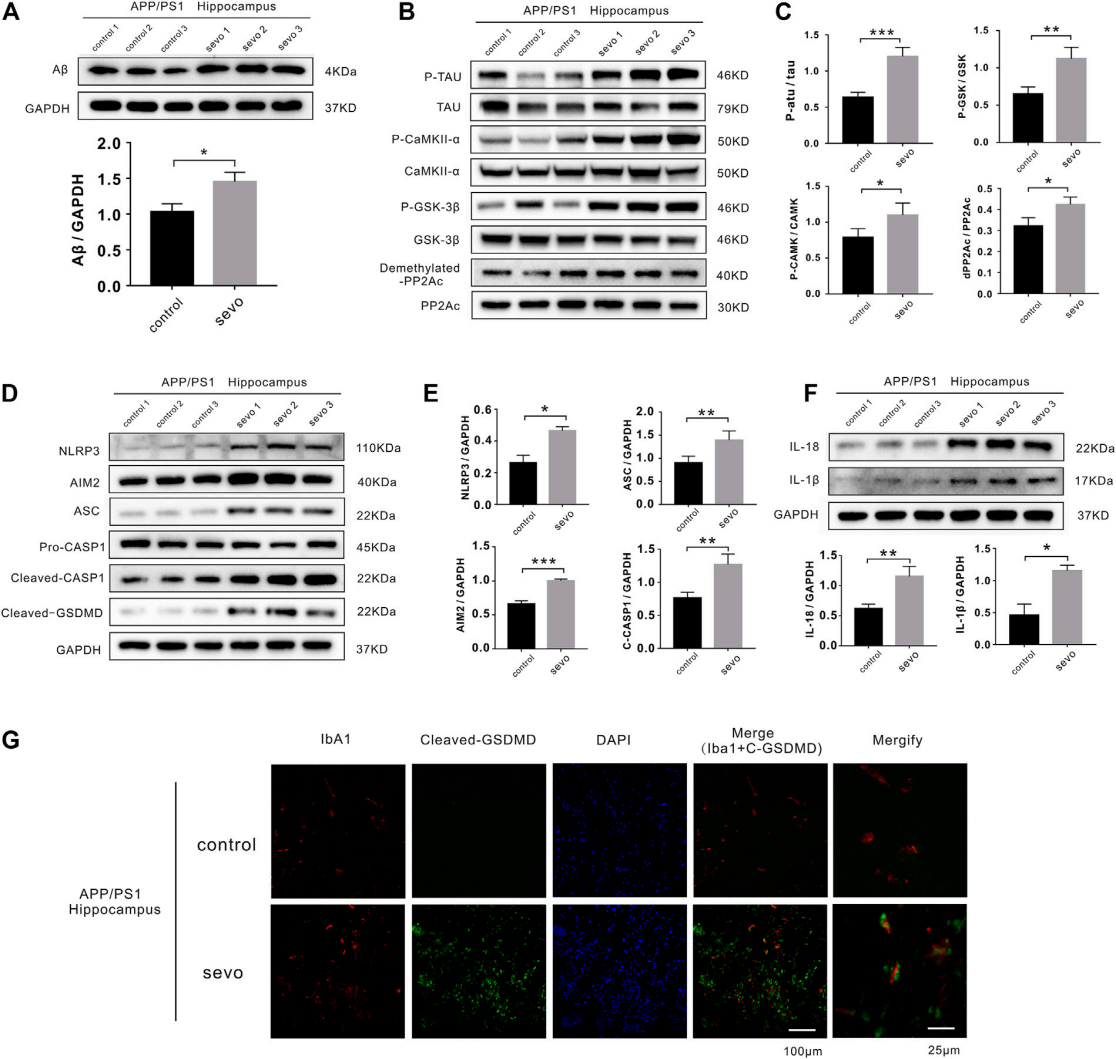
Sevoflurane induces pyroptosis and activates tau pathology in APP/PS1 mice. **(A)** Representative immunoblot analysis of Aβ protein expression in control or sevoflurane-treated APP/PS1 mice using GAPDH as an internal control. **(B)** Immunoblot analysis of APP/PS1 mouse hippocampi stained for phosphorylated tau (p-tau), total tau, total PP2A subunit C, demethylated PP2A subunit C, p-CaMKII-*α*, total CaMKII-*α*, GSK-3*β* phosphorylated (p-GSK-3*β*), and total GSK-3*β*. **(C)** Quantification of kinases and phosphatases in B (*n* = 6). **(D)** Immunoblot analysis of NLRP3, AIM2, ASC, pro-caspase-1, cleaved caspase-1, and cleaved gasdermin D (GSDMD). **(E)** Quantification of proteins in D using GAPDH as an internal control (*n* = 6). **(F)** Immunoblot analysis and quantification of interleukin-1*β* and 18 in hippocampi after sevoflurane treatment. **(G)** Immunofluorescence staining of Iba1, cleaved-GSDMD, and DAPI. Scale bar: 100 μm. ***p < 0.001, **p < 0.01, *p < 0.05.

In AD and other tauopathies, tau and prion deposition and extension occur in a similar manner. Previous studies have reported that sevoflurane can induce tau phosphorylation in brain tissue and tau trafficking from neurons to microglia, leading to cognitive impairment. To determine whether 3% sevoflurane altered phosphorylated tau (p-tau) and Aβ levels in APP/PS1 mice, we performed a Western blotting analysis on hippocampus samples. P-tau and Aβ levels were significantly increased in the sevoflurane group (1.9- and 1.4-fold, respectively; Figures 1A,B). Tau phosphorylation is regulated by a primary phosphatase (PP2A) and other kinases. Interestingly, phosphorylated CaMKII-*α* and GSK-3*β* kinases were upregulated in the hippocampi of sevoflurane-treated mice (1.4- and 1.7-fold, respectively) alongside inactive phosphatase PP2A (dPP2Ac/PP2Ac; Figures 1B,C).

In addition, cleaved caspase-1 and ASC were upregulated in the sevoflurane group (1.7- and 1.6-fold, respectively), suggesting NLRP3 inflammasome and AIM2 activation (1.7- and 1.5-fold, respectively). Consistently, the levels of cleaved GSDMD, an indicator of pyroptosis, were increased in the sevoflurane group, confirming pyroptosis (Figures 1D,E). We also detected higher levels of IL-1β and IL-18 secretion (2.5- and 1.8-fold, respectively) due to neuroinflammatory and degenerative processes (Figure 1F).

To examine whether sevoflurane affected microglial activation and pyroptosis in APP/PS1 mice, we stained hippocampus sections for the microglia-specific marker, Iba1, as well as cleaved GSDMD. Notably, there were considerably more microglia and significantly higher levels of cleaved GSDMD detected in the hippocampi of sevoflurane-treated APP/PS1 mice than in the control APP/PS1 mice (Figure 1G). The high degree of overlap between Iba1 and GSDMD fluorescence indicated that sevoflurane induces pyroptosis in the hippocampus of APP/PS1 mice, predominantly within microglia. To detect cognitive impairment in the APP/PS1 mice, we performed Morris water maze experiments and found that sevoflurane-treated mice displayed poorer navigation and spatial skills than the control mice (Supplementary Figure S1). The above experiments proved that sevoflurane induced pyroptosis and tau pathology in APP/PS1 mice leading to a cognitive disorder.

### Sevoflurane Induces Caspase-1-Dependent Pyroptosis in BV2 Microglia

**FIGURE 2:**
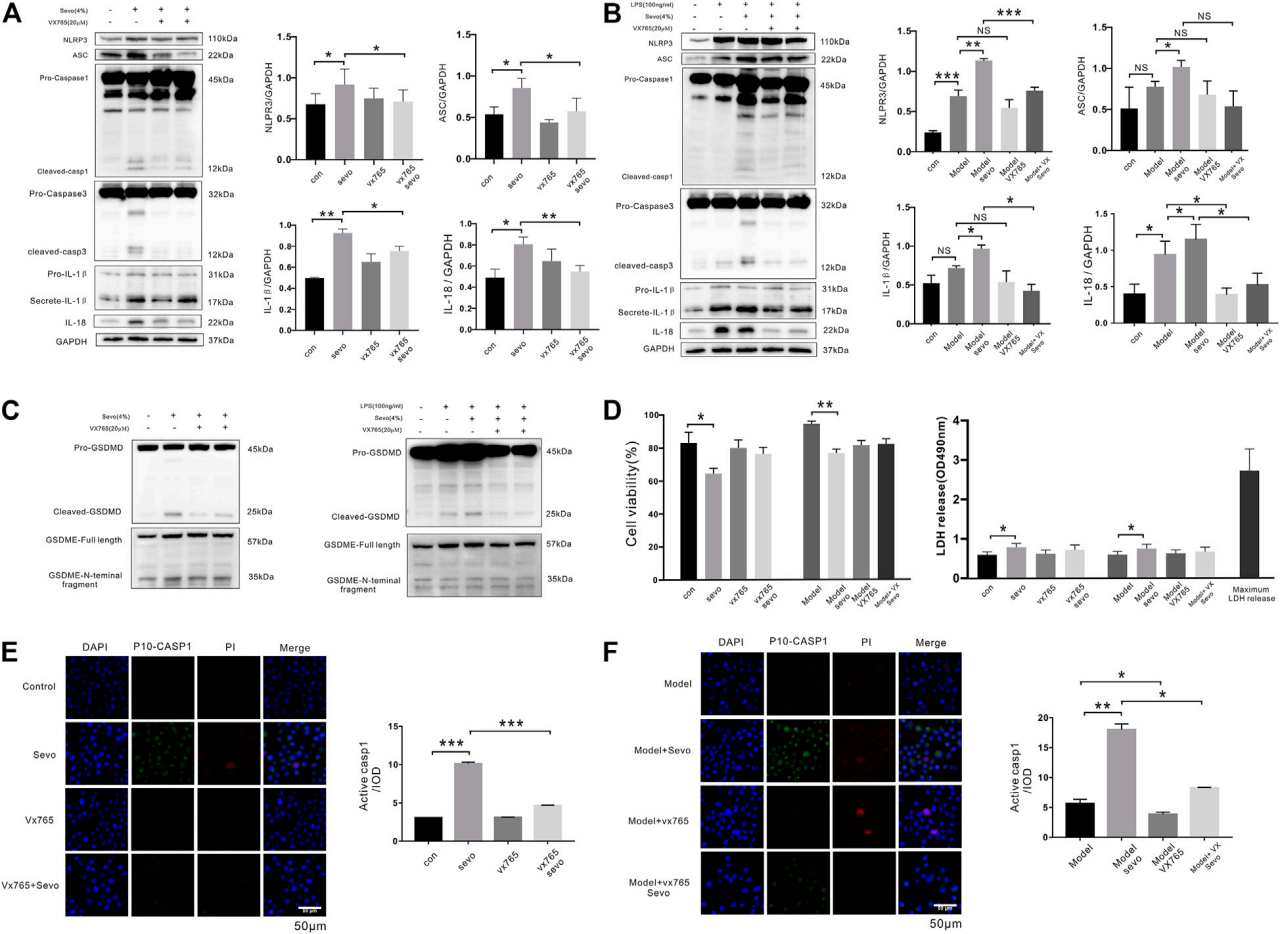
Sevoflurane promotes caspase-1-dependent pyroptosis in BV2 microglia **(A)**. Cleaved caspase-1 and caspase-3, IL-1β, and IL-18 levels were detected by Western blotting analysis in BV2 microglia exposed to 4% sevoflurane for 6 h **(B)**. Western blotting analysis of caspase-1, caspase-3, IL-1*β*, and IL-18 induced by sevoflurane in BV2 cells pretreated with 100 ng/ml of lipopolysaccharide (LPS). **(C)** Pyroptosis-related proteins (gasdermin D (GSDMD) and gasdermin E (GSDME)) were detected by Western blotting analysis induced by sevoflurane in BV2 cells. **(D)** Cell Counting Kit-8 (CCK-8) and lactate dehydrogenase (LDH) release assays of cell viability and LDH activity in BV2 cells. **(E)**. FAM-FLICA caspase-1 assays of cleaved caspase-1 (green), propidium iodide (PI) (red), and DAPI (blue) in BV2 cells. Scale bar: 50 μm. Cleaved caspase-1 fluorescence intensity was calculated as the iod. **(F)**. FAM-FLICA caspase-1 assays of cleaved caspase-1 (green) in BV2 cells. Scale bar: 50 μm. ***p < 0.001, **p < 0.01, *p < 0.05.

To determine whether sevoflurane causes caspase-1-induced pyroptosis *in vitro*, we treated BV2 microglia with 4% sevoflurane. Interestingly, sevoflurane activated the NLRP3 and ASC pathways (1.4- and 1.6-fold, respectively), increased active cleaved caspase-1 and caspase-3 levels, and potentiated IL-1β and IL-18 secretion (1.9- and 1.6-fold, respectively) in BV2 microglia (Figure 2A). The cells in the model group were pretreated with LPS (100 ng/ml) for 12 h. Similar effects were also induced by sevoflurane when the BV2 microglia were pretreated with LPS (Figure 2B). To verify the occurrence of sevoflurane-induced pyroptosis, we only observed cleavage of pro-GSDMD after 6 h of sevoflurane treatment in both untreated and LPS pretreated cells, while cleavage of pro-gasdermin E (pro-GSDME) was not changed (Figure 2C). Conversely, when BV2 microglia were pretreated for 12 h with VX-765, a nontoxic caspase-1 small molecule inhibitor (Mason et al., 2010), with pyroptosis-inducing effects of sevoflurane, including caspase-1 and GSDMD cleavage as well as IL-1β and IL-18 secretion, was inhibited (Figures 2A–C).

Next, we detected the effect of sevoflurane on cell viability using CCK-8 and LDH release assays and found that sevoflurane treatment for 6 h slightly reduced the viability of microglia as compared with the control cells (Figure 2D). Although the number of cleaved caspase-1-positive cells was significantly higher in BV2 microglia exposed to sevoflurane for 6 h, regardless of LPS pretreatment, few PI-stained cells were observed following sevoflurane treatment (Figures 2E,F). Taken together, these results suggest that sevoflurane induces caspase-1-mediated microglial pyroptosis that can be significantly inhibited by VX-765.

### Sevoflurane Induces Caspase-1-Dependent Pyroptosis by Activating the NLRP3-ASC Pathway in BV2 Microglia

**FIGURE 3:**
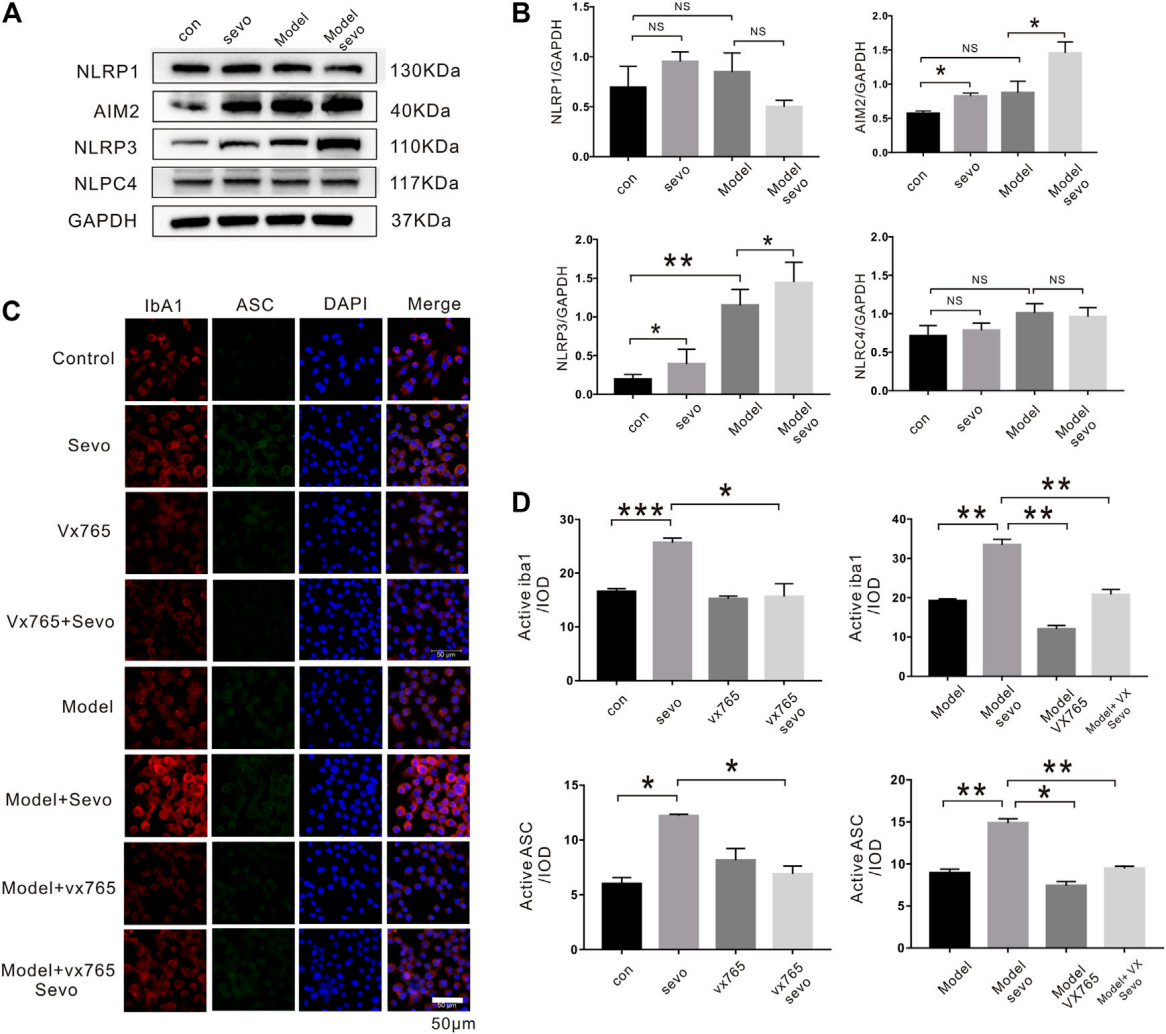
Sevoflurane exposure induces caspase-1-dependent pyroptosis by activating the NLRP3-ASC pathway **(A)**. Immunoblot analysis of NLRP1, AIM2, NLRP3, and NLPC4 expression in BV2 cells treated with 4% sevoflurane for 6 h or control. **(B)**. Quantitative Western blotting analysis of NLRP1, AIM2, NLRP3, and NLPC4. **(C)**. Iba1, ASC, and DAPI staining in BV2 cells imaged under a microscope. **(D)**. Iba1 and ASC fluorescence intensity was calculated as the iod. ***p < 0.001, **p < 0.01, *p < 0.05.

The activation of the NLR family (NALP1, NLRP3, and NLRC4) and AIM2 plays crucial roles in the regulation of pyroptosis (Satoh et al., 2013); therefore, we examined whether the expression of these proteins was altered during sevoflurane-induced microglial pyroptosis. Although sevoflurane increased AIM2 and NLRP3 expression in BV2 microglia irrespective of pretreatment with LPS, the levels of NALP1 and NLRC4 were relatively unchanged (Figures 3A,B).

ASC is a downstream regulator of the NLR family that plays a crucial role in caspase-1 recruitment and inflammasome assembly (Goldmann et al., 2013). To explore the possible effect of ASC in sevoflurane-induced pyroptosis, we performed immunofluorescence assays, which revealed that sevoflurane significantly increased ASC levels compared with the control or model groups (2.0-fold in the sevo group and 1.85-fold in the model + sevo group). Moreover, sevoflurane treatment increased the expression of the microglia marker Iba1 (1.7-fold in the sevo group and 1.8-fold in the model + sevo group; Figures 3C,D). In brief, sevoflurane induced caspase-1-dependent pyroptosis by activating the NLRP3-ASC pathway in BV2 microglia.

### Tau-Related Kinases and Phosphatases Regulate the Effects of Sevoflurane in BV2 Microglia

**FIGURE 4:**
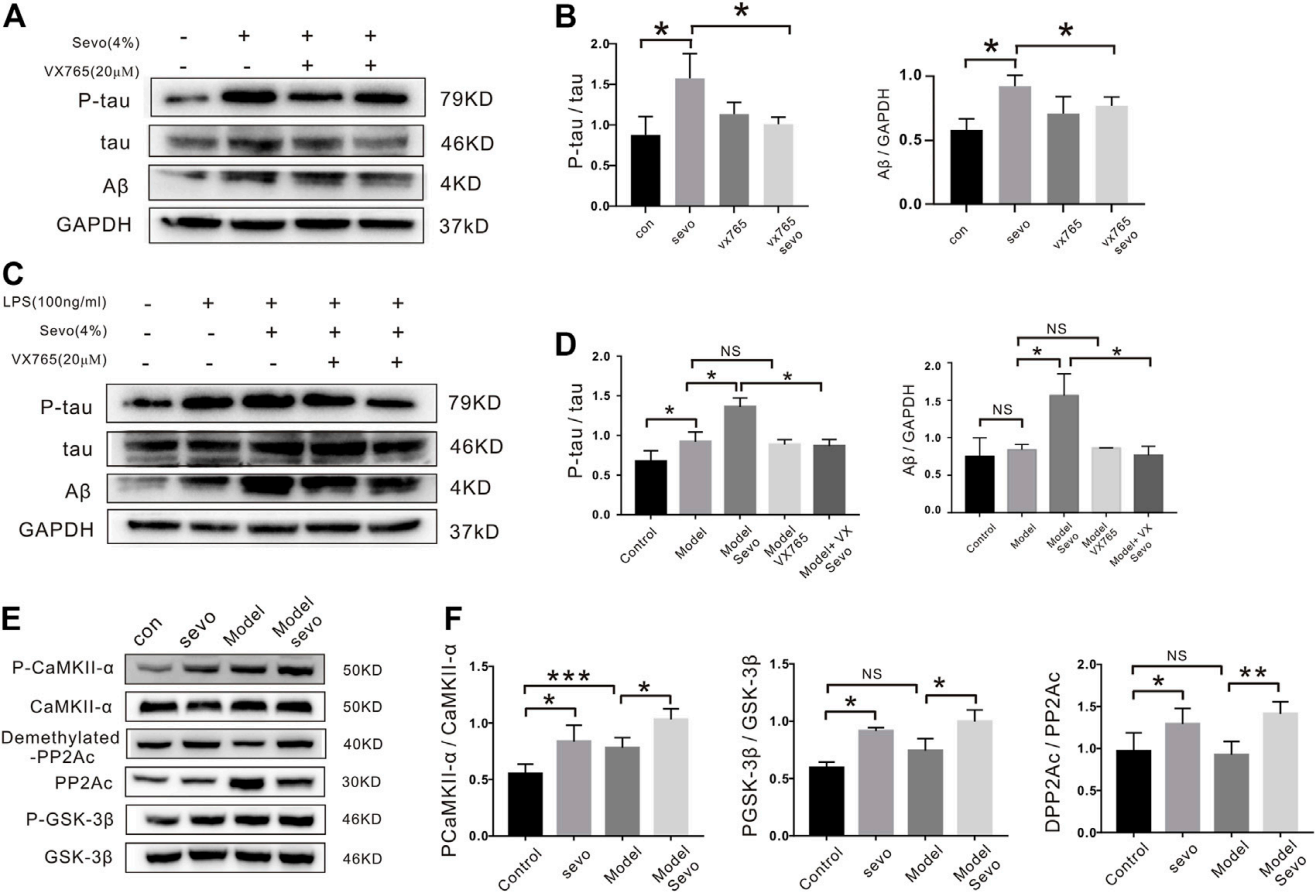
Tau-correlated kinases and phosphatases regulate the effects of sevoflurane in BV2 cells. **(A)** BV2 cells were treated with 4% sevoflurane for 6 h, and then p-tau and A*β* were detected by Western blotting. **(B)**. Quantitation of p-tau and A*β* band intensity using ImageJ software. (**C**) Western blotting analysis of p-tau and A*β* induced by sevoflurane in BV2 cells pretreated with 100 ng/ml of lipopolysaccharide (LPS). **(D)**. Quantitation of p-tau and A*β* band intensity using ImageJ software. **(** **E** **)** Expression of tau-correlated kinases and phosphatases (P-CaMKII, PP2A-D, and P-GSK-3β) as determined by Western blotting analysis. **(F)** Quantitation of P-CaMKII, PP2A-D and P-GSK band intensity using ImageJ software. ***p < 0.001, **p < 0.01, *p < 0.05.

Next, we examined the potential effects of tauopathy in sevoflurane-induced pyroptosis by analyzing cell protein samples and found that A*β* (1.6-fold) and p-tau (1.8-fold) levels were elevated after sevoflurane exposure (Figures 4A,B) compared with the control and LPS groups (Figures 4C,D). In addition, sevoflurane increased the phosphorylation activity of the CAMKIIα and GSK-3β kinases and upregulated inactive phosphatase PP2A (dPP2Ac/PP2Ac; Figures 4E,F). However, the VX-765 treatment significantly suppressed Aβ and p-tau accumulation in sevoflurane-treated cells (Figures 4A–D).

### NLRP3 Knockout Alleviates Sevoflurane-Induced Pyroptosis and Tau Pathology in BV2 Microglia

**FIGURE 5:**
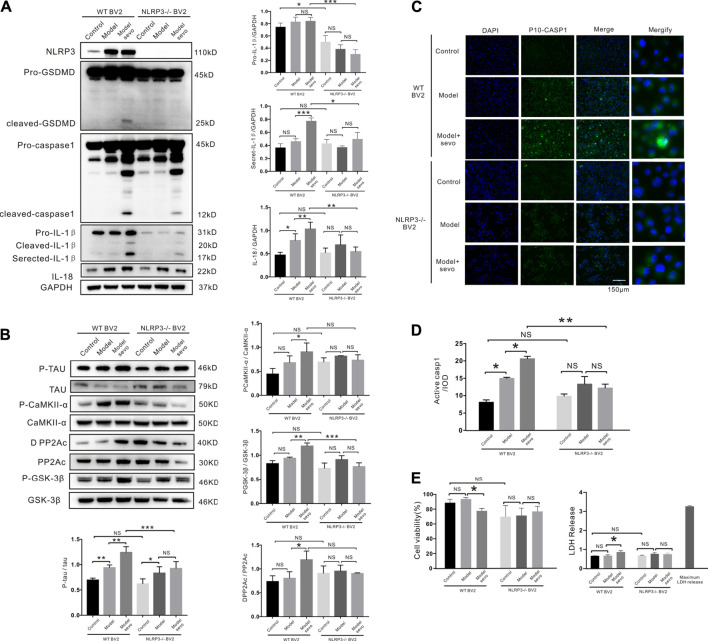
NLRP3 knockout alleviates sevoflurane-induced pyroptosis. **(A)** Western blotting analysis of cleaved gasdermin D (GSDMD), cleaved caspase-1, IL-1β, and IL-18 induced by sevoflurane in wild-type (WT) and NLRP3^−/−^ BV2 cells. Protein band intensity was quantified using ImageJ software. **(B)** Immunoblot analysis of p-tau, tau-correlated kinases, and phosphatases induced by sevoflurane in WT and NLRP3^−/−^ BV2 cells. **(C)** FAM-FLICA caspase-1 assay of cleaved caspase-1 (green) induced by sevoflurane in WT and NLRP3^−/−^ BV2 cells. Scale bar: 50 μm **(D)**. Quantitation of cleaved caspase-1 fluorescence intensity as iod. **(E)**. Lactate dehydrogenase (LDH) release and Cell Counting Kit-8 (CCK-8) assays of LDH activity and cell viability induced by sevoflurane in WT and NLRP3^−/−^ BV2 cells. ***p < 0.001, **p < 0.01, *p < 0.05.

To determine whether NLRP3 mediates sevoflurane-induced microglia pyroptosis, we knocked out NLRP3 gene using CRISPR/Cas9. As shown in Figure 5A, cleaved GSDMD forms were only detected in wild-type (WT) BV2 cells after sevoflurane treatment. Consistently, higher cleaved caspase-1 levels were measured in WT BV2 cells than in NLRP3^−/−^ BV2 cells (Figure 5A). The NLRP3 knockout cells also had lower pro-IL-1β levels than the WT cells, in spite of sevoflurane treatment (Figure 5A). NLRP3 deletion also significantly reduced p-tau levels in sevoflurane-treated groups (Figure 5B) and decreased D-PP2A induction and upregulation of the CAMKIIα and GSK-3β kinases by sevoflurane as compared with those in the control (Figure 5B).

To verify that NLRP3 silencing suppressed caspase-1 cleavage, we detected cleaved caspase-1-stained cells using FAM-FLICA caspase-1 assays. Compared with the WT BV2 cells, caspase-1 cleavage was dramatically decreased in NLRP3^−/−^ BV2 cells after sevoflurane exposure, highlighting the vital role of NLRP3 in sevoflurane-induced microglia pyroptosis (Figures 5C,D). Consistently, sevoflurane treatment did not affect the viability or LDH release of NLRP3^−/−^ BV2 cells compared with the control cells (Figure 5E). We also confirmed that NLRP3 knockout in APP/PS1 mice alleviated sevoflurane-mediated pyroptosis and tau phosphorylation (Supplementary Figure S2). Taken together, these results demonstrate that NLRP3 deletion prevents sevoflurane-increased toxicity, suggesting that NLRP3 is necessary for the induction of macroglia pyroptosis and tau pathology by sevoflurane.

### Caspase-1 Knockout Alleviates Sevoflurane-Induced IL-1β and IL-18 Release and Tau Pathology in BV2 Microglia

**FIGURE 6:**
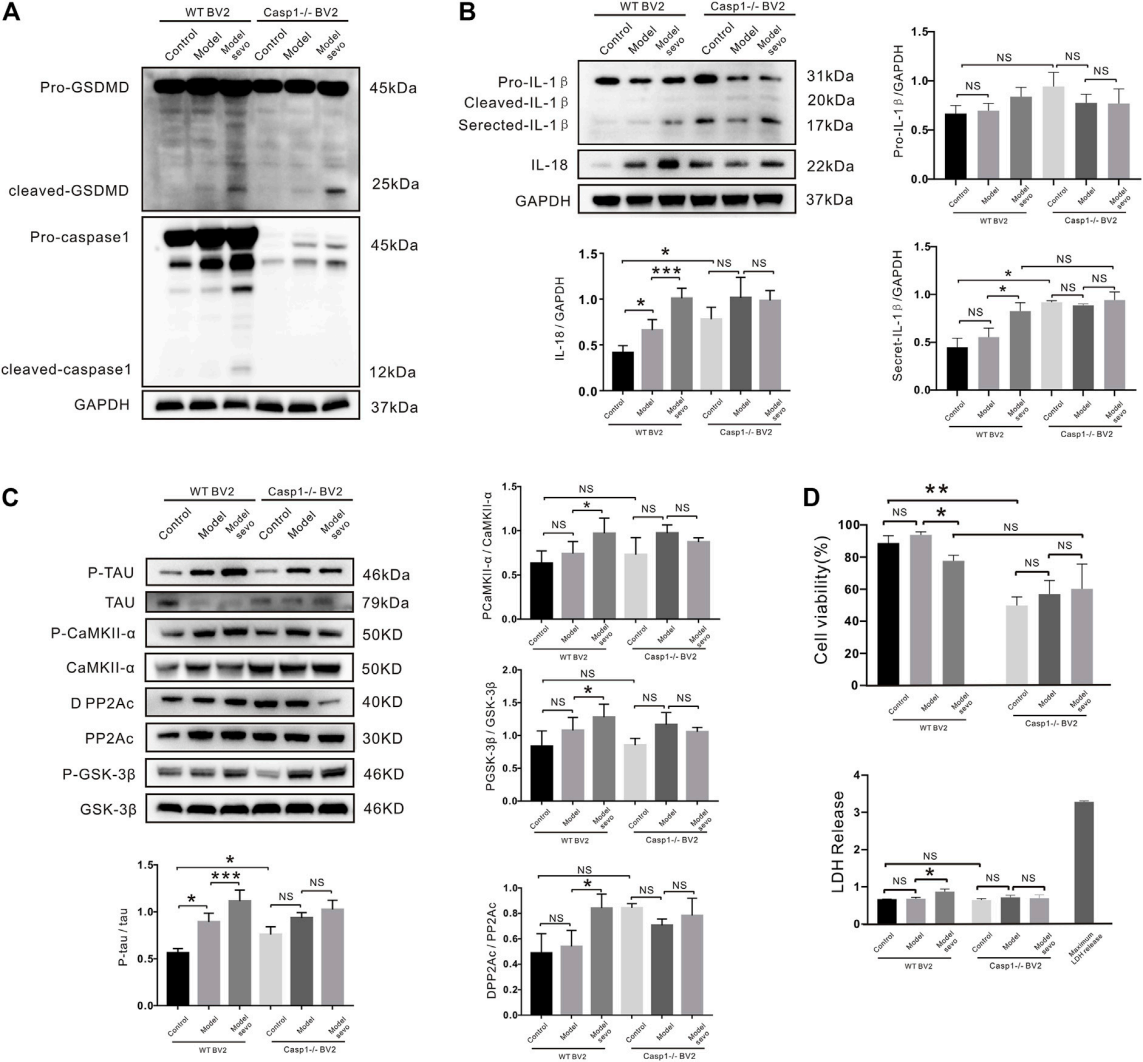
Casp1 knockout alleviates sevoflurane-induced pyroptosis and Alzheimer’s disease (AD) development. **(A)** Western blotting analysis of cleaved gasdermin D (GSDMD) and caspase-1 in wild-type (WT) and caspase-1^−/−^ BV2 cells treated with 4% sevoflurane for 6 h. **(B)** Immunoblot analysis of, secreted IL-18, secreted IL-1*β*, induced by sevoflurane in WT and caspase-1^−/−^ BV2 cells. Protein band intensity was quantified using ImageJ software. **(C)** P-tau and tau-related phosphatases were detected by Western blotting analysis. **(D)** Lactate dehydrogenase (LDH) release and Cell Counting Kit-8 (CCK-8) assays of LDH activity and cell viability in WT and NLRP3^−/−^ BV2 cells. ***p < 0.001, **p < 0.01, *p < 0.05.

To further elucidate the role of caspase-1 in sevoflurane-mediated pyroptosis, we knocked out caspase-1 in BV2 microglia that were treated with or without sevoflurane. GSDMD cleavage was significantly increased by sevoflurane treatment in caspase-1^−/−^ BV2 cells (Figure 6A). Secreted IL-1β and IL-18 were significantly upregulated as compared with the WT BV2 cells, while the secretion of IL-1β and IL-18 regulated by sevoflurane was indistinctive compared with the control group in caspase-1^−/−^ BV2 (Figure 6B). The upregulation of p-tau by sevoflurane was also abrogated in caspase-1^−/−^ BV2 cells (Figure 6C), and caspase-1 clearance antagonized the sevoflurane-induced activation/deactivation of kinases and phosphatases (Figure 6C). Although CCK-8 assays revealed a slight decrease in the viability of caspase-1^−/−^ BV2 cells compared with the WT BV2 cells, sevoflurane treatment did not significantly affect LDH release in caspase-1^−/−^ BV2 cells (Figure 6D). Collectively, these results confirm that caspase-1 knockout inhibits the proliferation of BV2 microglia and affects IL-1β secretion along with IL-18 and p-tau levels. Thus, our findings suggest that caspase-1 plays a role in sevoflurane-induced pyroptosis and establishes a link between pyroptosis and tauopathies.

## Discussion

Neuroinflammation is thought to play a critical role in AD (Dang et al., 2018); however, the underlying mechanism remains largely unclear. In this study, we demonstrated for the first time that clinical doses of sevoflurane can aggravate AD progression by inducing pyroptosis and tau pathology via a novel mechanism involving the activation of the NLRP3/ASC/caspase-1 pathway. Specifically, microglia exposed to sevoflurane subsequently underwent pyroptosis and displayed increased LDH release and reduced cell viability. Meanwhile, sevoflurane-treated mice and cells exhibited ASC speckling, caspase-1 and GSDMD cleavage, interleukin maturation and secretion, A*β* deposition, and tau pathology. Furthermore, the mice displayed cognitive dysfunction following sevoflurane treatment. These findings potentially explain the cross talk between pyroptosis and the pathology of AD and indicate that sevoflurane can promote the progression of AD by inducing pyroptosis.

Pyroptosis is a caspase-dependent form of programmed cell death that mainly occurs in immunocytes. Caspase-1 activity has been reported to promote IL-1β secretion via GSDMD-dependent pathways (Parajuli et al., 2013). Activated caspase-3 cleaves GSDME to release its N-terminal domain, which creates holes in the plasma membrane, leading to cell swelling, rupture, and death (Jiang et al., 2020). These processes in conjunction result in the induction of pyroptosis. Although we confirmed that sevoflurane treatment increased cleaved caspase-1 and caspase-3 levels, it only increased the cleavage of GSDMD, not GSDME. Therefore, sevoflurane appears to induce pyroptosis via caspase-1-mediated GSDMD cleavage.

Microglia are vital immunocytes in the central nervous system that exert many important functions during neuro-inflammatory responses. The concentrated proliferation and activation of microglia in the brain around A*β* plaques is a prominent feature of AD. Different external stimuli can induce the activation of two different phenotypic forms of microglia, namely, M1 and M2. We found that sevoflurane treatment induced the activation of M1-type microglia, which secrete pro-inflammatory factors such as TNF-α, IL-1β, and IL-6 (Supplementary Figure S3). Since NLRP-3/caspase-1/GSDMD axis-constituted pyroptosis mainly occurs in microglia rather than in neurons, we chose microglia for our study and demonstrated a high degree of overlap between Iba1 and GSDMD fluorescence in sevoflurane-treated hippocampus sections.

Sevoflurane treatment altered the expression of the NLR family (NALP1, NLRP3, and NLRC4) and AIM2 in microglia and activated the NLRP3 inflammasome and AIM2. Since active NLRP3 plays a vital role in caspase-1-related pyroptosis, we confirmed its role in sevoflurane-induced pyroptosis and found that deleting NLRP3 resulted in inhibition of pyroptosis by partially decreasing the cleavage of caspase-1 and GSDMD induced by sevoflurane. Although NLRP3 knockout decreased total IL-1β levels by approximately 50%, IL-18 levels were unchanged, possibly due to the existence of an unknown compensatory mechanism such as ASC-related inflammasomes (AIM). Previous studies have reported that tau activates the NLRP3 inflammasome and that intracerebral injection of fibrillar A*β*-containing brain homogenates can contribute toward tau pathology in an NLRP3-dependent manner (Broz, 2015; Flores et al., 2018). Here, we found that NLRP3 deletion reduced sevoflurane-induced tau hyperphosphorylation by affecting the interplay between tau kinases and phosphatases, which was consistent with previous findings (Choi et al., 2017).

Having demonstrated that pyroptosis occurs as a consequence of sevoflurane-mediated inflammation, we decided to explore whether caspase-1, the activation of which induces pyroptosis, affects the pathogenesis of tauopathies. Caspase-1 knockout reduced the viability of BV2 microglia, but not LDH release, and increased IL-1β levels, which not only affected the function of sevoflurane but also indicated that microglia-derived IL-1β causes tauopathy deterioration, as observed with other tau kinases (Bhaskar et al., 2010). Furthermore, caspase-1 knockout reduced the effects of sevoflurane on IL-1β and IL-18 secretion and p-tau while unexpectedly increasing GSDMD cleavage. Therefore, we speculate that caspase-1 deletion prevents normal microglia growth and activates a different pathway that promotes GSDMD cleavage. For instance, caspase-1 silencing has been shown to reduce the bacterial defense functions of MG-63 cells (Lima Leite et al., 2020). Our findings suggest that caspase-1 knockout reduces sevoflurane-increased pyroptosis and inflammation, potentially via the NLRP3/caspase-1 pathway; however, further studies are required to elucidate these mechanisms in more detail.

Although sevoflurane induced caspase-1-dependent pyroptosis by activating NLRP3 inflammasomes in both the WT and model groups, leading to inflammation and tau pathology, sevoflurane exerted stronger effects in the model group, supporting the hypothesis that anesthesia via inhalation may increase the risk of AD in those with a genetic predisposition (Xu et al., 2018; Xu et al., 2019). However, a few studies have reported that low-dose sevoflurane can enhance memory retention in rats (Callaway et al., 2012; Le Freche et al., 2012). These contradictory results may be explained by the heterogeneity of experimental design, such as the animal model/age, anesthetic formulation/dose, duration of exposure, and the period of cognitive experimentation and evaluation.

Finally, we demonstrated that the nontoxic caspase-1 small molecule inhibitor, VX-765 (Mason et al., 2010), inhibited sevoflurane-induced caspase-1-dependent pyroptosis in microglia while inhibiting IL-18 activity and IL-1β secretion and reducing tau phosphorylation and A*β* accumulation. These findings are consistent with existing evidence that VX-765 prevents A*β* protein deposition, reverses brain inflammation, and normalizes synaptophysin protein levels in the hippocampus of mice (Xu et al., 2019). Thus, VX-765 could be a promising drug for treating AD.

## Conclusion

This study is the first to report that clinical doses of sevoflurane can aggravate AD progression by inducing pyroptosis and tau pathology via the NLRP3/caspase-1/GSDMD pathway. In particular, sevoflurane directly activates caspase-1, leading to GSDMD cleavage and subsequent pyroptosis by selectively activating NLRP3-ASC and AIM2 inflammasomes. NLRP3 and caspase-1 KO further confirmed that the NLRP3/caspase-1 axis plays an important role in the induction of pyroptosis, Aβ deposition, and tau pathogenesis by sevoflurane. Furthermore, our findings suggest that VX-765 not only blocks sevoflurane-induced pyroptosis but also suppresses Aβ deposition and tau phosphorylation in microglia, suggesting that VX-765 could represent a novel therapeutic intervention for AD.

## Data Availability

The original contributions presented in the study are included in the article/Supplementary Material, Further inquiries can be directed to the corresponding author.

## Abbreviations

AD, Alzheimer’s disease; A*β*, *β*-amyloid; CCK-8, Cell Counting Kit-8; DMEM, Dulbecco’s modified Eagle’s medium; FBS, fetal bovine serum; IL-1β, interleukin-1β; GSDMD, gasdermin D; LPS, lipopolysaccharide; NLR, Nod-like receptors; PVDF, polyvinylidene fluoride; SDS, sodium dodecyl sulfate.
