# Supplementary figures and images for: Sevoflurane Aggravates the Progress of Alzheimer’s Disease Through NLRP3/Caspase-1/Gasdermin D Pathway (part 1 of 2)
Source: Front Cell Dev Biol. 2022 Jan 19;9:801422. doi: 10.3389/fcell.2021.801422 (PMC8807556; doi:10.3389/fcell.2021.801422)

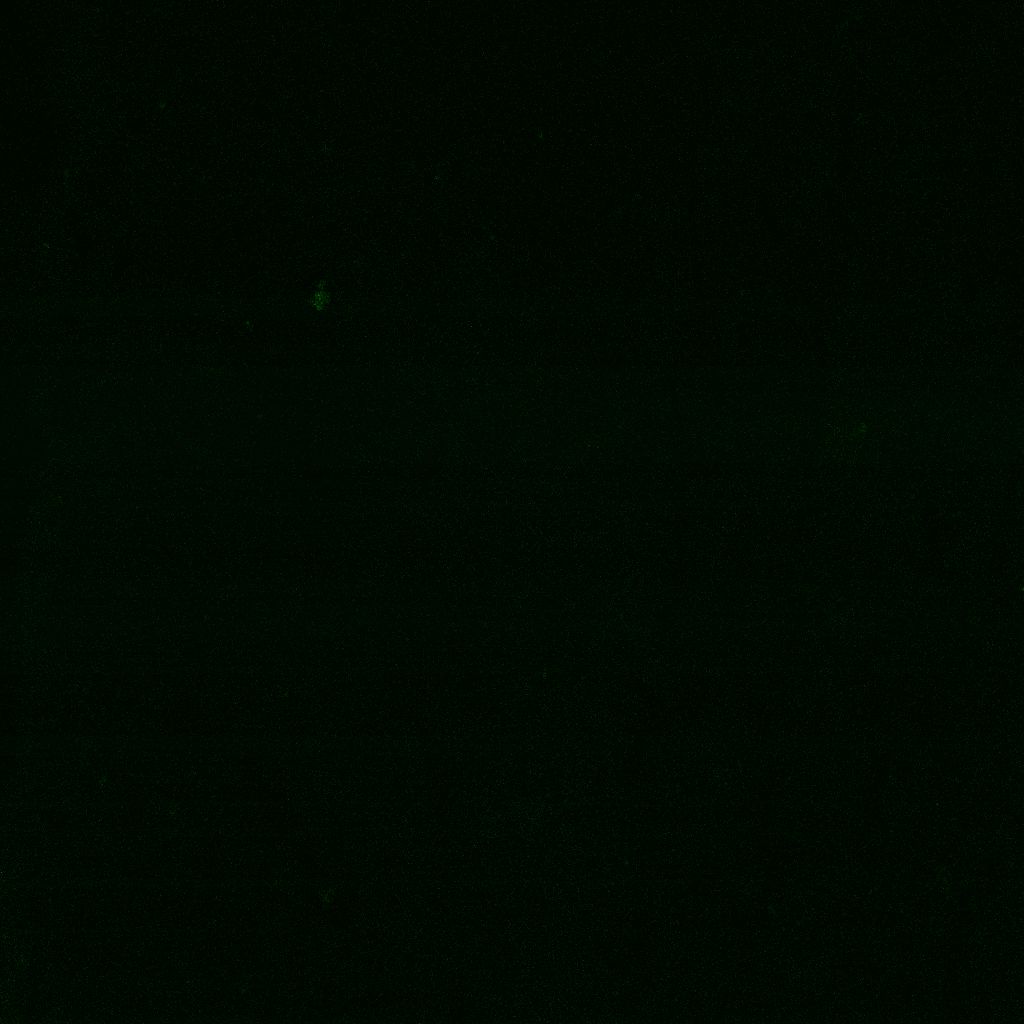

Supplement: Supplementary file 1 [file DataSheet3.ZIP › Original microscopy images1/Figure1/CON C-GSDMD.tif]

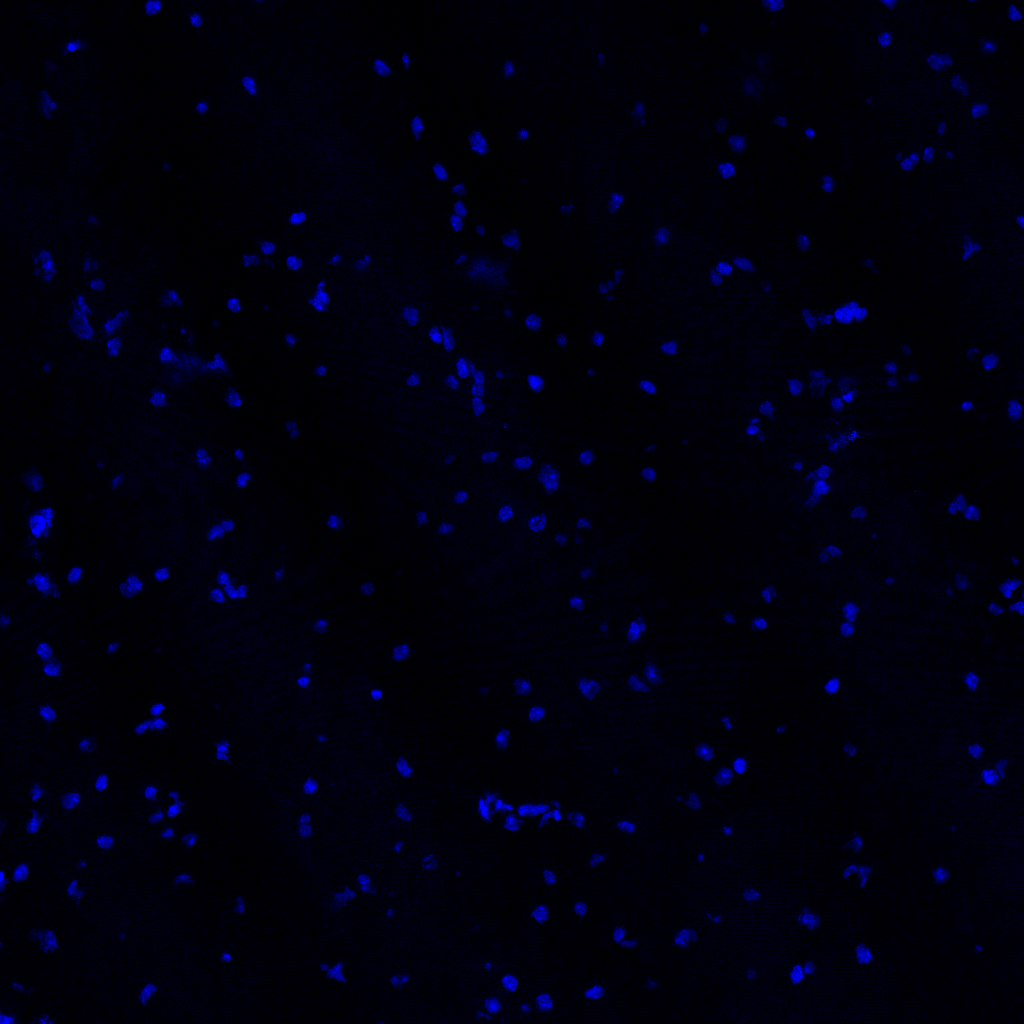

Supplement: Supplementary file 1 [file DataSheet3.ZIP › Original microscopy images1/Figure1/CON DAPI.tif]

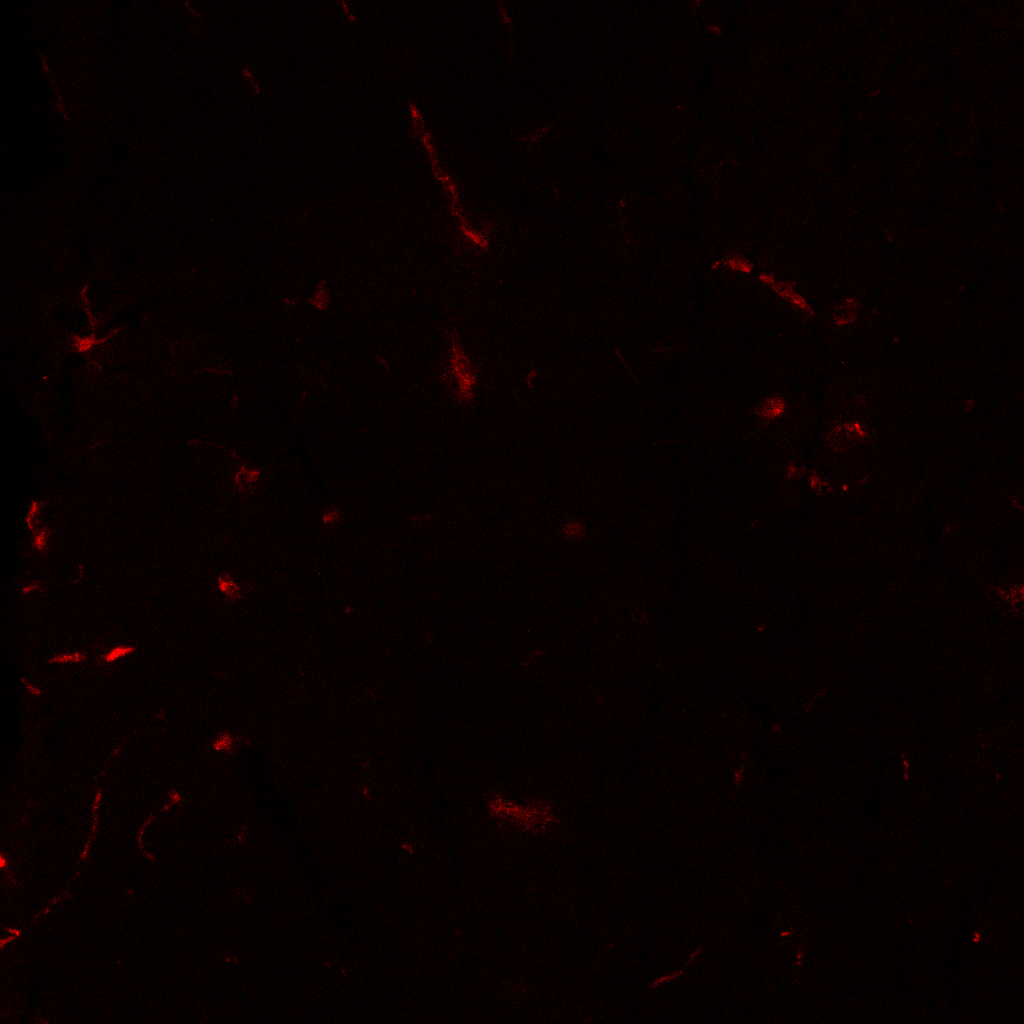

Supplement: Supplementary file 1 [file DataSheet3.ZIP › Original microscopy images1/Figure1/con Iba1.tif]

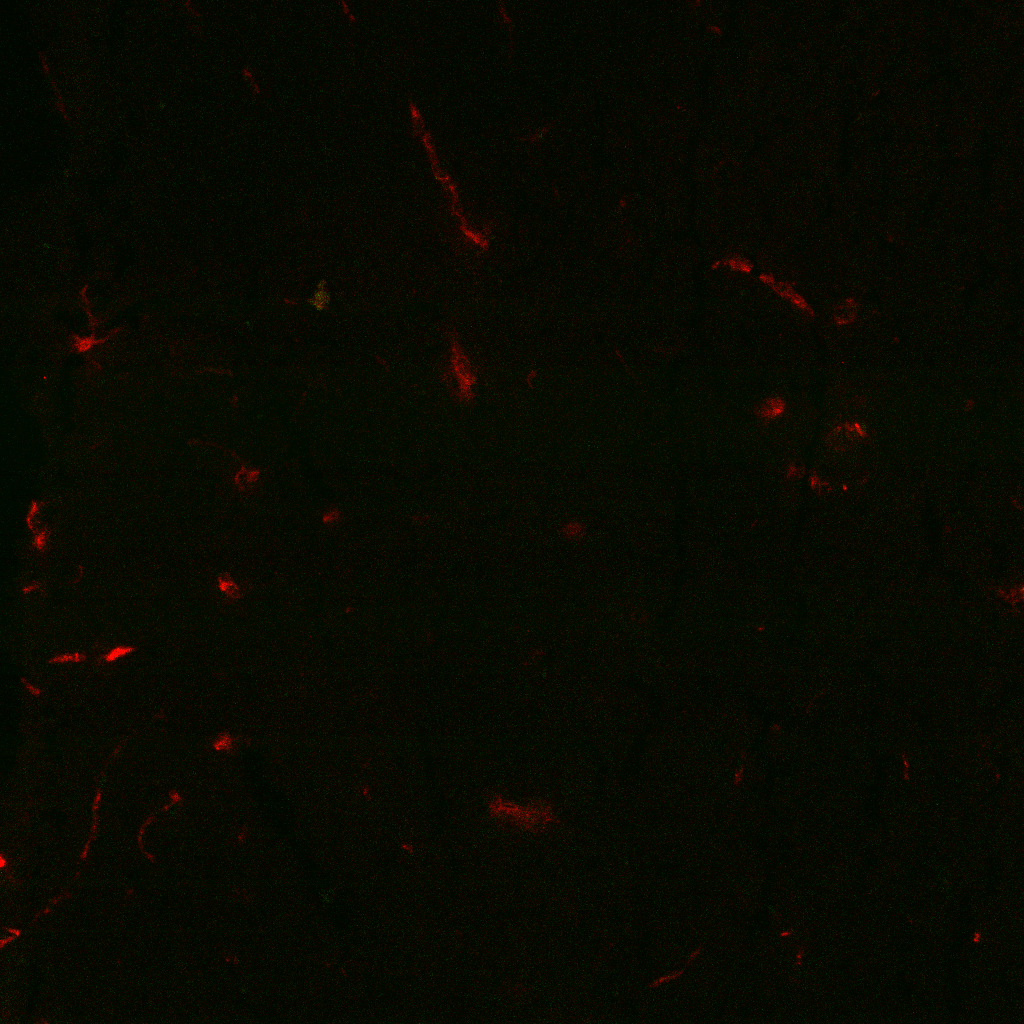

Supplement: Supplementary file 1 [file DataSheet3.ZIP › Original microscopy images1/Figure1/con merge.tif]

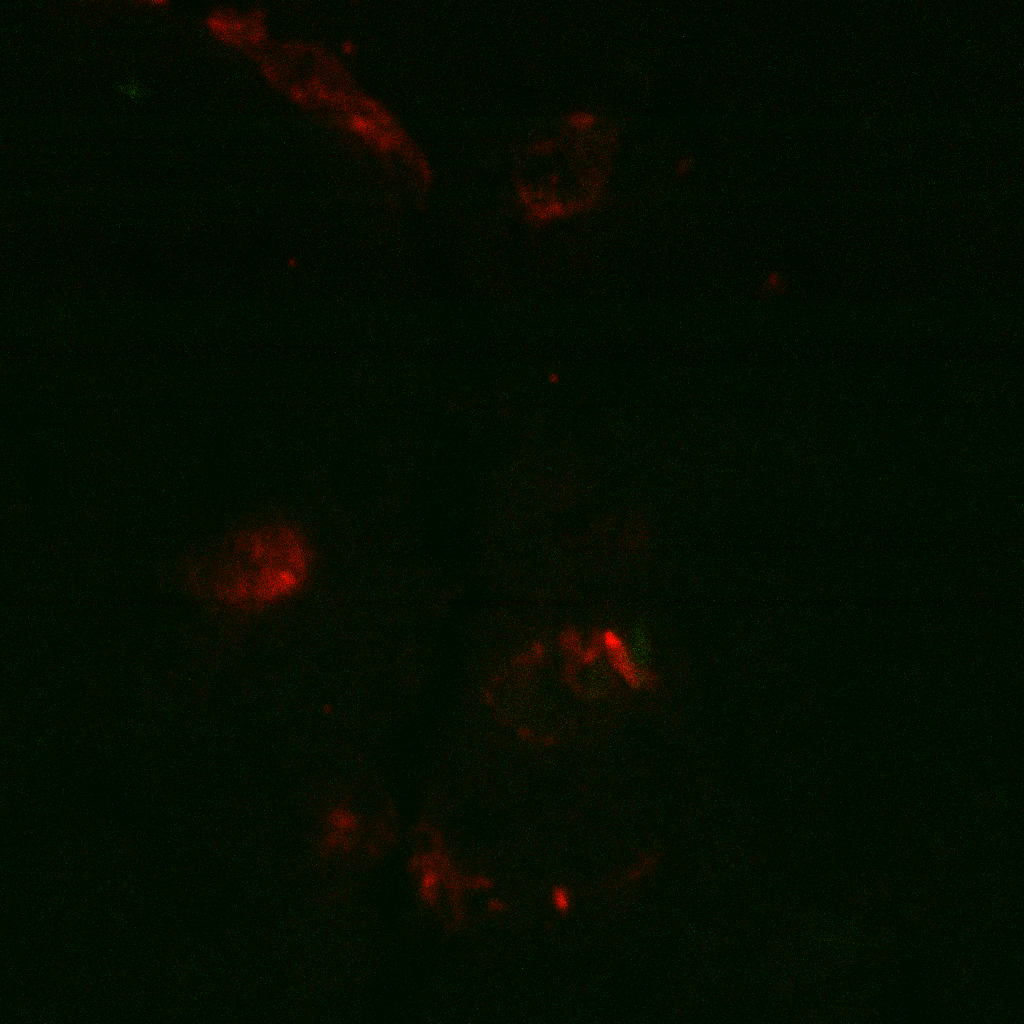

Supplement: Supplementary file 1 [file DataSheet3.ZIP › Original microscopy images1/Figure1/con mergify.tif]

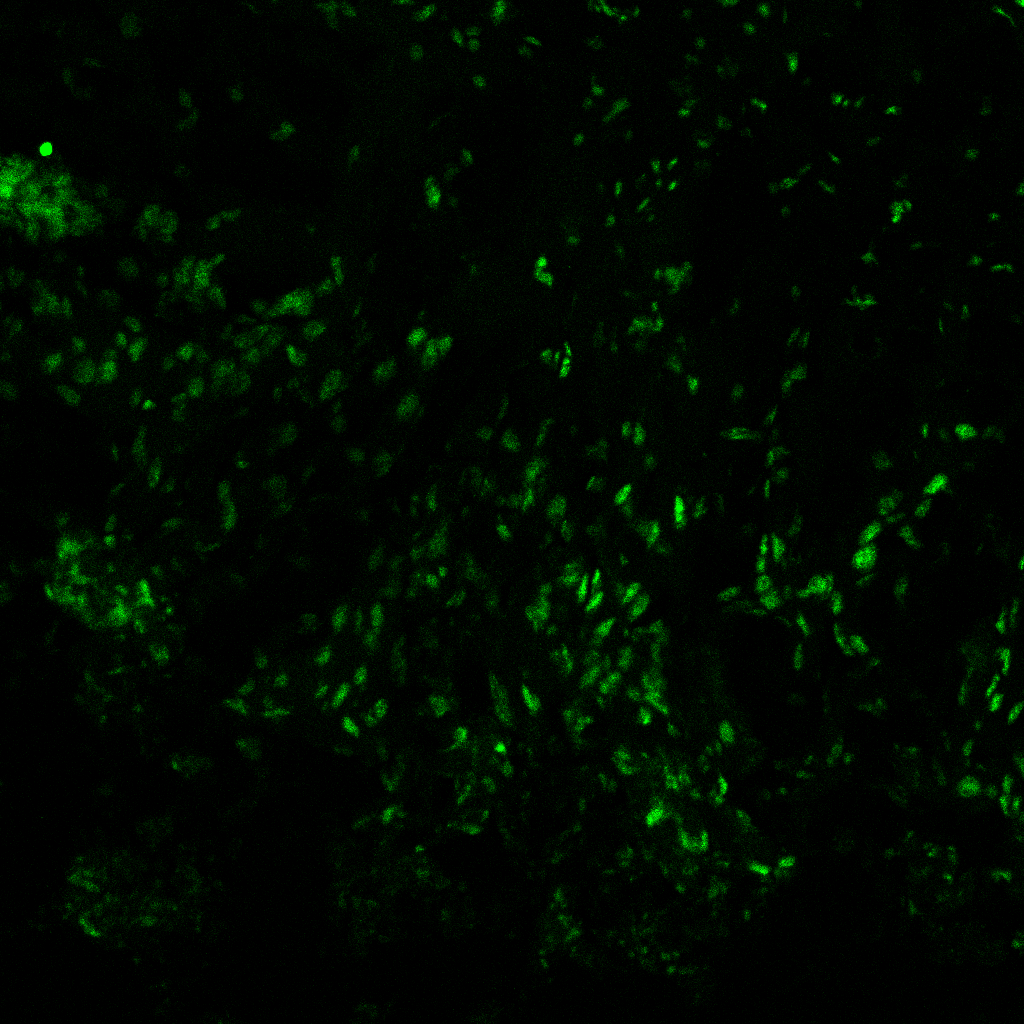

Supplement: Supplementary file 1 [file DataSheet3.ZIP › Original microscopy images1/Figure1/sevo C-GSDMD.tif]

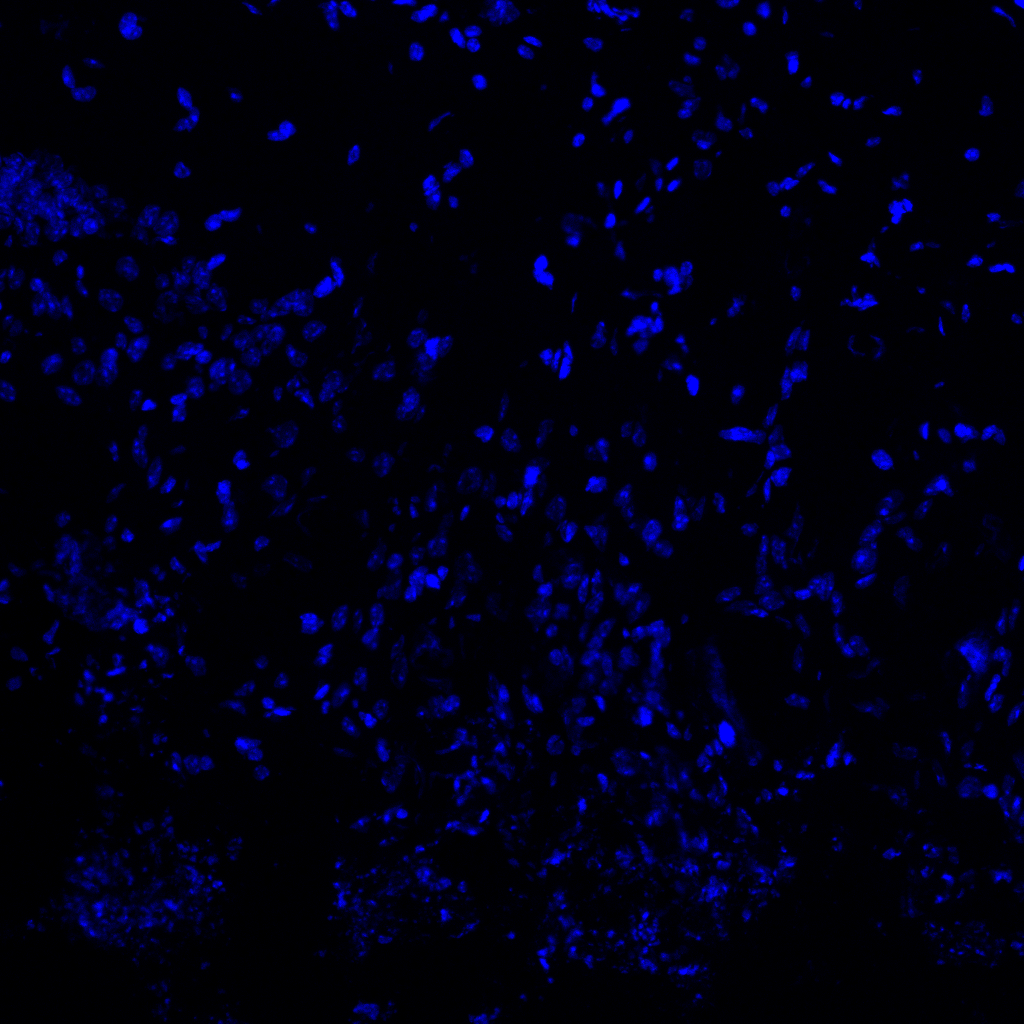

Supplement: Supplementary file 1 [file DataSheet3.ZIP › Original microscopy images1/Figure1/sevo DAPI.tif]

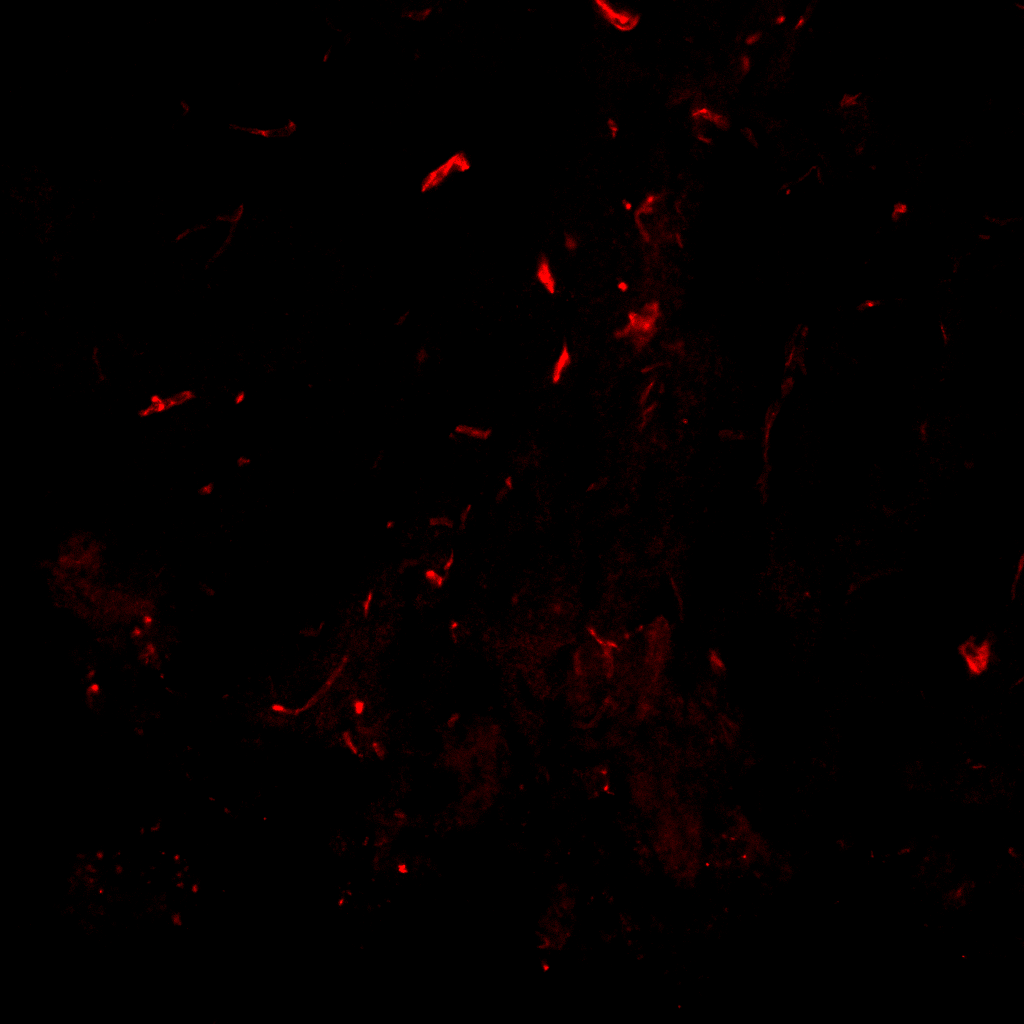

Supplement: Supplementary file 1 [file DataSheet3.ZIP › Original microscopy images1/Figure1/sevo Iba1.tif]

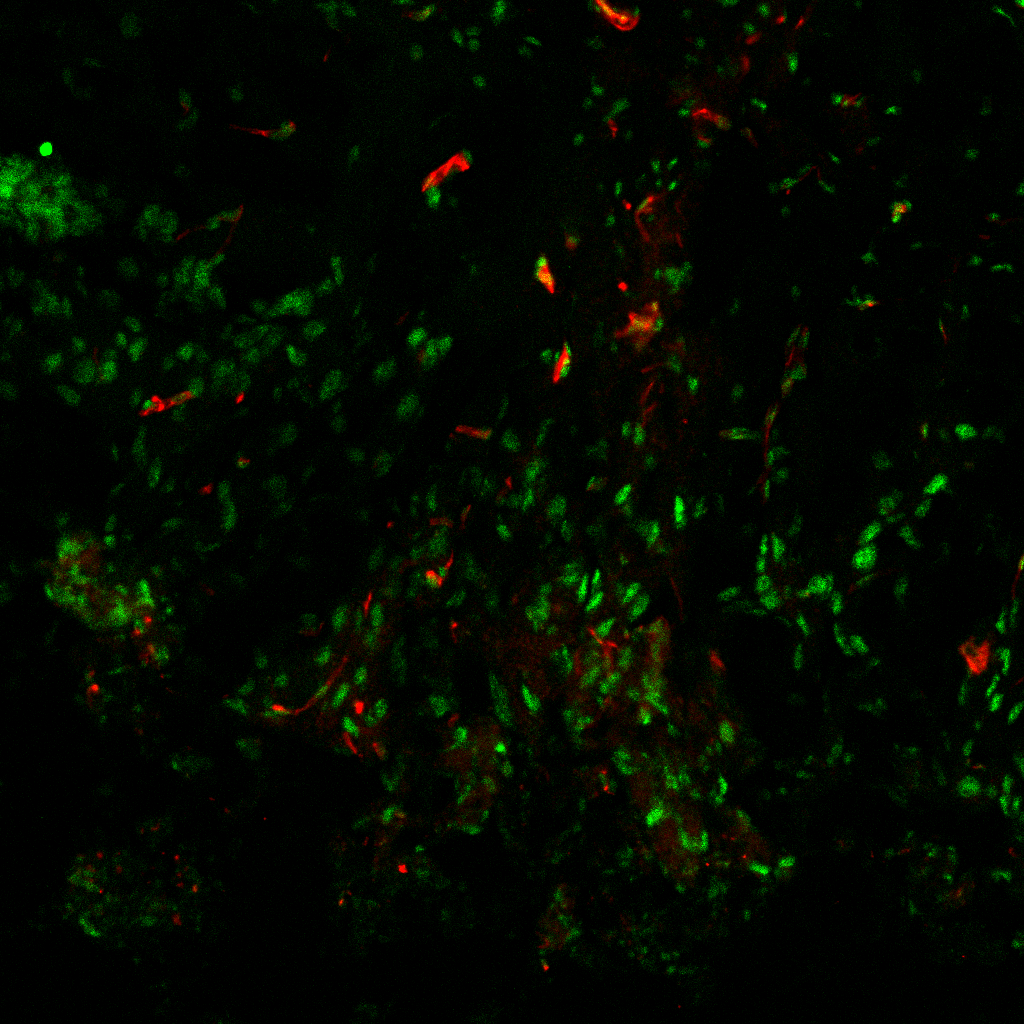

Supplement: Supplementary file 1 [file DataSheet3.ZIP › Original microscopy images1/Figure1/sevo merge.tif]

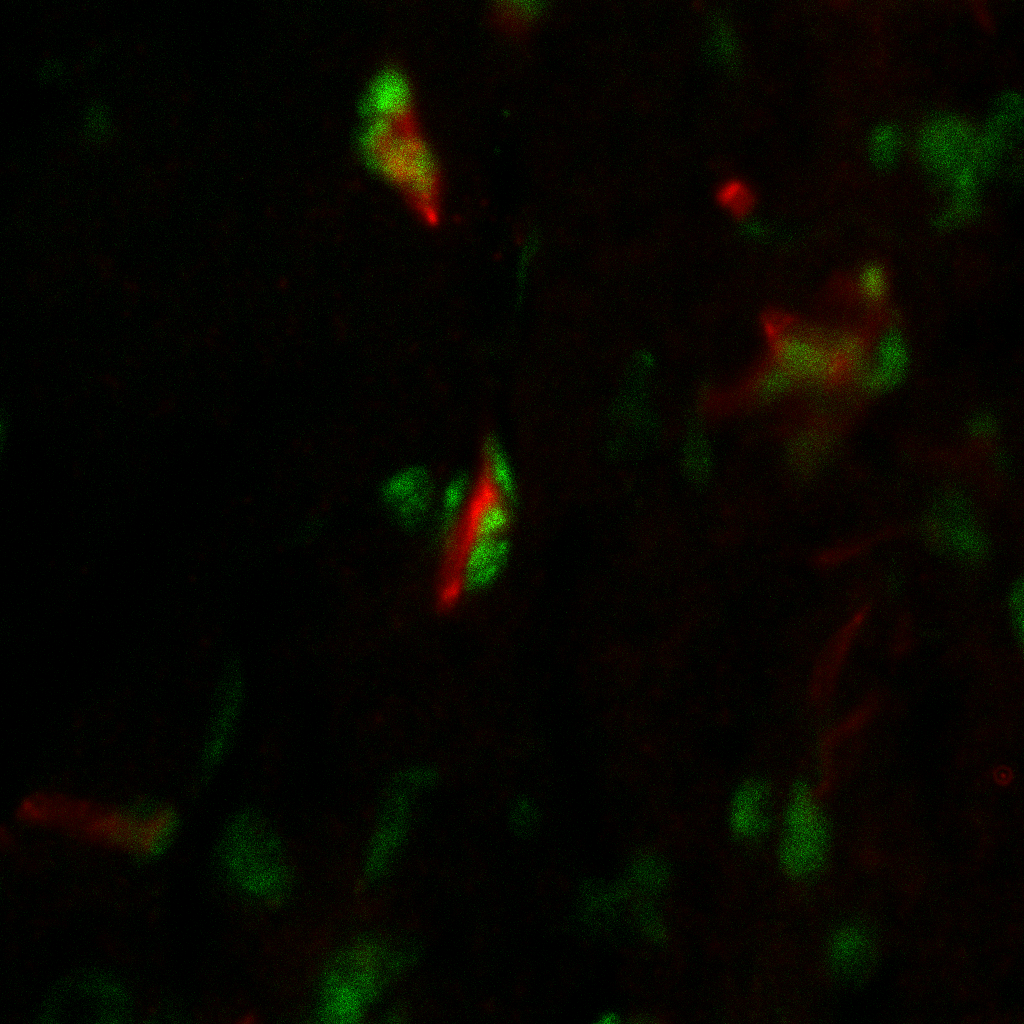

Supplement: Supplementary file 1 [file DataSheet3.ZIP › Original microscopy images1/Figure1/sevo mergify.tif]

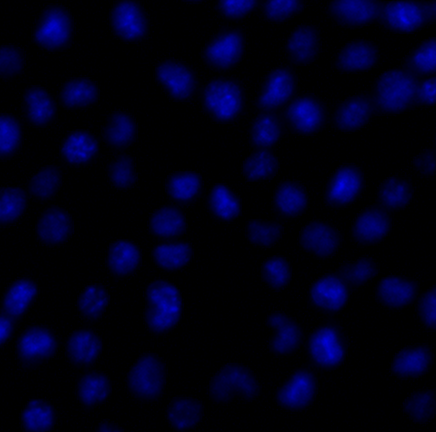

Supplement: Supplementary file 1 [file DataSheet3.ZIP › Original microscopy images1/Figure2/con DAPI.tif]

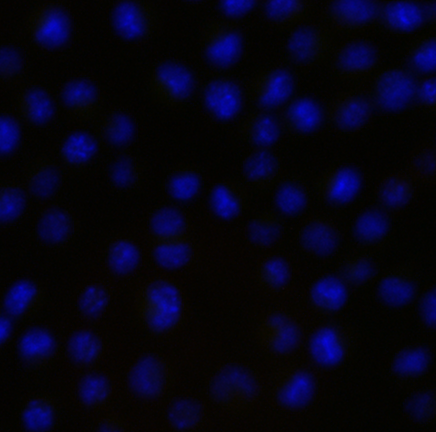

Supplement: Supplementary file 1 [file DataSheet3.ZIP › Original microscopy images1/Figure2/con merge.tif]

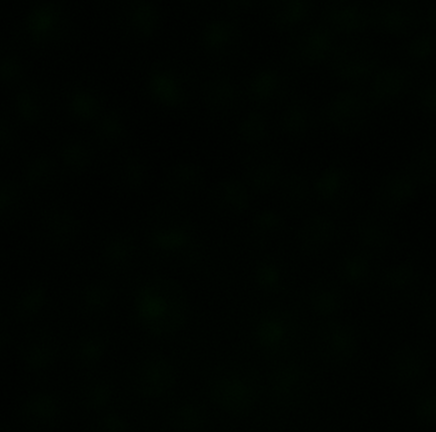

Supplement: Supplementary file 1 [file DataSheet3.ZIP › Original microscopy images1/Figure2/con P10-casp1.tif]

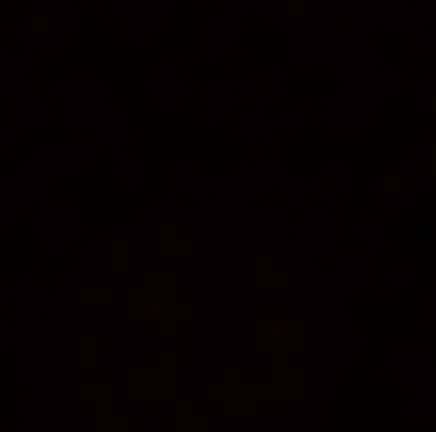

Supplement: Supplementary file 1 [file DataSheet3.ZIP › Original microscopy images1/Figure2/con PI.tif]

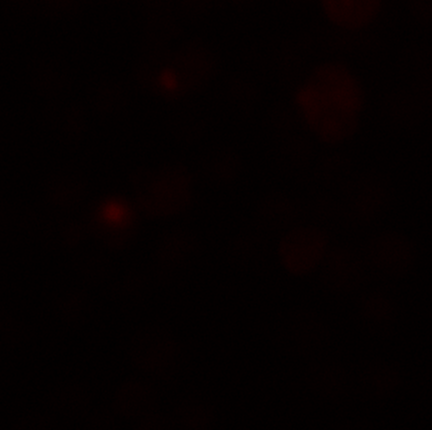

Supplement: Supplementary file 1 [file DataSheet3.ZIP › Original microscopy images1/Figure2/Model PI.tif]

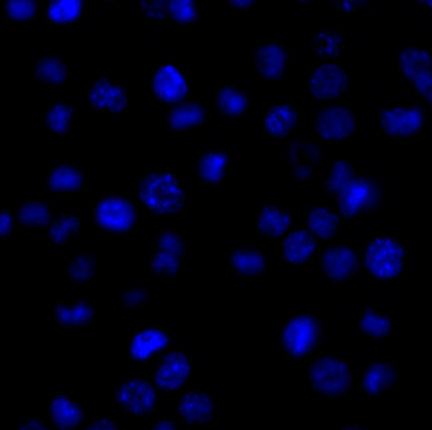

Supplement: Supplementary file 1 [file DataSheet3.ZIP › Original microscopy images1/Figure2/Model DAPI.tif]

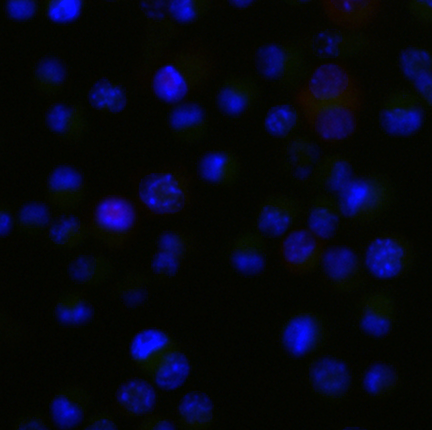

Supplement: Supplementary file 1 [file DataSheet3.ZIP › Original microscopy images1/Figure2/Model merge.tif]

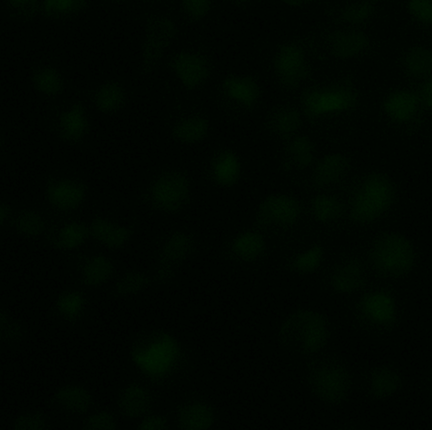

Supplement: Supplementary file 1 [file DataSheet3.ZIP › Original microscopy images1/Figure2/Model P10-casp1.tif]

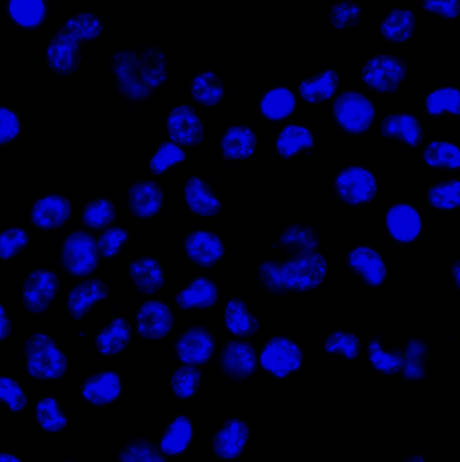

Supplement: Supplementary file 1 [file DataSheet3.ZIP › Original microscopy images1/Figure2/Model sevo DAPI.tif]

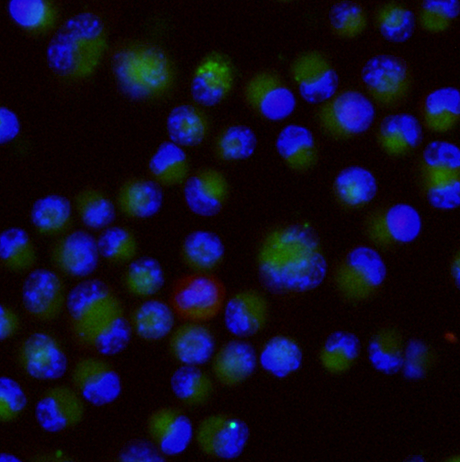

Supplement: Supplementary file 1 [file DataSheet3.ZIP › Original microscopy images1/Figure2/Model sevo merge.tif]

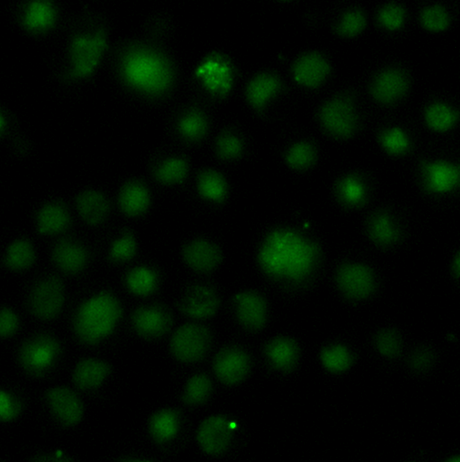

Supplement: Supplementary file 1 [file DataSheet3.ZIP › Original microscopy images1/Figure2/Model sevo P10-casp1.tif]

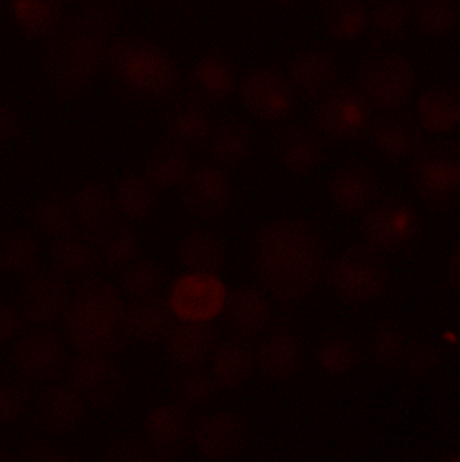

Supplement: Supplementary file 1 [file DataSheet3.ZIP › Original microscopy images1/Figure2/Model sevo PI.tif]

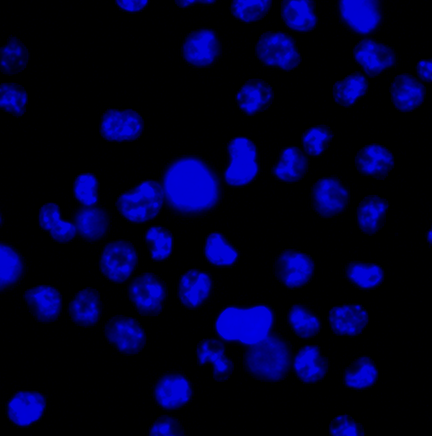

Supplement: Supplementary file 1 [file DataSheet3.ZIP › Original microscopy images1/Figure2/Model vx765 DAPI.tif]

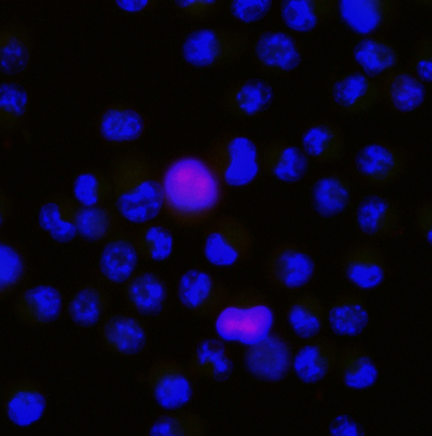

Supplement: Supplementary file 1 [file DataSheet3.ZIP › Original microscopy images1/Figure2/Model vx765 merge.tif]

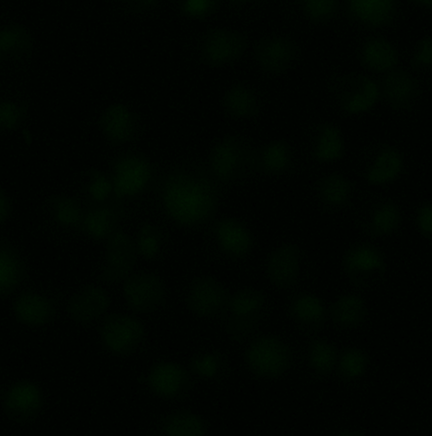

Supplement: Supplementary file 1 [file DataSheet3.ZIP › Original microscopy images1/Figure2/Model vx765 P10-casp1.tif]

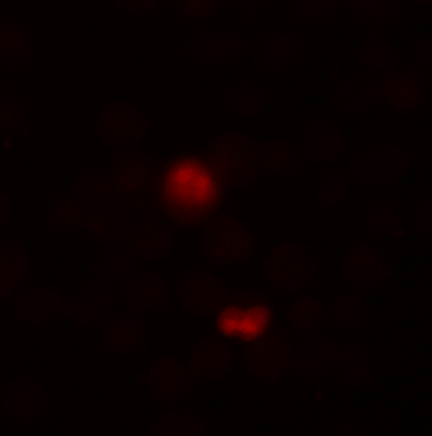

Supplement: Supplementary file 1 [file DataSheet3.ZIP › Original microscopy images1/Figure2/Model vx765 PI.tif]

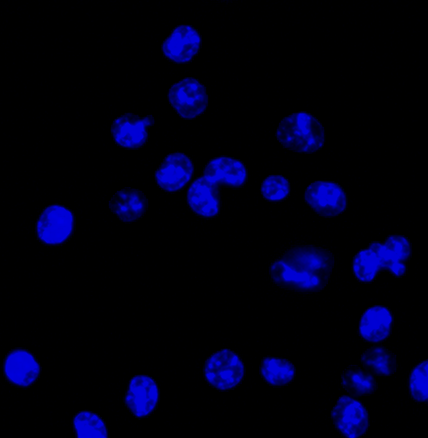

Supplement: Supplementary file 1 [file DataSheet3.ZIP › Original microscopy images1/Figure2/Model vx765 sevo DAPI.tif]

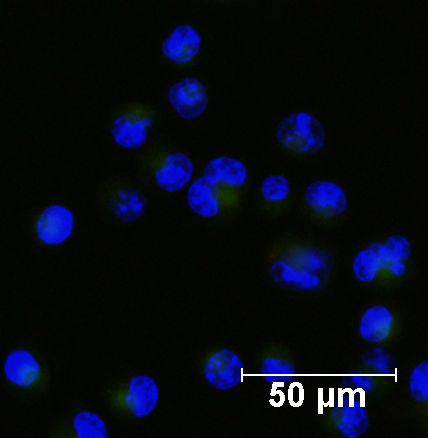

Supplement: Supplementary file 1 [file DataSheet3.ZIP › Original microscopy images1/Figure2/Model vx765 sevo merge.tif]

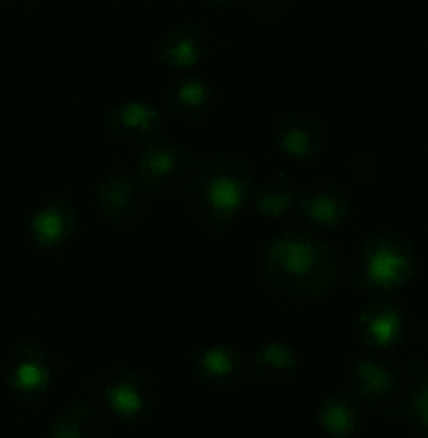

Supplement: Supplementary file 1 [file DataSheet3.ZIP › Original microscopy images1/Figure2/model vx765 sevo P10-casp1.tif]

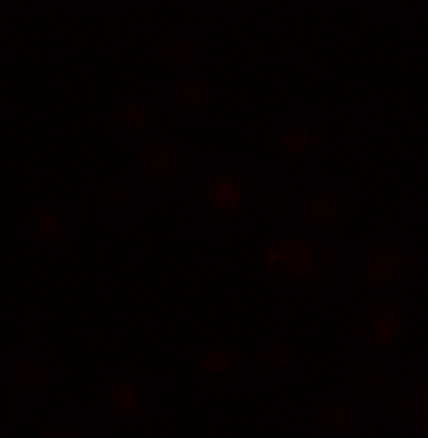

Supplement: Supplementary file 1 [file DataSheet3.ZIP › Original microscopy images1/Figure2/Model vx765 sevo PI.tif]

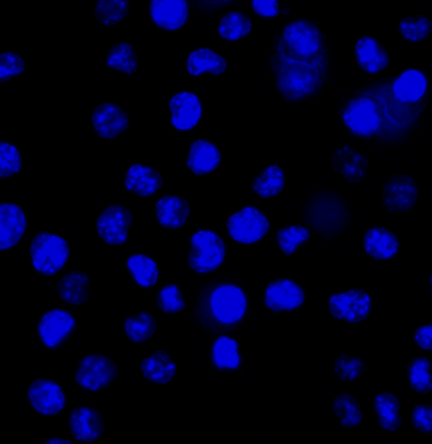

Supplement: Supplementary file 1 [file DataSheet3.ZIP › Original microscopy images1/Figure2/sevo DAPI.tif]

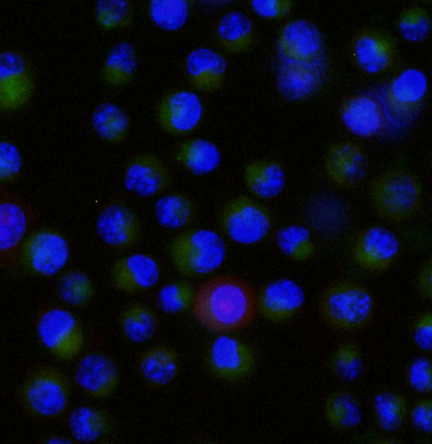

Supplement: Supplementary file 1 [file DataSheet3.ZIP › Original microscopy images1/Figure2/sevo merge.tif]

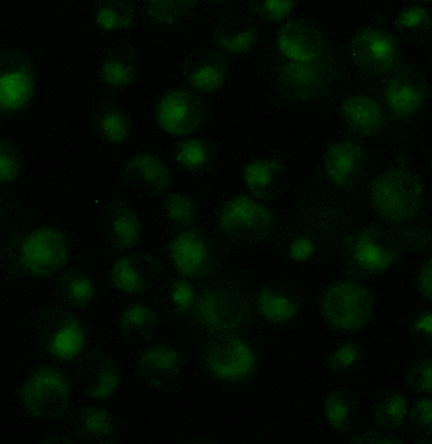

Supplement: Supplementary file 1 [file DataSheet3.ZIP › Original microscopy images1/Figure2/sevo P10-casp1.tif]

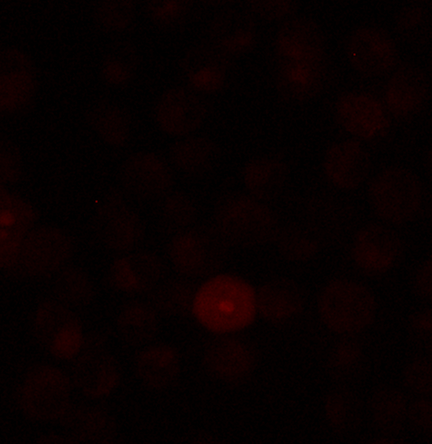

Supplement: Supplementary file 1 [file DataSheet3.ZIP › Original microscopy images1/Figure2/sevo PI.tif]

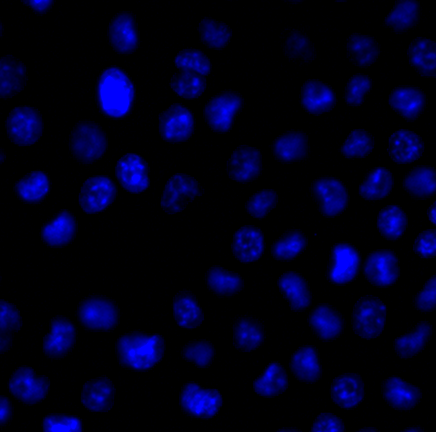

Supplement: Supplementary file 1 [file DataSheet3.ZIP › Original microscopy images1/Figure2/vx765 DAPI.tif]

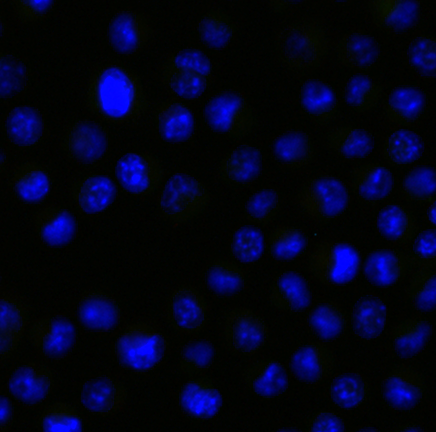

Supplement: Supplementary file 1 [file DataSheet3.ZIP › Original microscopy images1/Figure2/vx765 merge.tif]

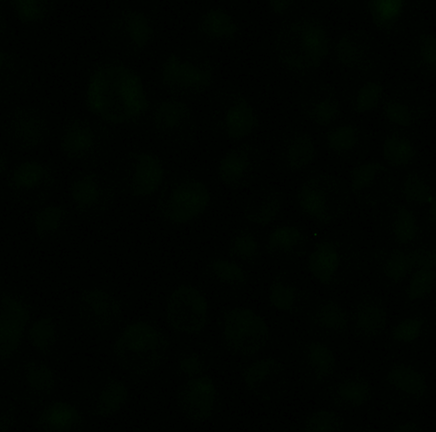

Supplement: Supplementary file 1 [file DataSheet3.ZIP › Original microscopy images1/Figure2/vx765 P10-casp1.tif]

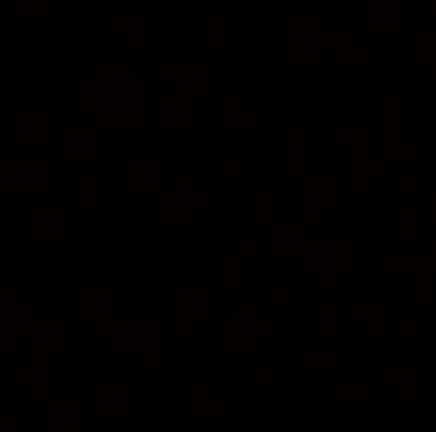

Supplement: Supplementary file 1 [file DataSheet3.ZIP › Original microscopy images1/Figure2/vx765 PI.tif]

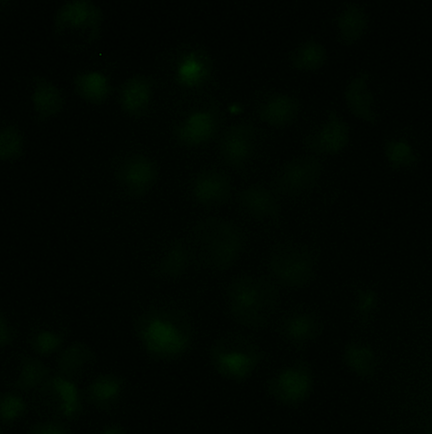

Supplement: Supplementary file 1 [file DataSheet3.ZIP › Original microscopy images1/Figure2/vx765 sevo P10-casp1.tif]

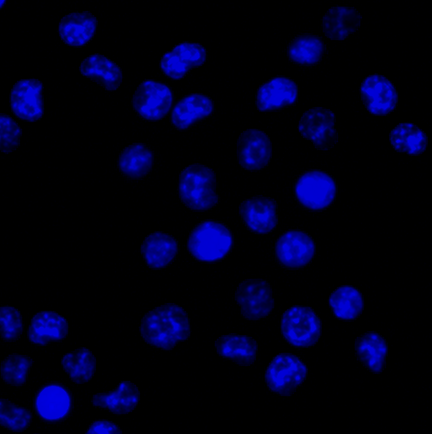

Supplement: Supplementary file 1 [file DataSheet3.ZIP › Original microscopy images1/Figure2/vx765 sevo DAPI.tif]

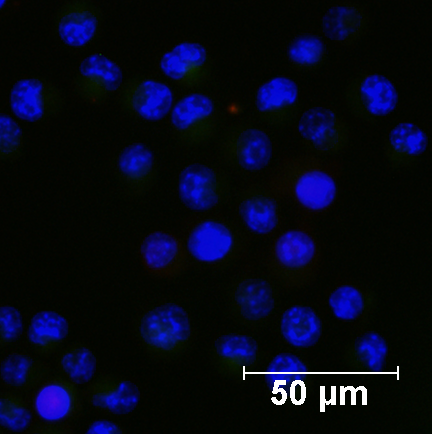

Supplement: Supplementary file 1 [file DataSheet3.ZIP › Original microscopy images1/Figure2/vx765 sevo merge.tif]

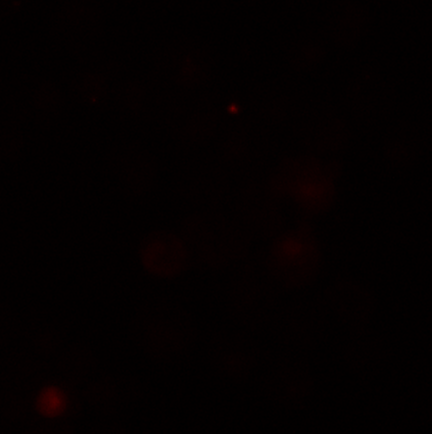

Supplement: Supplementary file 1 [file DataSheet3.ZIP › Original microscopy images1/Figure2/vx765 sevo PI.tif]

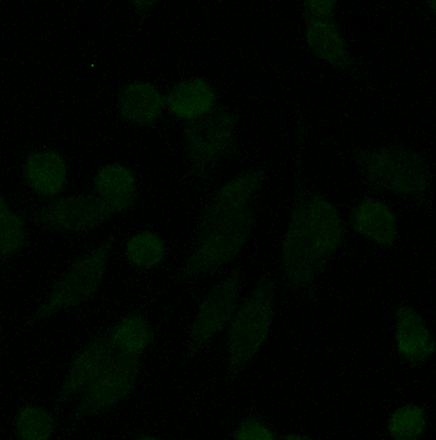

Supplement: Supplementary file 1 [file DataSheet3.ZIP › Original microscopy images1/Figure3/con ASC.tif]

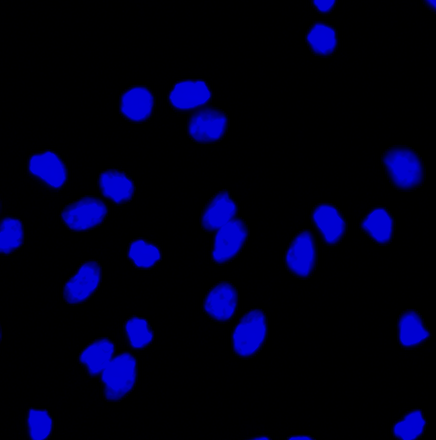

Supplement: Supplementary file 1 [file DataSheet3.ZIP › Original microscopy images1/Figure3/con DAPI.tif]

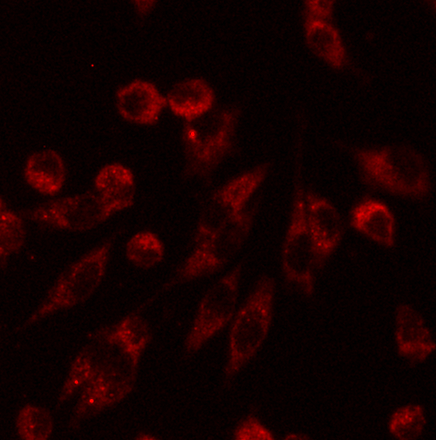

Supplement: Supplementary file 1 [file DataSheet3.ZIP › Original microscopy images1/Figure3/con IbA1.tif]

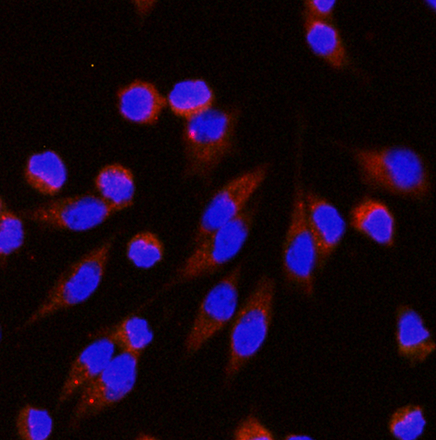

Supplement: Supplementary file 1 [file DataSheet3.ZIP › Original microscopy images1/Figure3/con merge.tif]

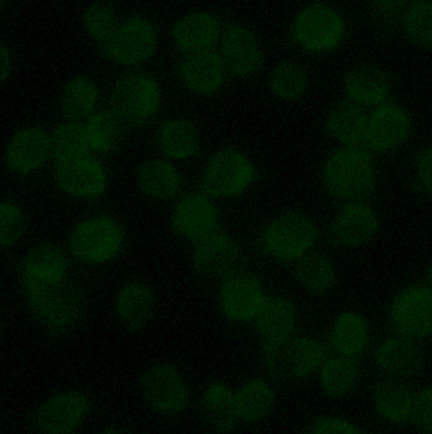

Supplement: Supplementary file 1 [file DataSheet3.ZIP › Original microscopy images1/Figure3/Model ASC.tif]

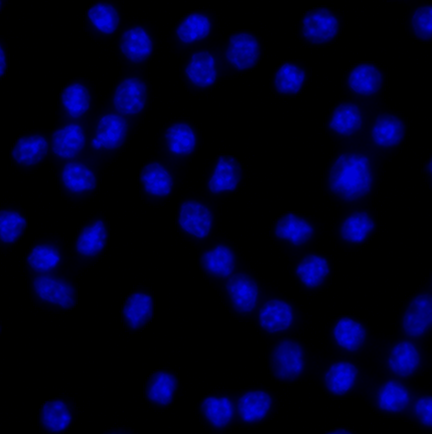

Supplement: Supplementary file 1 [file DataSheet3.ZIP › Original microscopy images1/Figure3/Model DAPI.tif]

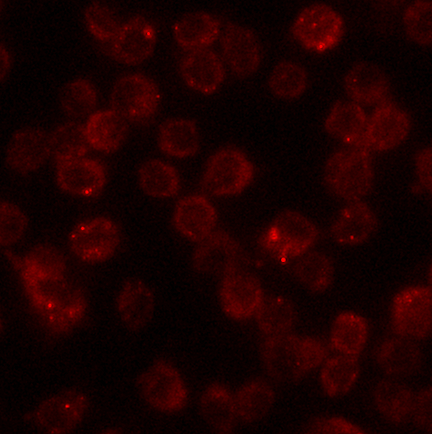

Supplement: Supplementary file 1 [file DataSheet3.ZIP › Original microscopy images1/Figure3/Model IbA1.tif]

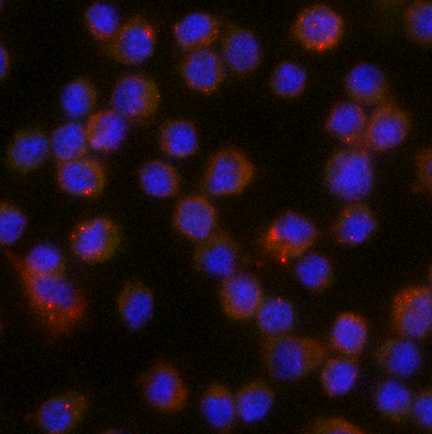

Supplement: Supplementary file 1 [file DataSheet3.ZIP › Original microscopy images1/Figure3/Model merge.tif]

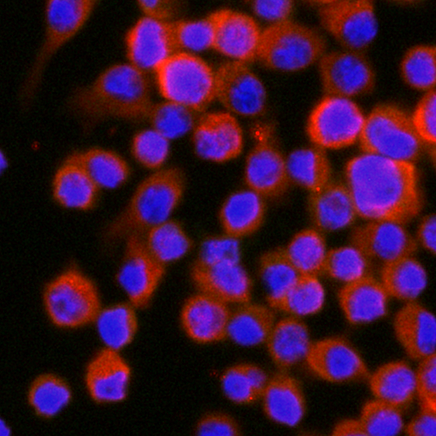

Supplement: Supplementary file 1 [file DataSheet3.ZIP › Original microscopy images1/Figure3/Model SEVO merge.tif]

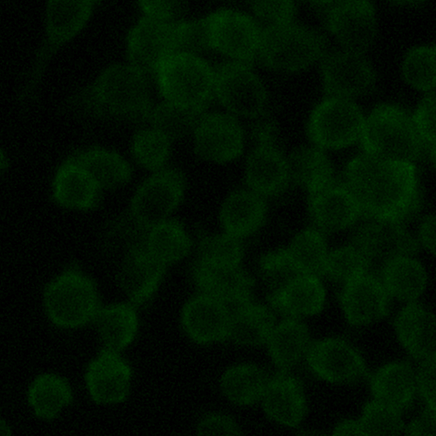

Supplement: Supplementary file 1 [file DataSheet3.ZIP › Original microscopy images1/Figure3/Model SEVO ASC.tif]

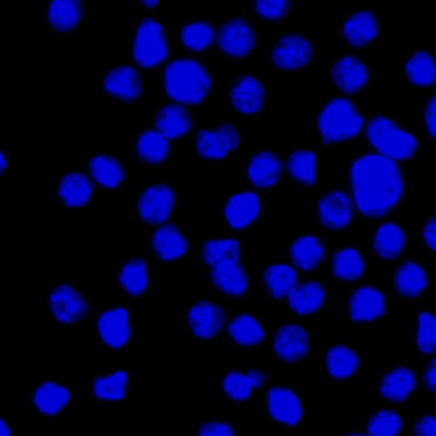

Supplement: Supplementary file 1 [file DataSheet3.ZIP › Original microscopy images1/Figure3/Model SEVO DAPI.tif]

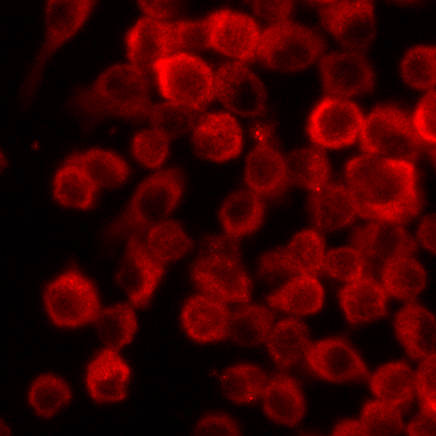

Supplement: Supplementary file 1 [file DataSheet3.ZIP › Original microscopy images1/Figure3/Model SEVO IbA1.tif]

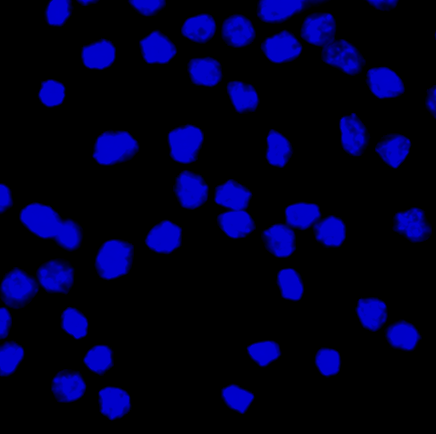

Supplement: Supplementary file 1 [file DataSheet3.ZIP › Original microscopy images1/Figure3/Model vx765 DAPI.tif]

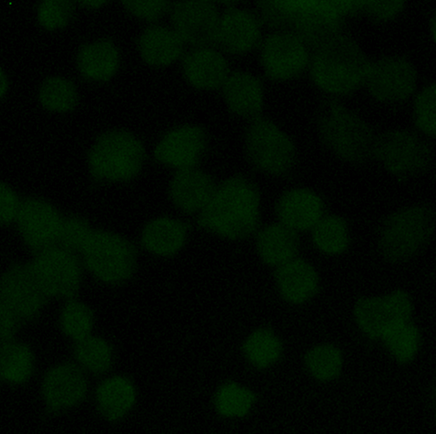

Supplement: Supplementary file 1 [file DataSheet3.ZIP › Original microscopy images1/Figure3/Model vx765 ASC.tif]

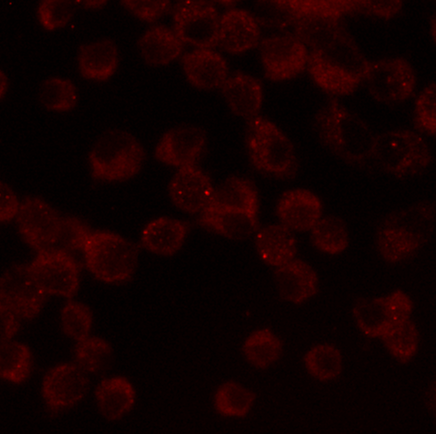

Supplement: Supplementary file 1 [file DataSheet3.ZIP › Original microscopy images1/Figure3/Model vx765 IbA1.tif]

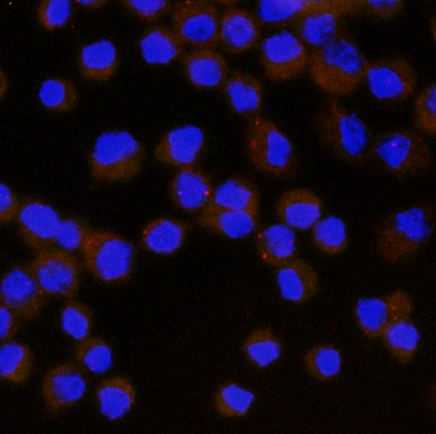

Supplement: Supplementary file 1 [file DataSheet3.ZIP › Original microscopy images1/Figure3/Model vx765 merge.tif]

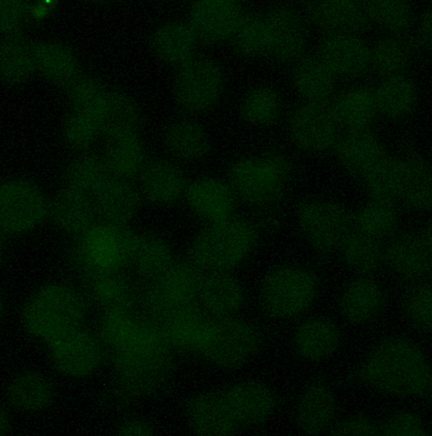

Supplement: Supplementary file 1 [file DataSheet3.ZIP › Original microscopy images1/Figure3/Model vx765 sevo ASC.tif]

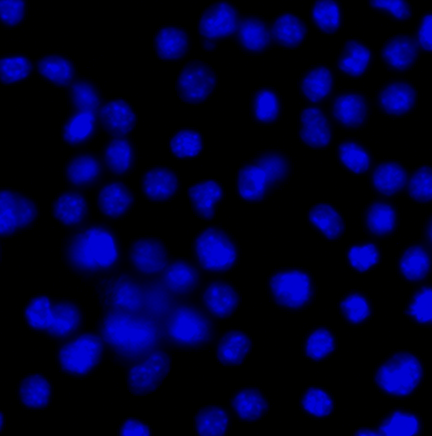

Supplement: Supplementary file 1 [file DataSheet3.ZIP › Original microscopy images1/Figure3/Model vx765 sevo DAPI.tif]

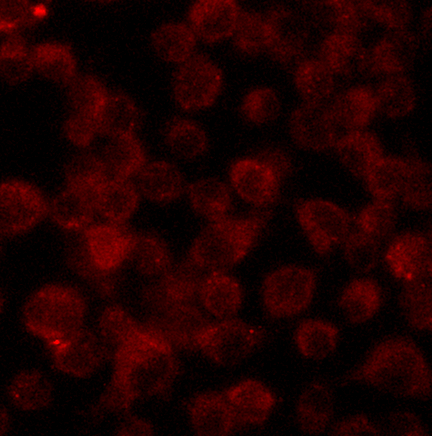

Supplement: Supplementary file 1 [file DataSheet3.ZIP › Original microscopy images1/Figure3/Model vx765 sevo IbA1.tif]

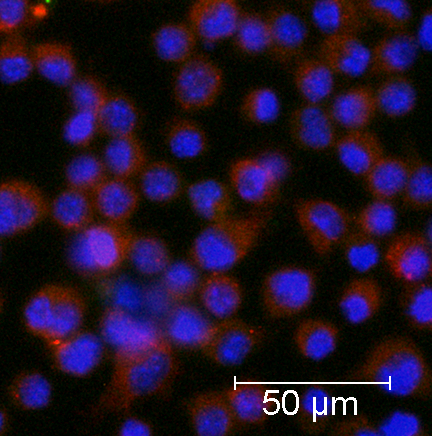

Supplement: Supplementary file 1 [file DataSheet3.ZIP › Original microscopy images1/Figure3/Model vx765 sevo merge.tif]

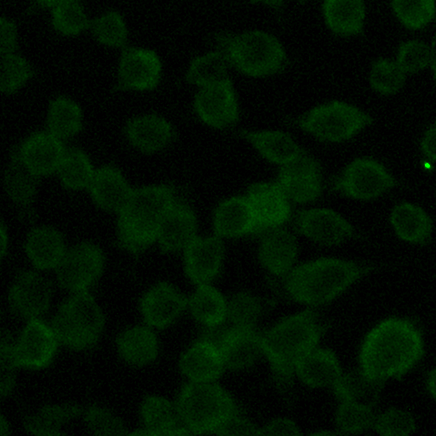

Supplement: Supplementary file 1 [file DataSheet3.ZIP › Original microscopy images1/Figure3/sevo ASC.tif]

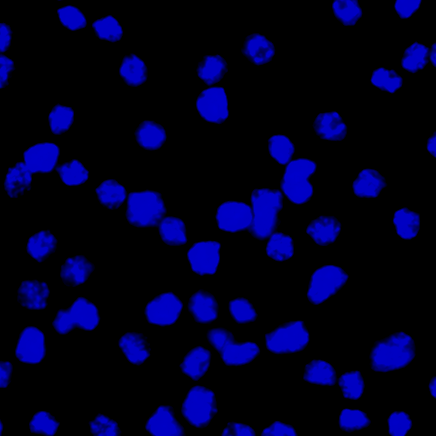

Supplement: Supplementary file 1 [file DataSheet3.ZIP › Original microscopy images1/Figure3/sevo DAPI.tif]

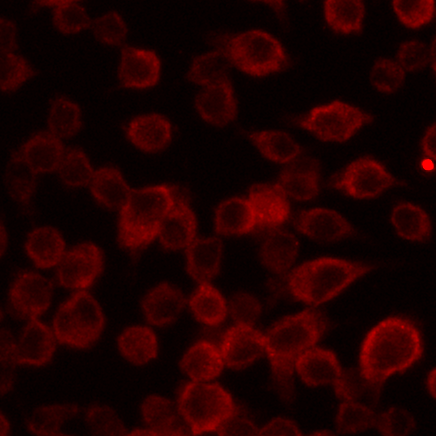

Supplement: Supplementary file 1 [file DataSheet3.ZIP › Original microscopy images1/Figure3/sevo IbA1.tif]

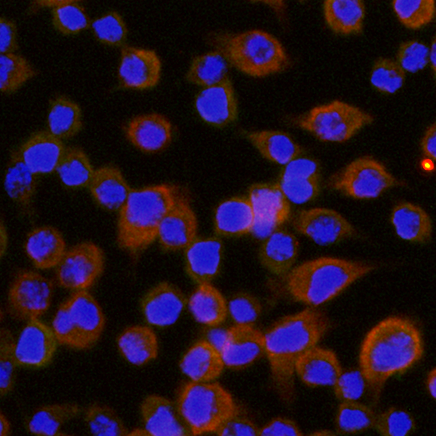

Supplement: Supplementary file 1 [file DataSheet3.ZIP › Original microscopy images1/Figure3/sevo merge.tif]

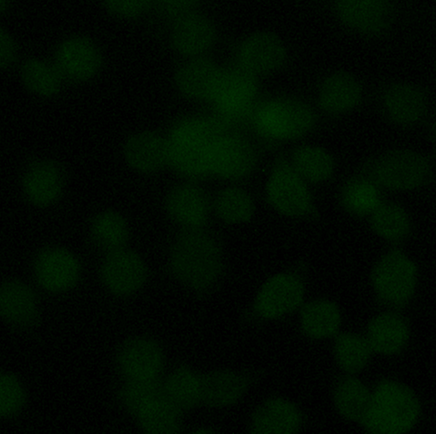

Supplement: Supplementary file 1 [file DataSheet3.ZIP › Original microscopy images1/Figure3/vx765 ASC.tif]

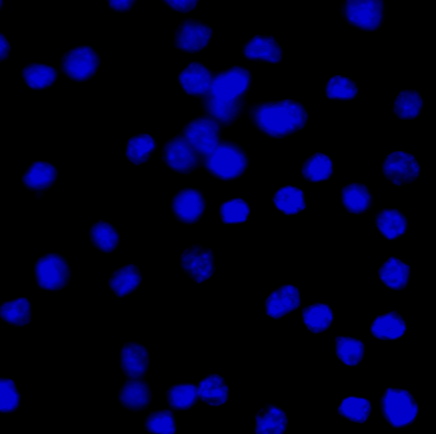

Supplement: Supplementary file 1 [file DataSheet3.ZIP › Original microscopy images1/Figure3/vx765 DAPI.tif]

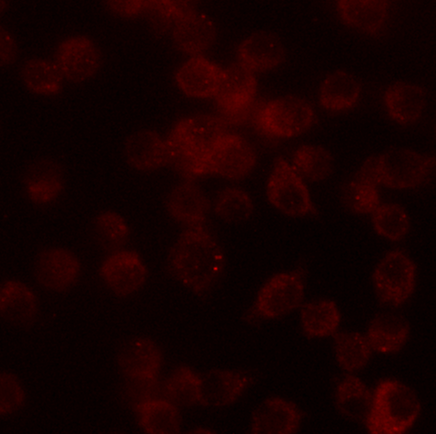

Supplement: Supplementary file 1 [file DataSheet3.ZIP › Original microscopy images1/Figure3/vx765 IbA1.tif]

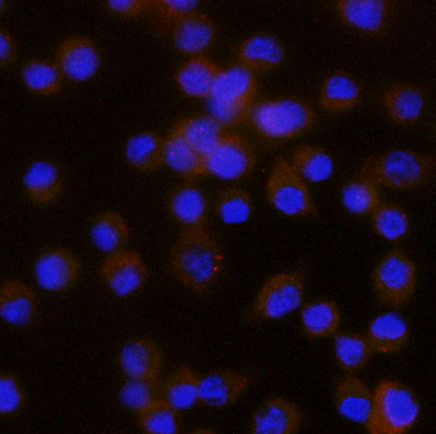

Supplement: Supplementary file 1 [file DataSheet3.ZIP › Original microscopy images1/Figure3/vx765 merge.tif]

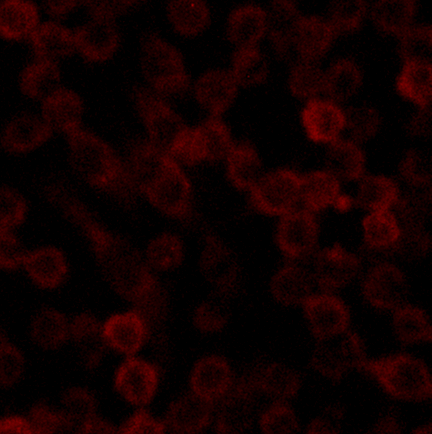

Supplement: Supplementary file 1 [file DataSheet3.ZIP › Original microscopy images1/Figure3/vx765 sevo IbA1.tif]

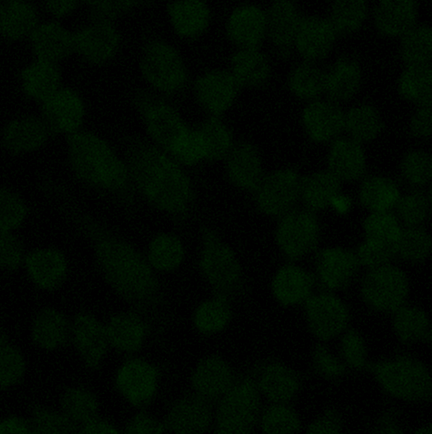

Supplement: Supplementary file 1 [file DataSheet3.ZIP › Original microscopy images1/Figure3/vx765 sevo ASC.tif]

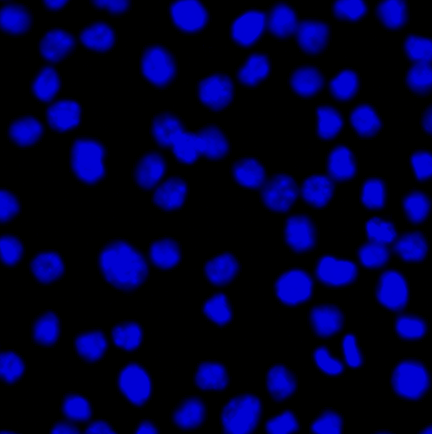

Supplement: Supplementary file 1 [file DataSheet3.ZIP › Original microscopy images1/Figure3/vx765 sevo DAPI.tif]

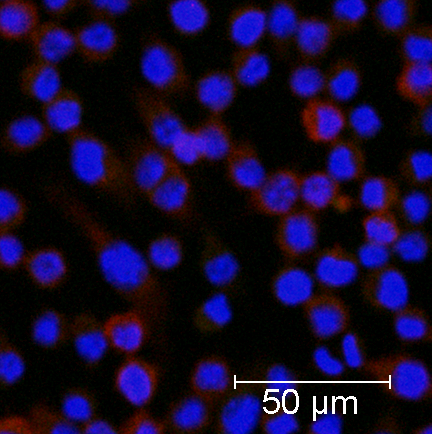

Supplement: Supplementary file 1 [file DataSheet3.ZIP › Original microscopy images1/Figure3/vx765 sevo merge.tif]

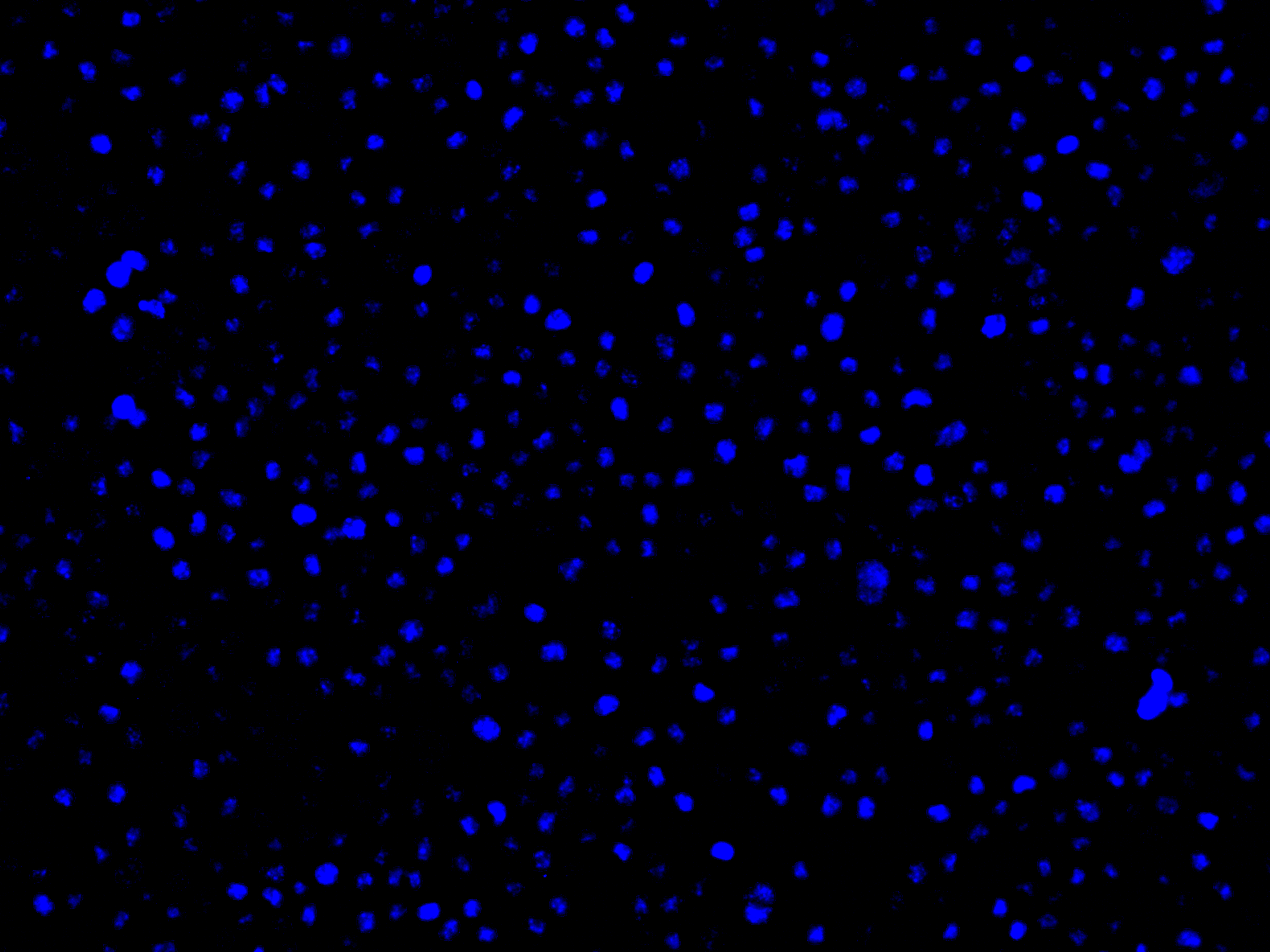

Supplement: Supplementary file 2 [file DataSheet4.ZIP › Original microscopy images2/Figure5/bv2 con DAPI.tif]

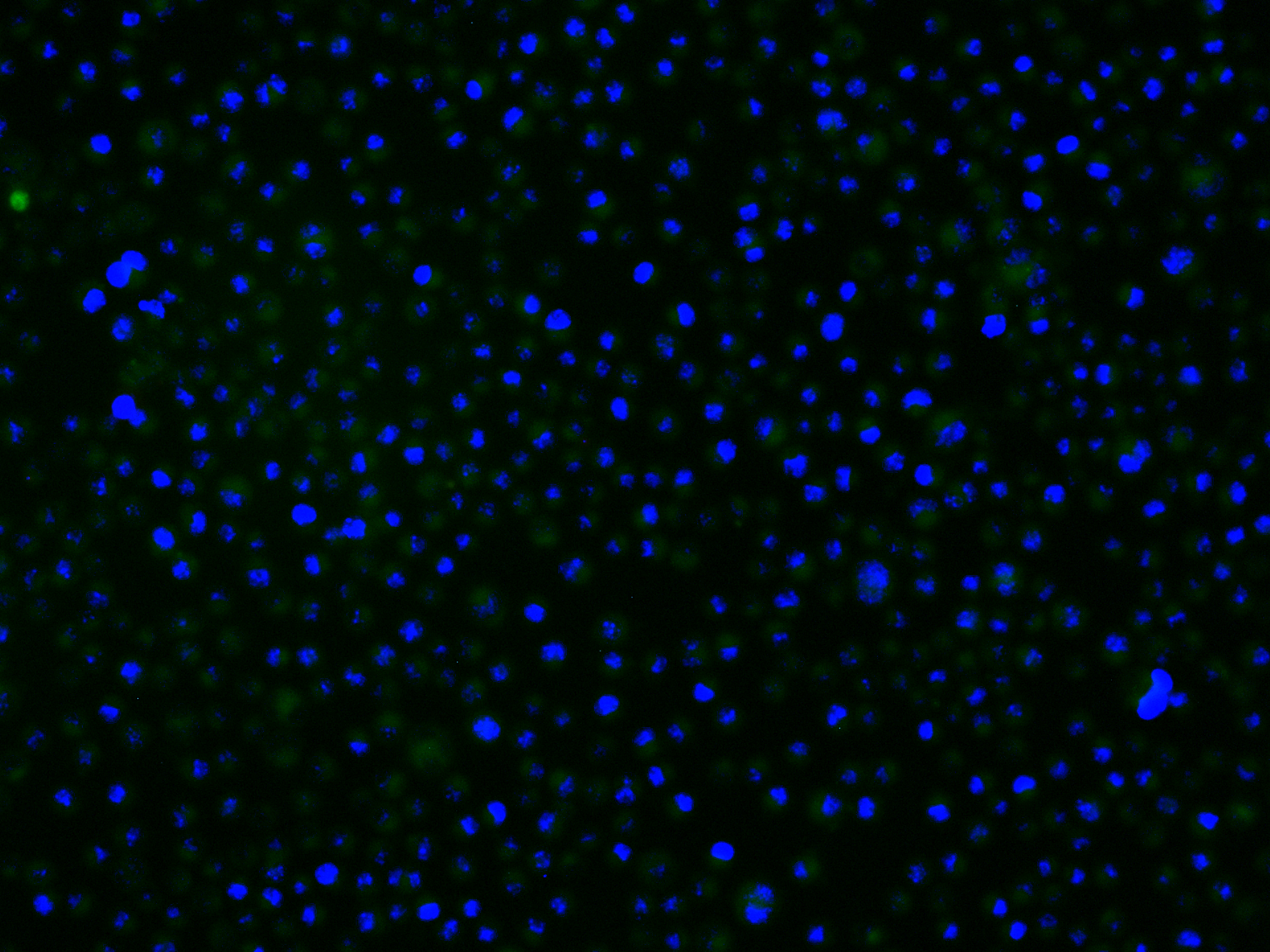

Supplement: Supplementary file 2 [file DataSheet4.ZIP › Original microscopy images2/Figure5/bv2 con merge.tif]

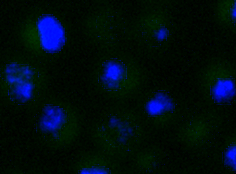

Supplement: Supplementary file 2 [file DataSheet4.ZIP › Original microscopy images2/Figure5/bv2 con mergify.tif]

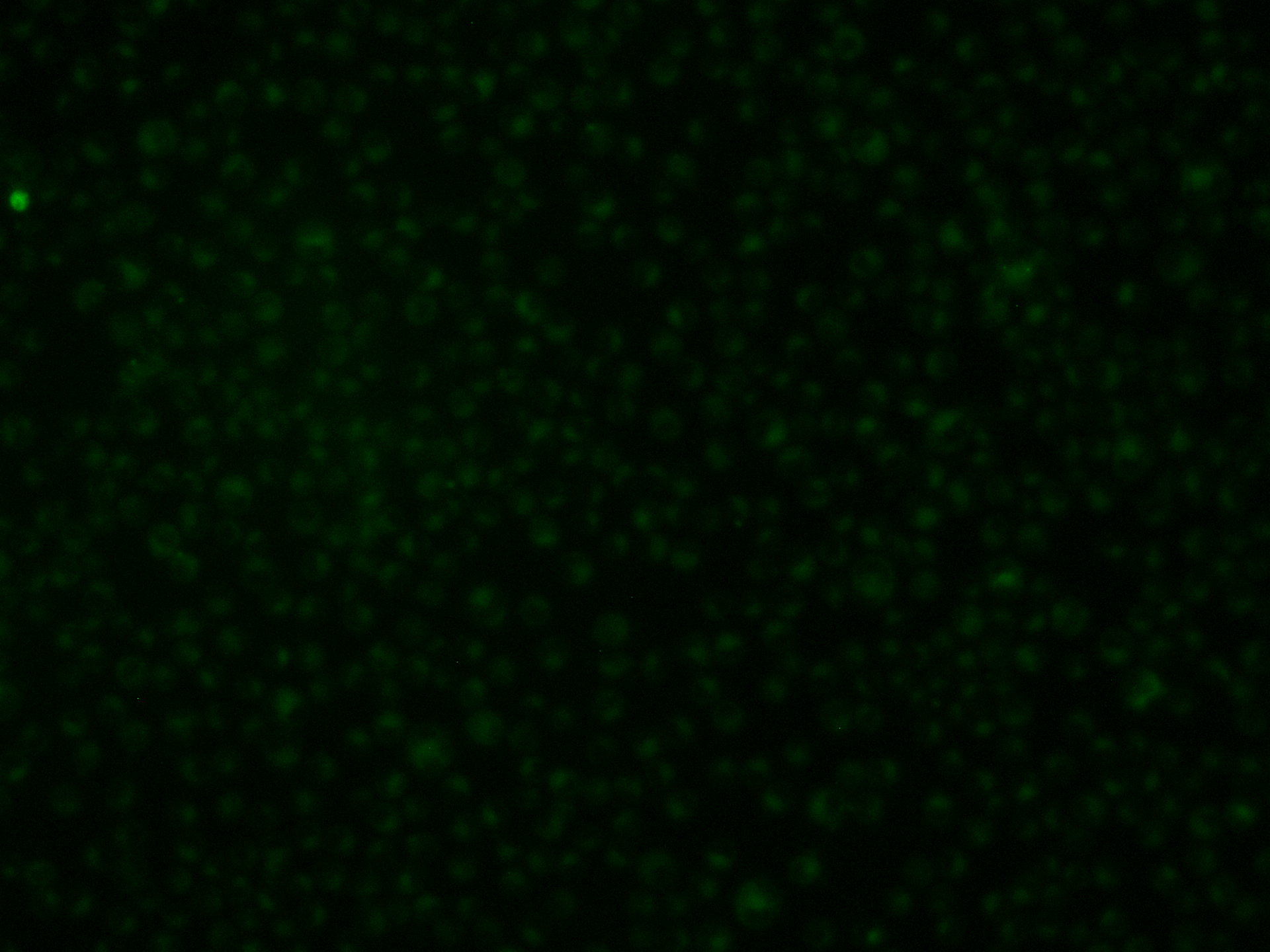

Supplement: Supplementary file 2 [file DataSheet4.ZIP › Original microscopy images2/Figure5/bv2 con P10-casp1.tif]

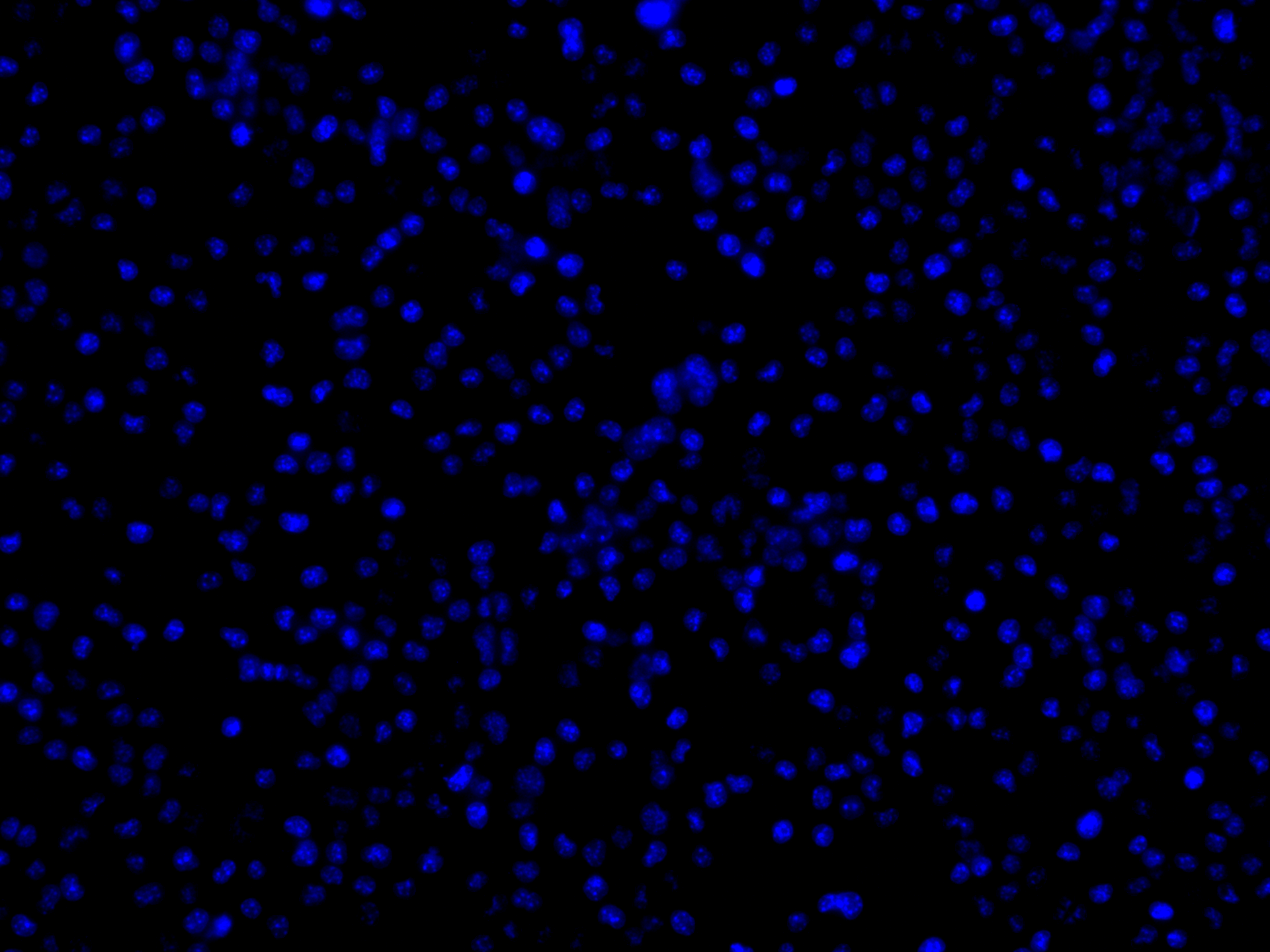

Supplement: Supplementary file 2 [file DataSheet4.ZIP › Original microscopy images2/Figure5/bv2 model DAPI.tif]

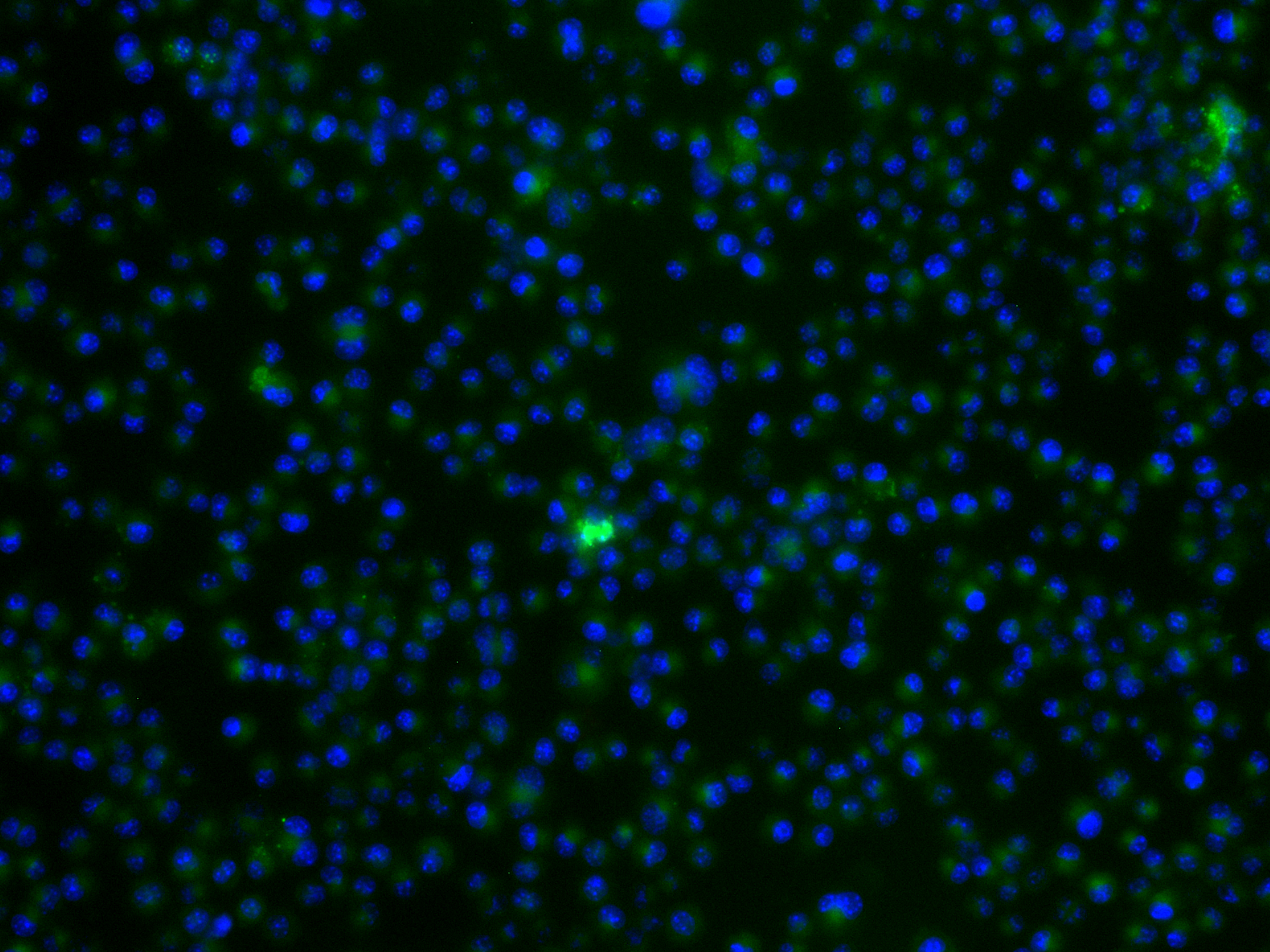

Supplement: Supplementary file 2 [file DataSheet4.ZIP › Original microscopy images2/Figure5/bv2 model merge.tif]

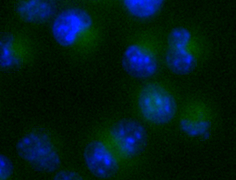

Supplement: Supplementary file 2 [file DataSheet4.ZIP › Original microscopy images2/Figure5/bv2 model mergify.tif]

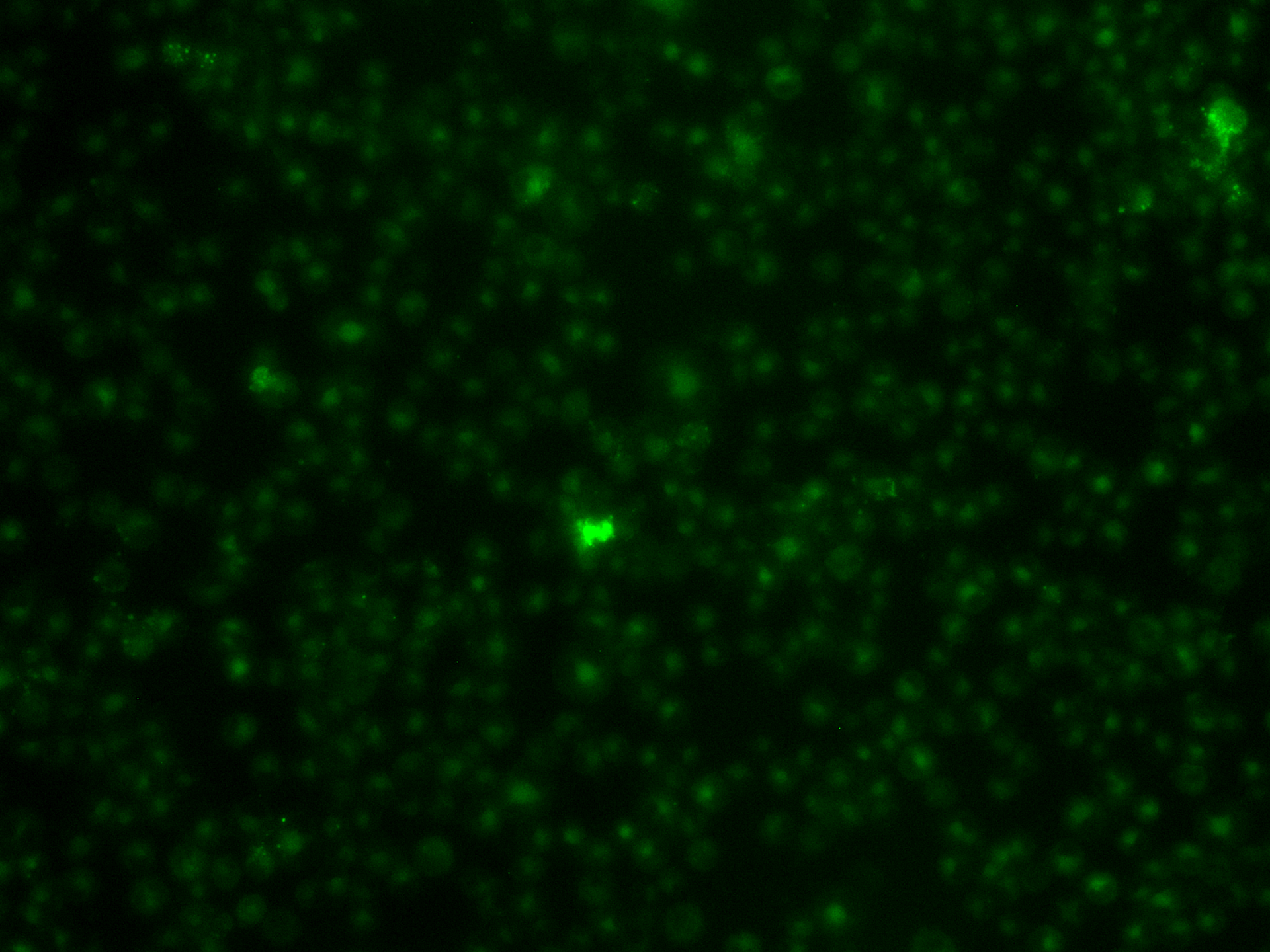

Supplement: Supplementary file 2 [file DataSheet4.ZIP › Original microscopy images2/Figure5/bv2 model P10-casp1.tif]

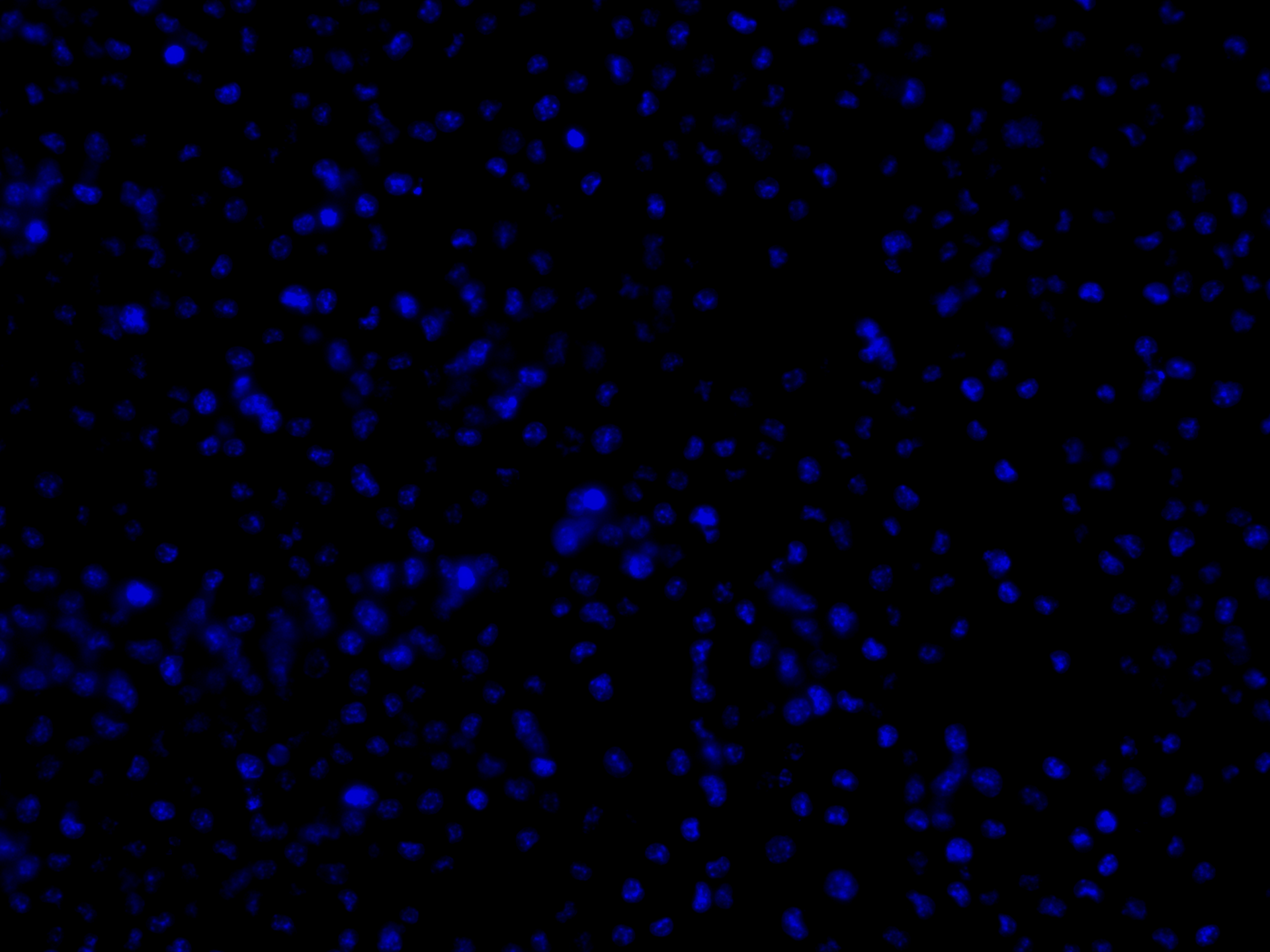

Supplement: Supplementary file 2 [file DataSheet4.ZIP › Original microscopy images2/Figure5/bv2 model sevo DAPI.tif]

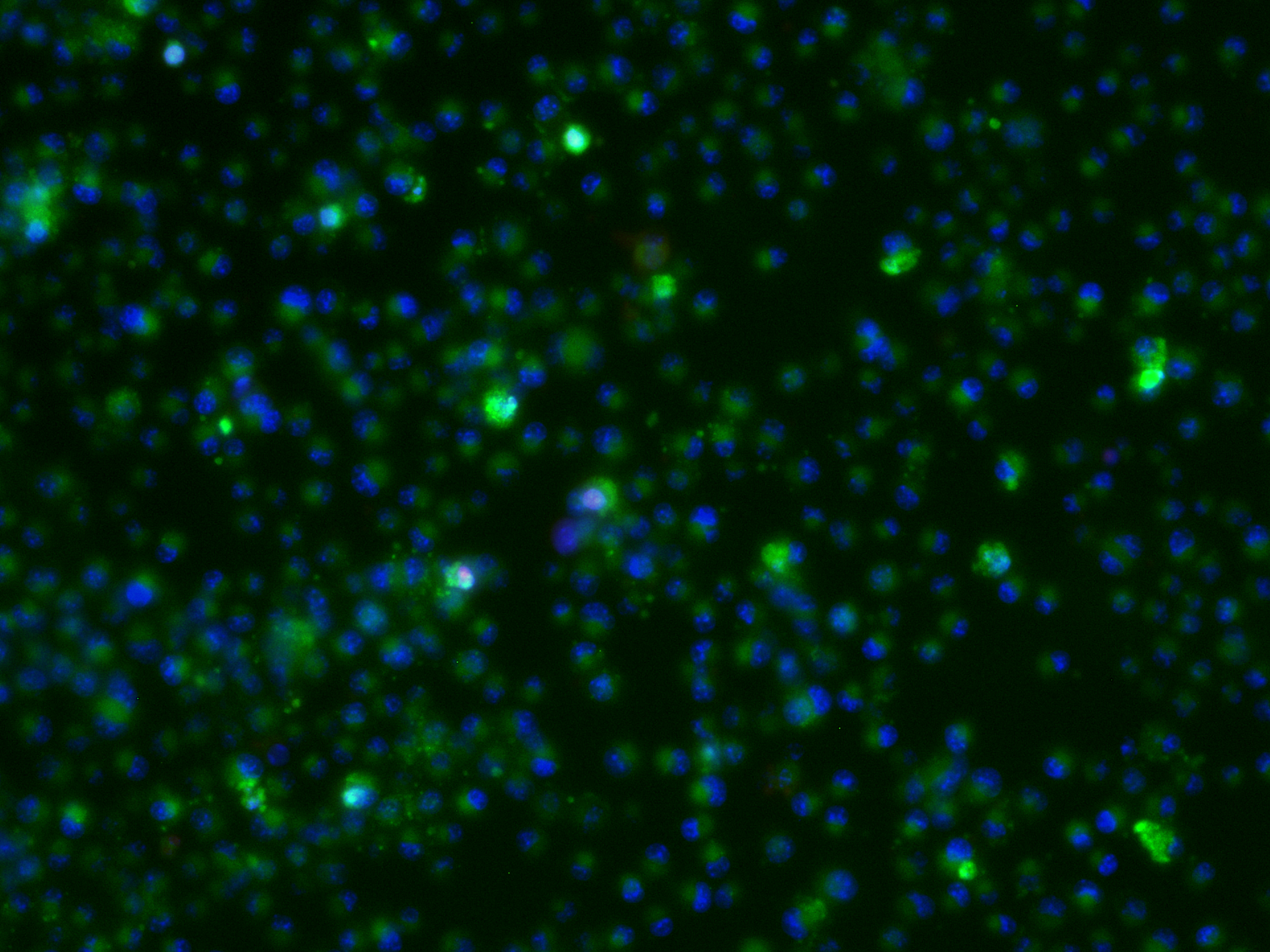

Supplement: Supplementary file 2 [file DataSheet4.ZIP › Original microscopy images2/Figure5/bv2 model sevo merge.tif]

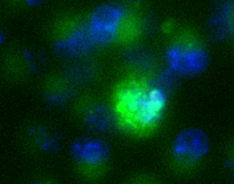

Supplement: Supplementary file 2 [file DataSheet4.ZIP › Original microscopy images2/Figure5/bv2 model sevo mergify.tif]

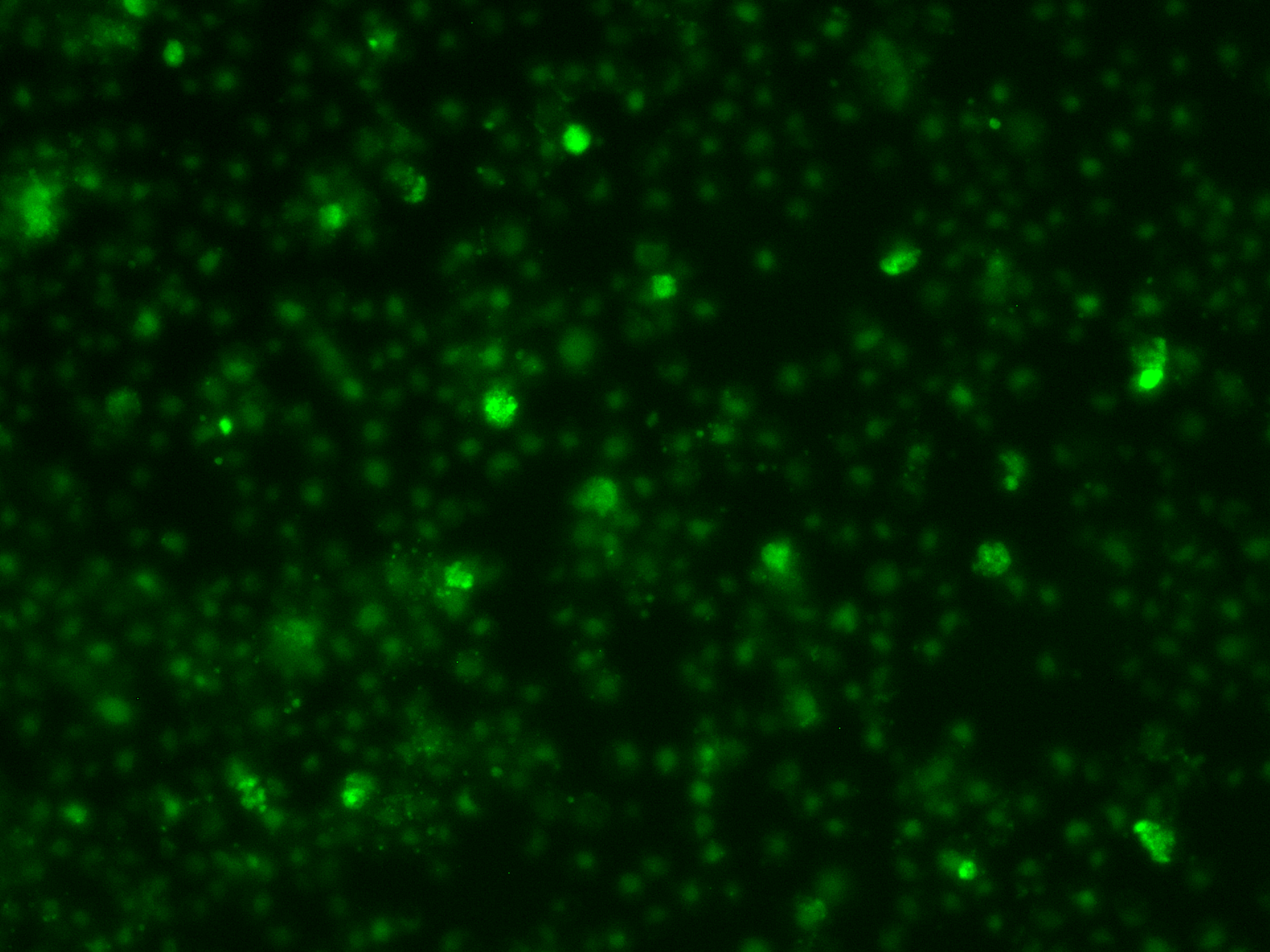

Supplement: Supplementary file 2 [file DataSheet4.ZIP › Original microscopy images2/Figure5/bv2 model sevo P10-casp1.tif]

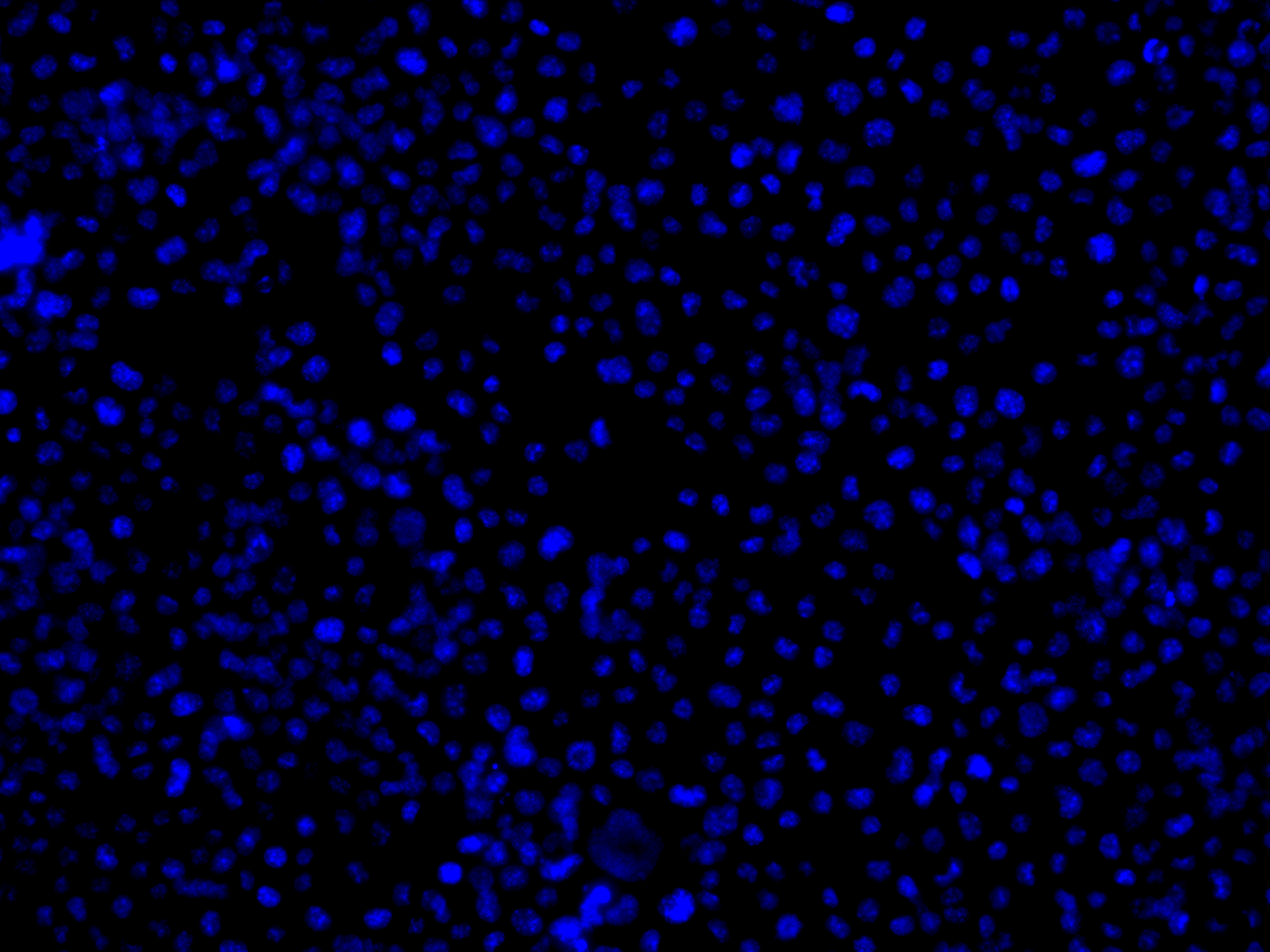

Supplement: Supplementary file 2 [file DataSheet4.ZIP › Original microscopy images2/Figure5/NLRP3 con DAPI.tif]

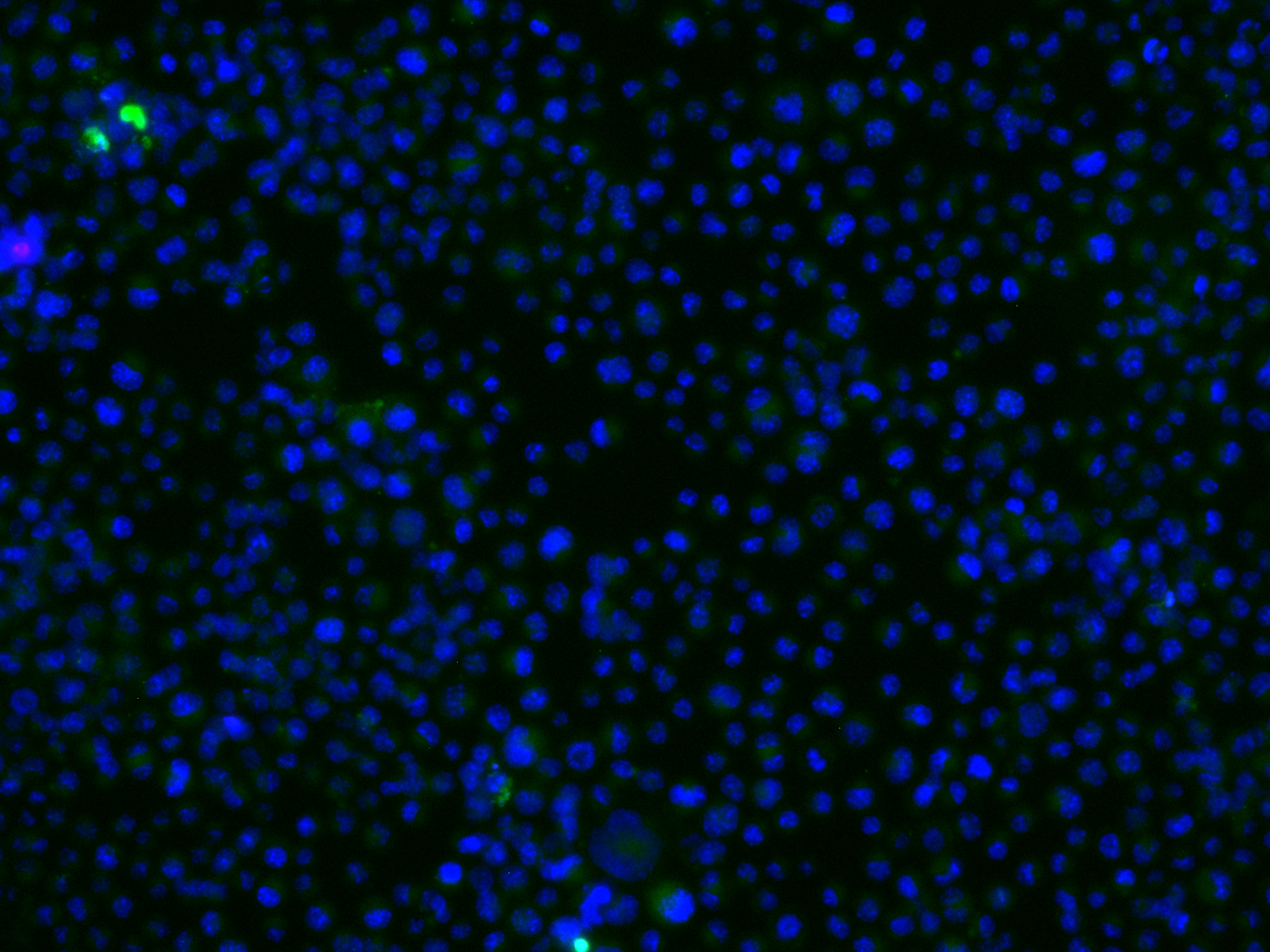

Supplement: Supplementary file 2 [file DataSheet4.ZIP › Original microscopy images2/Figure5/NLRP3 con merge.tif]

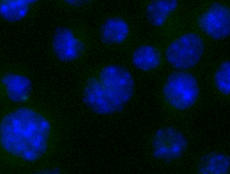

Supplement: Supplementary file 2 [file DataSheet4.ZIP › Original microscopy images2/Figure5/NLRP3 con mergify.tif]

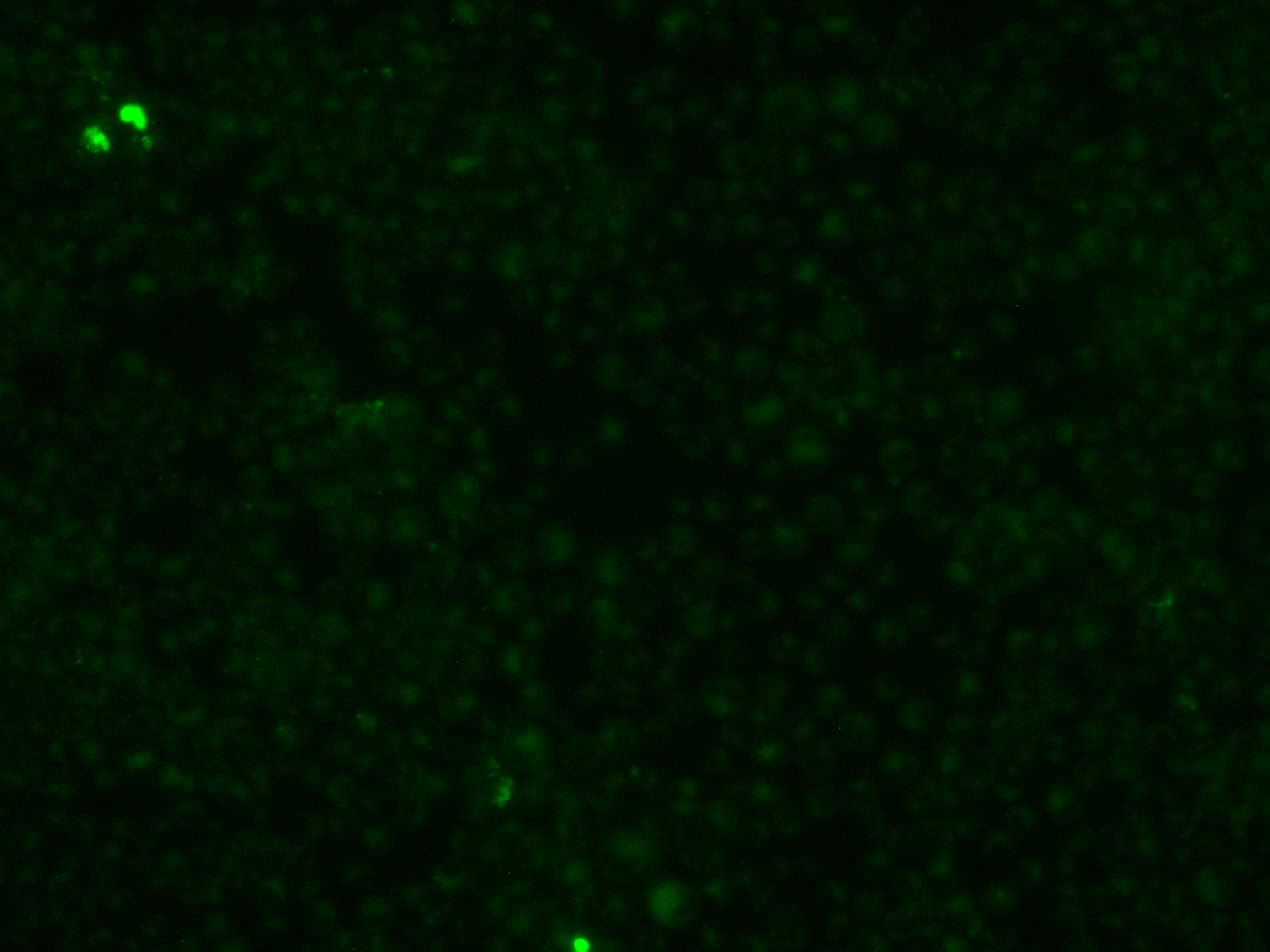

Supplement: Supplementary file 2 [file DataSheet4.ZIP › Original microscopy images2/Figure5/NLRP3 con P10-casp1.tif]

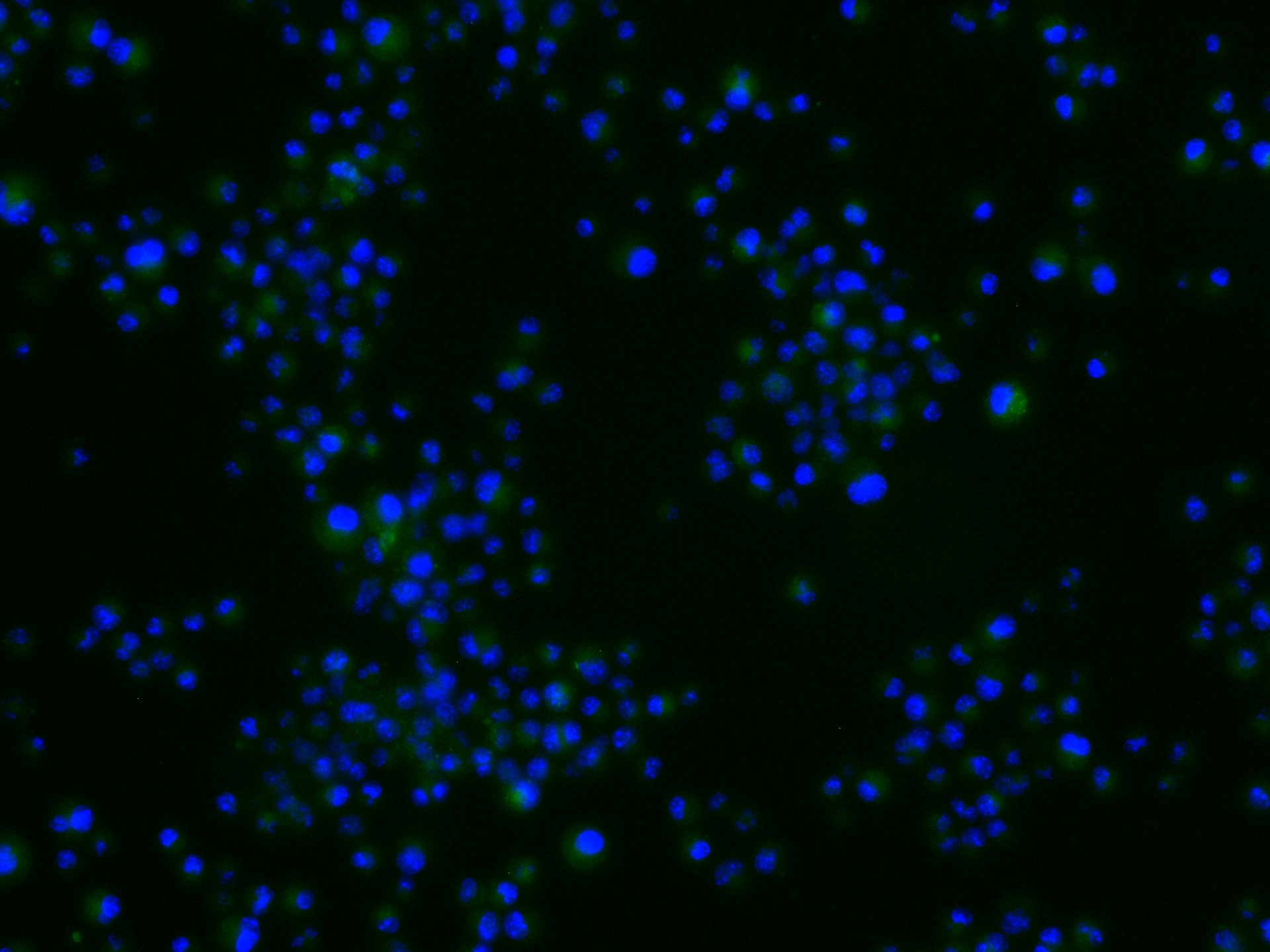

Supplement: Supplementary file 2 [file DataSheet4.ZIP › Original microscopy images2/Figure5/NLRP3 model merge.tif]

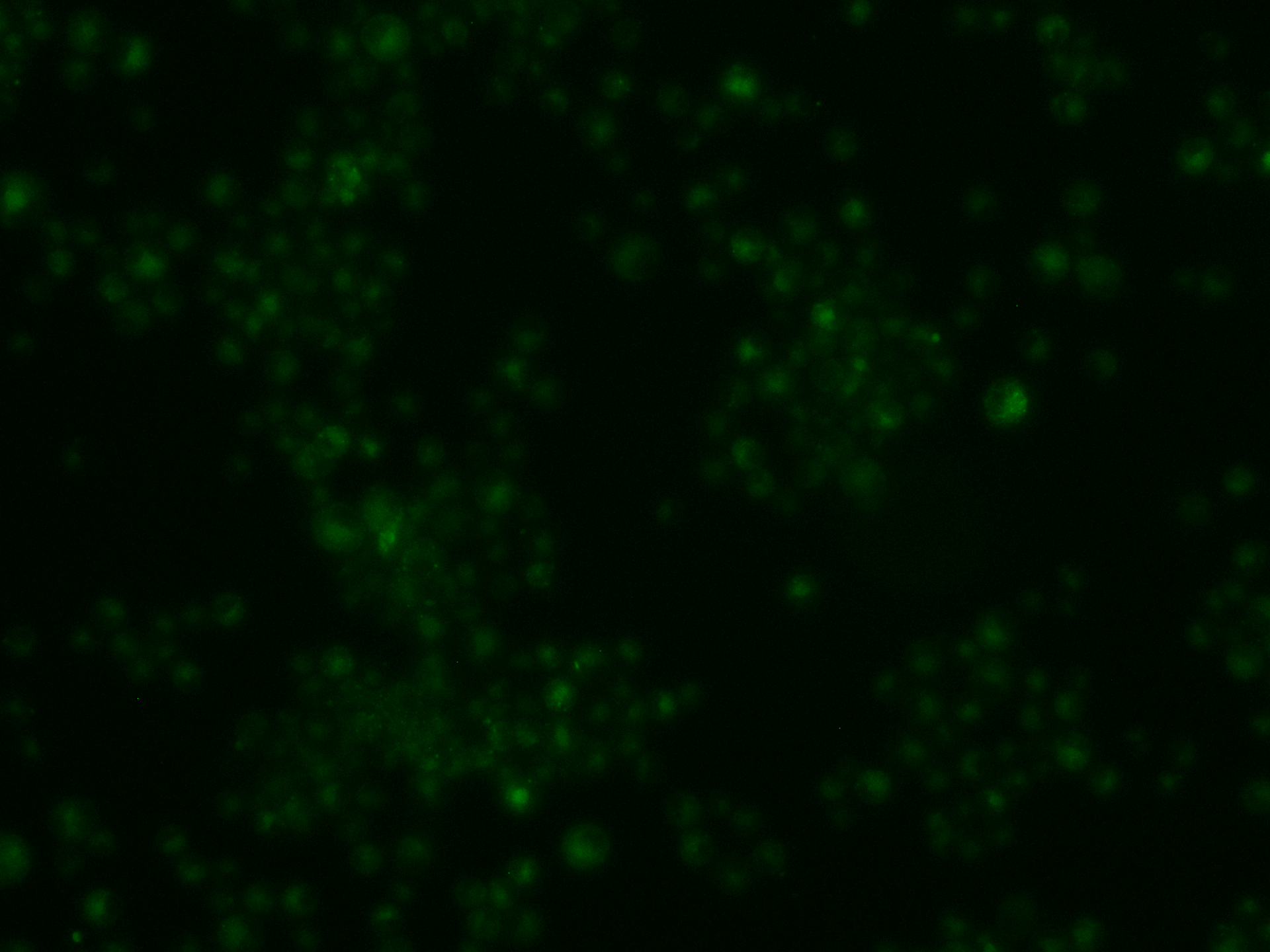

Supplement: Supplementary file 2 [file DataSheet4.ZIP › Original microscopy images2/Figure5/NLRP3 model p10-casp1.tif]

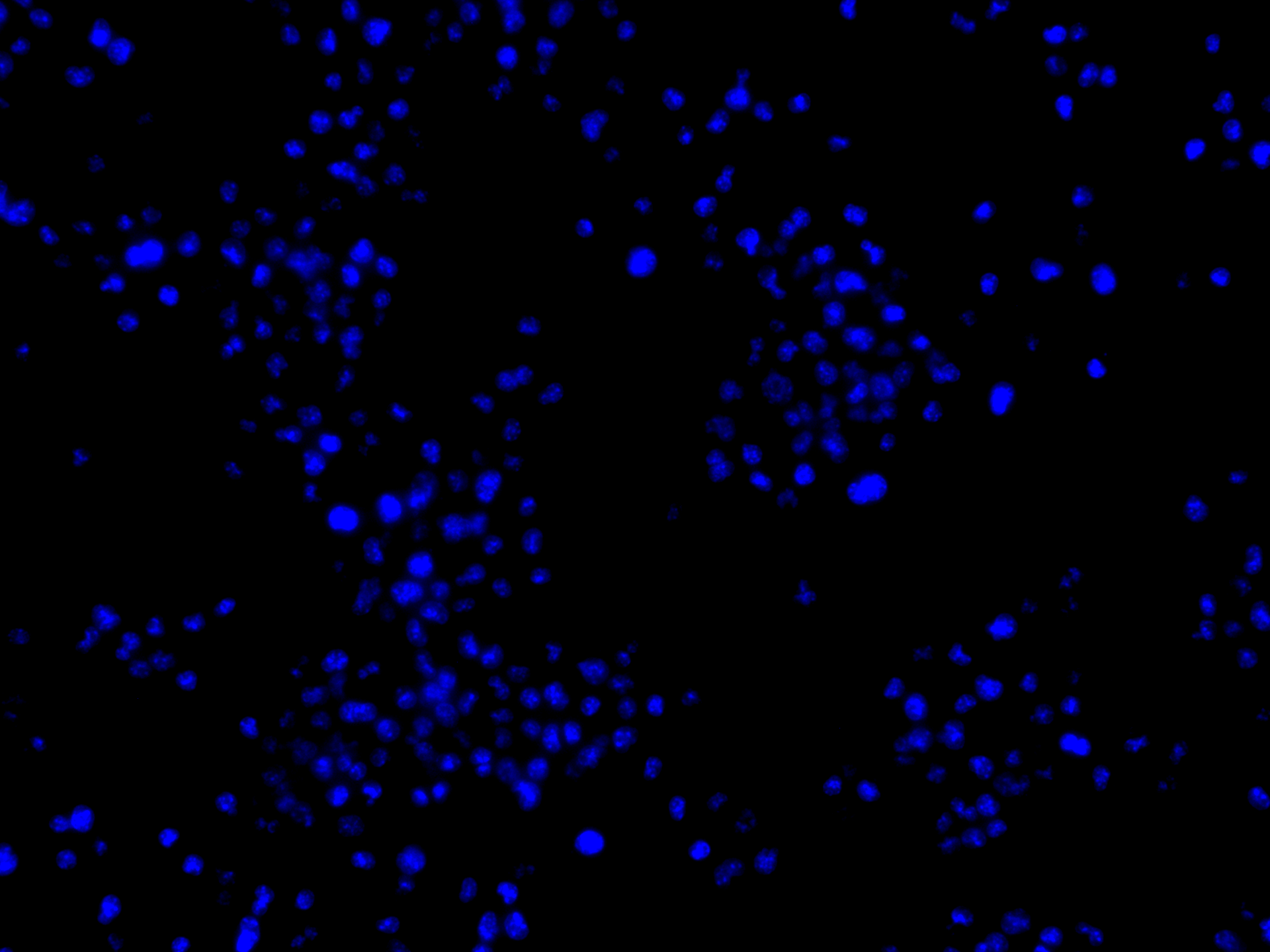

Supplement: Supplementary file 2 [file DataSheet4.ZIP › Original microscopy images2/Figure5/NLRP3 model DAPI.tif]

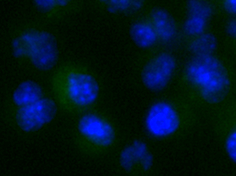

Supplement: Supplementary file 2 [file DataSheet4.ZIP › Original microscopy images2/Figure5/NLRP3 model mergify.tif]

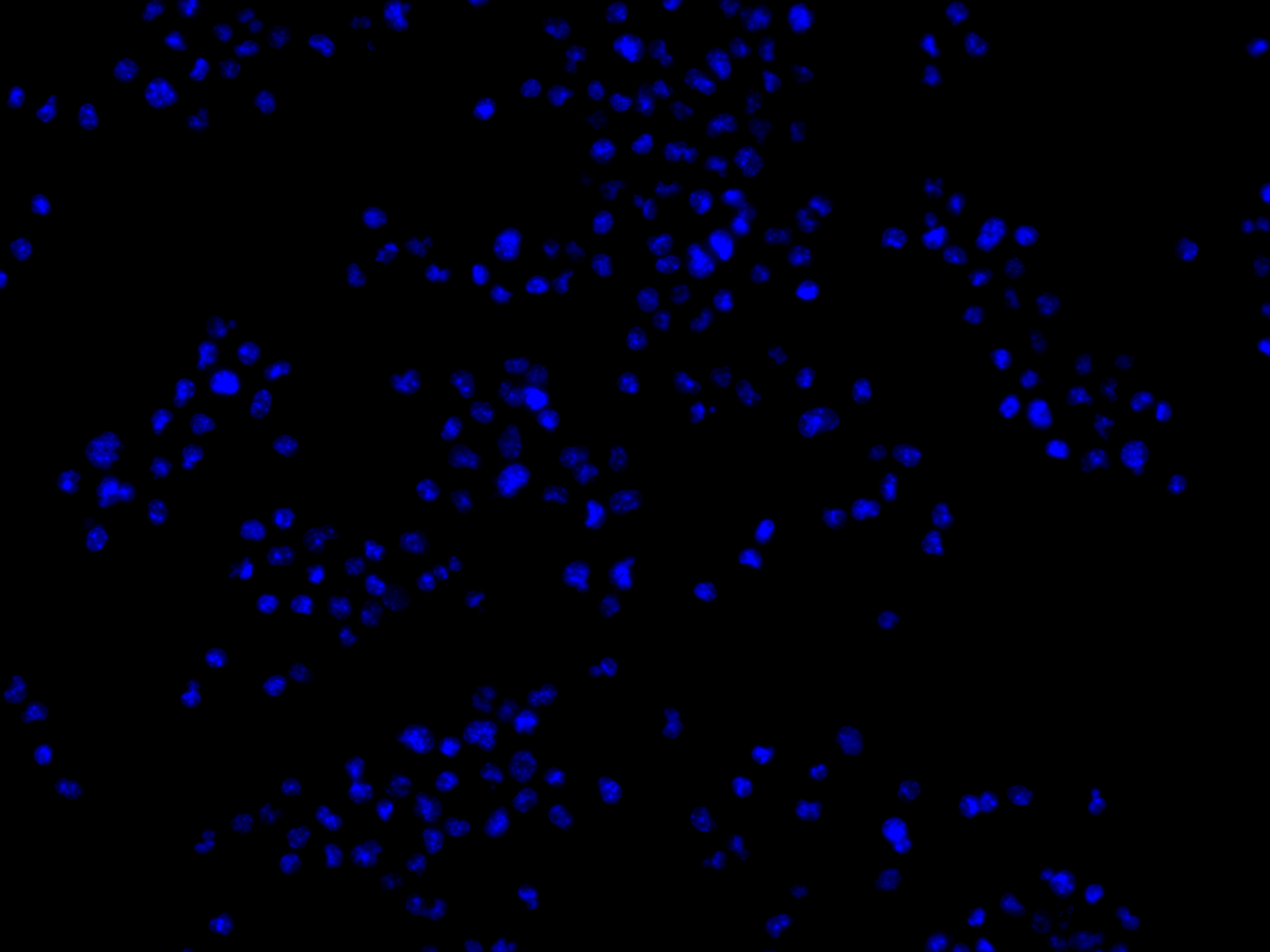

Supplement: Supplementary file 2 [file DataSheet4.ZIP › Original microscopy images2/Figure5/NLRP3 model sevo DAPI.tif]

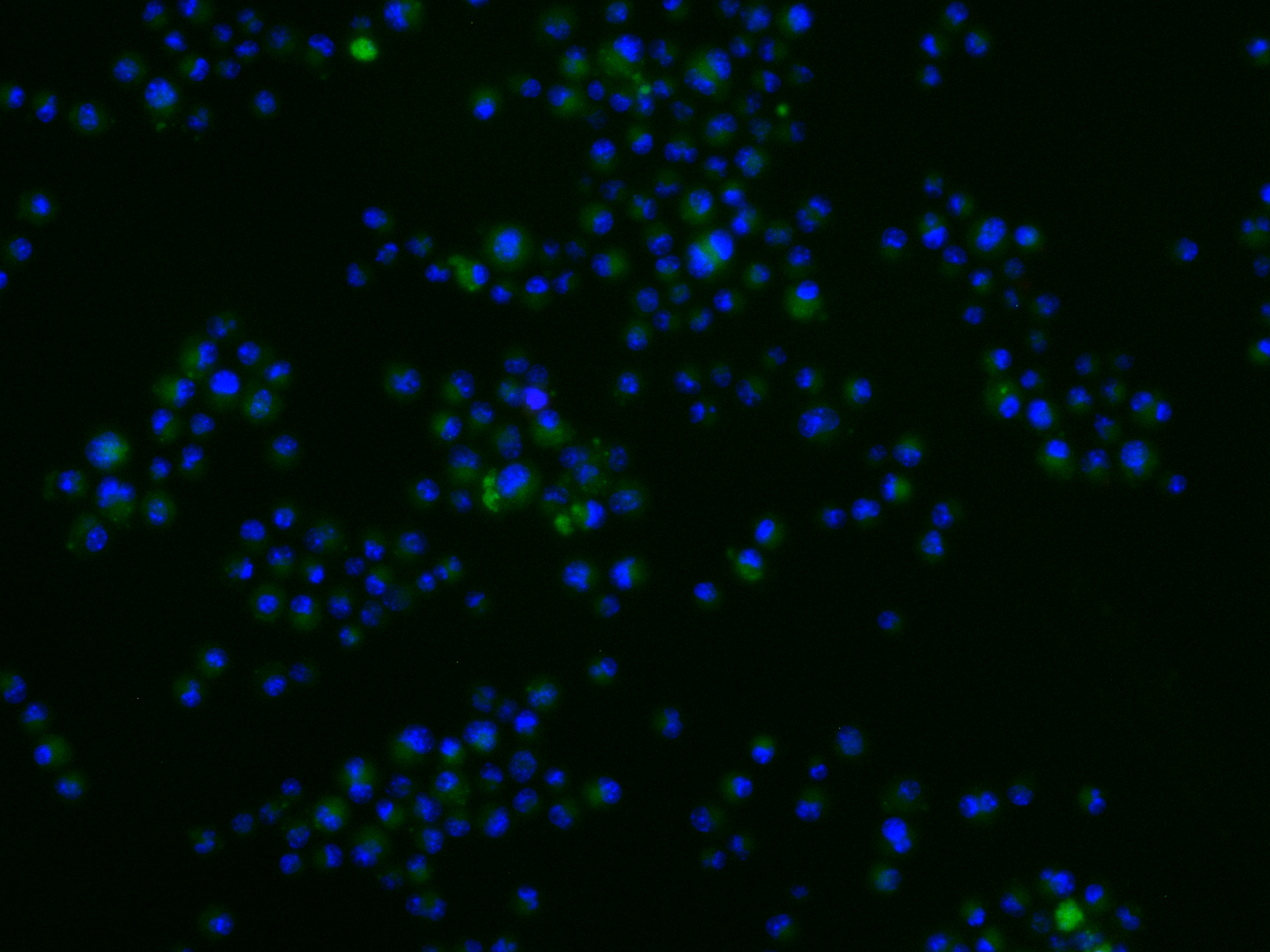

Supplement: Supplementary file 2 [file DataSheet4.ZIP › Original microscopy images2/Figure5/NLRP3 model sevo merge.tif]

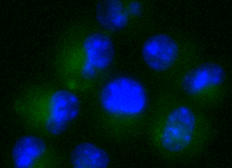

Supplement: Supplementary file 2 [file DataSheet4.ZIP › Original microscopy images2/Figure5/NLRP3 model sevo mergify.tif]

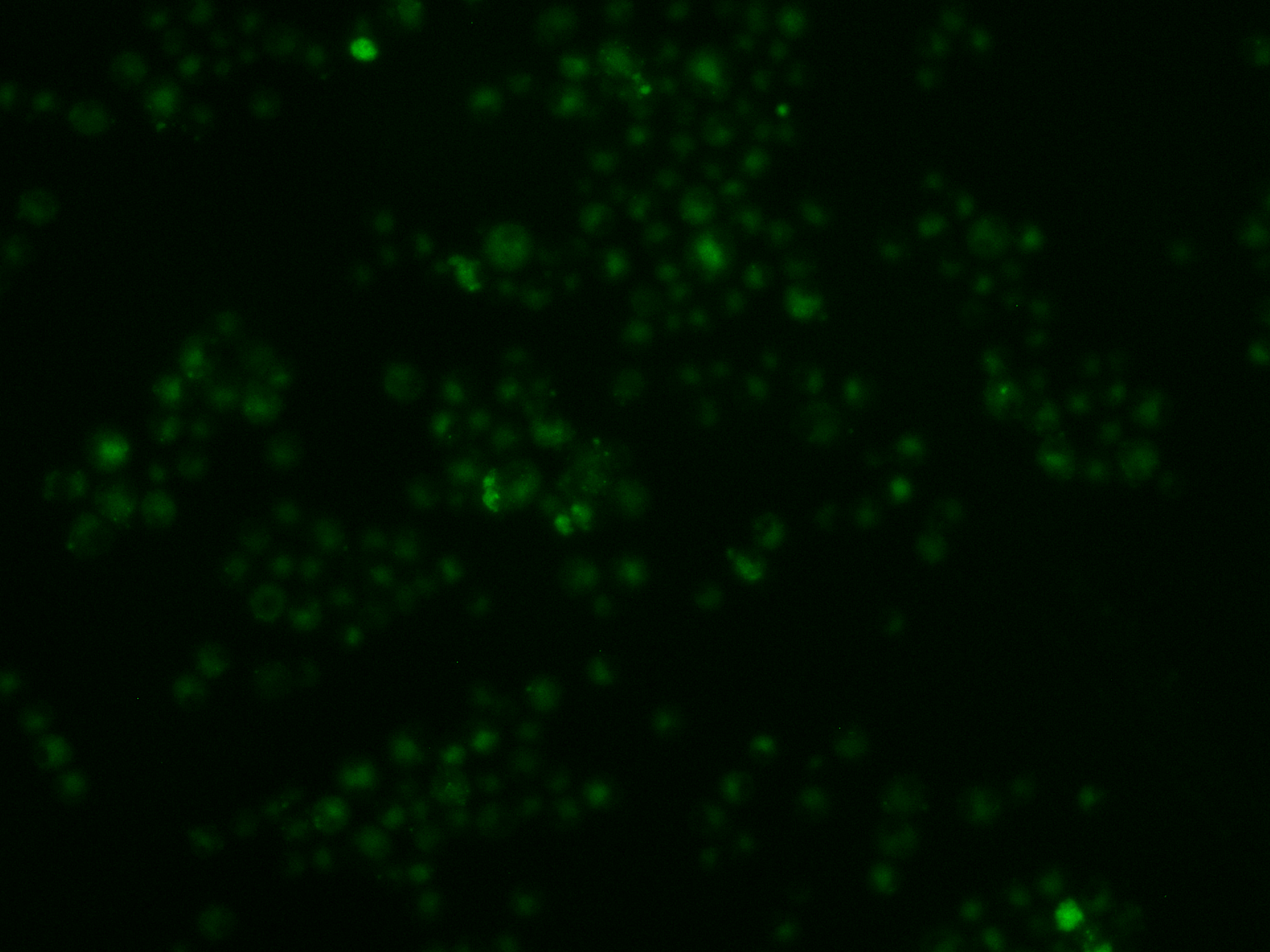

Supplement: Supplementary file 2 [file DataSheet4.ZIP › Original microscopy images2/Figure5/NLRP3 model sevo p10-casp1.tif]

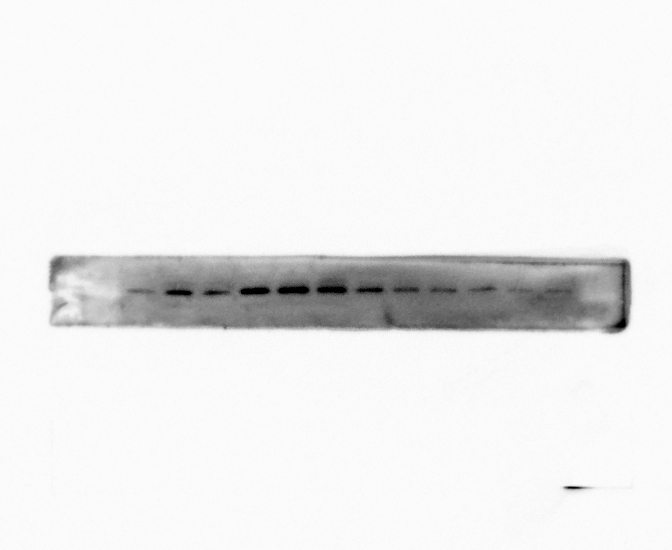

Supplement: Supplementary file 4 [file DataSheet2.ZIP › Original images and data in supplementary figures/Supplementary figure2/S2-AB.tif]

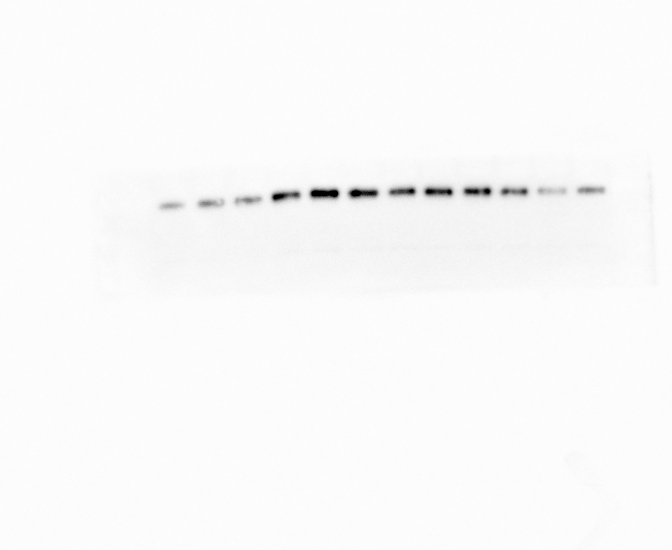

Supplement: Supplementary file 4 [file DataSheet2.ZIP › Original images and data in supplementary figures/Supplementary figure2/S2-C-CAPS1.tif]
