# Supplementary figures and images for: Sevoflurane Aggravates the Progress of Alzheimer’s Disease Through NLRP3/Caspase-1/Gasdermin D Pathway (part 2 of 2)
Source: Front Cell Dev Biol. 2022 Jan 19;9:801422. doi: 10.3389/fcell.2021.801422 (PMC8807556; doi:10.3389/fcell.2021.801422)

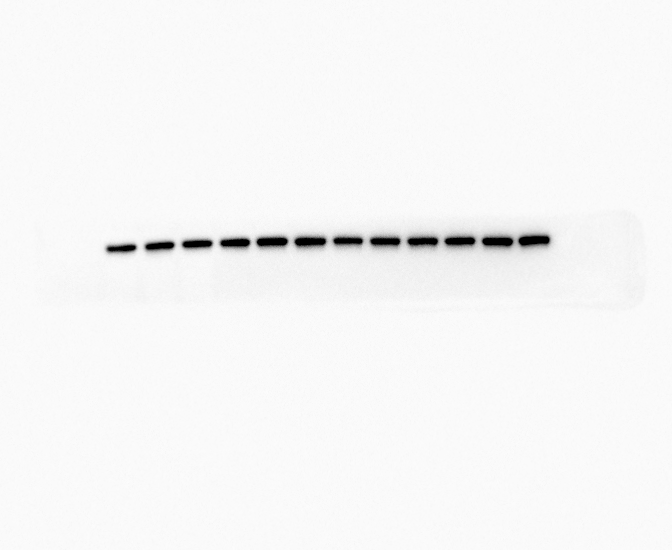

Supplement: Supplementary file 4 [file DataSheet2.ZIP › Original images and data in supplementary figures/Supplementary figure2/S2-GAPDH.tif]

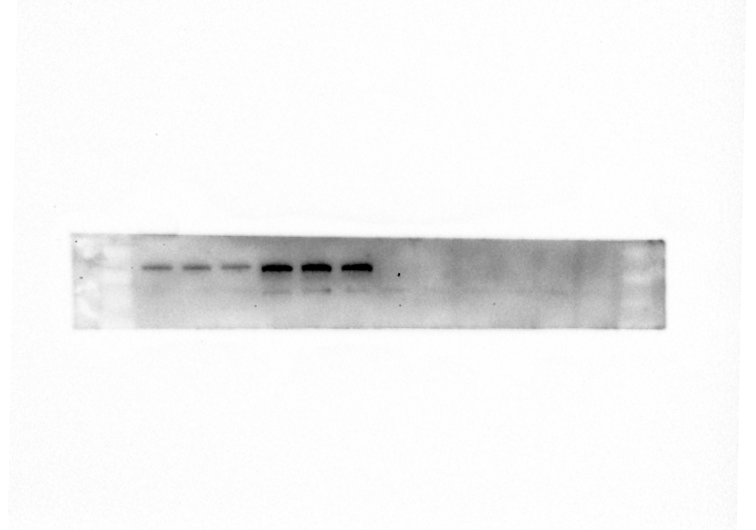

Supplement: Supplementary file 4 [file DataSheet2.ZIP › Original images and data in supplementary figures/Supplementary figure2/S2-NLRP3.tif]

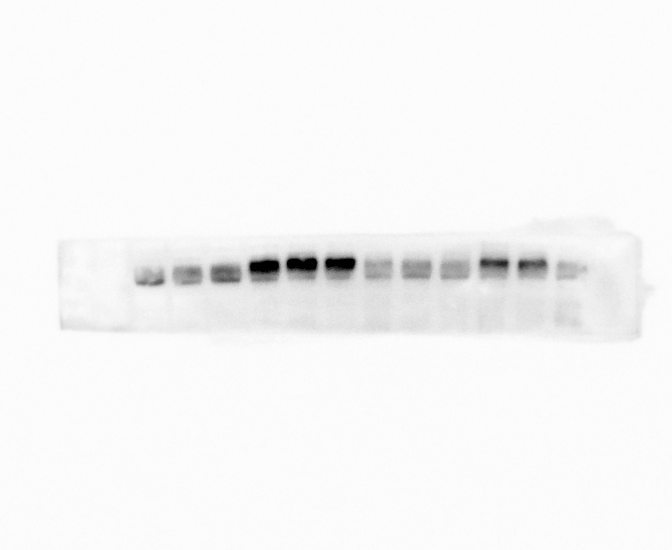

Supplement: Supplementary file 4 [file DataSheet2.ZIP › Original images and data in supplementary figures/Supplementary figure2/S2-P-TAU.tif]

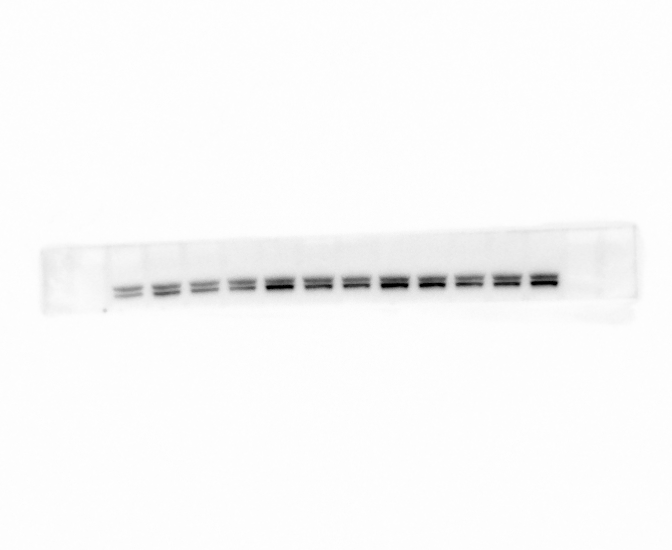

Supplement: Supplementary file 4 [file DataSheet2.ZIP › Original images and data in supplementary figures/Supplementary figure2/S2-PRO-CASP1.tif]

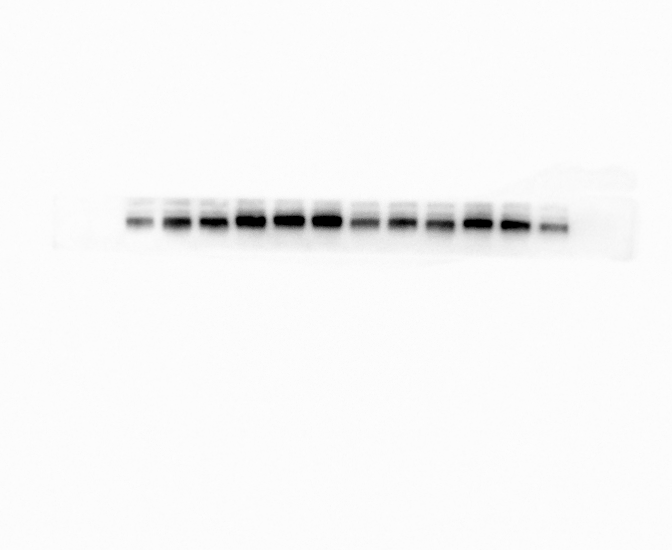

Supplement: Supplementary file 4 [file DataSheet2.ZIP › Original images and data in supplementary figures/Supplementary figure2/S2-TAU.tif]

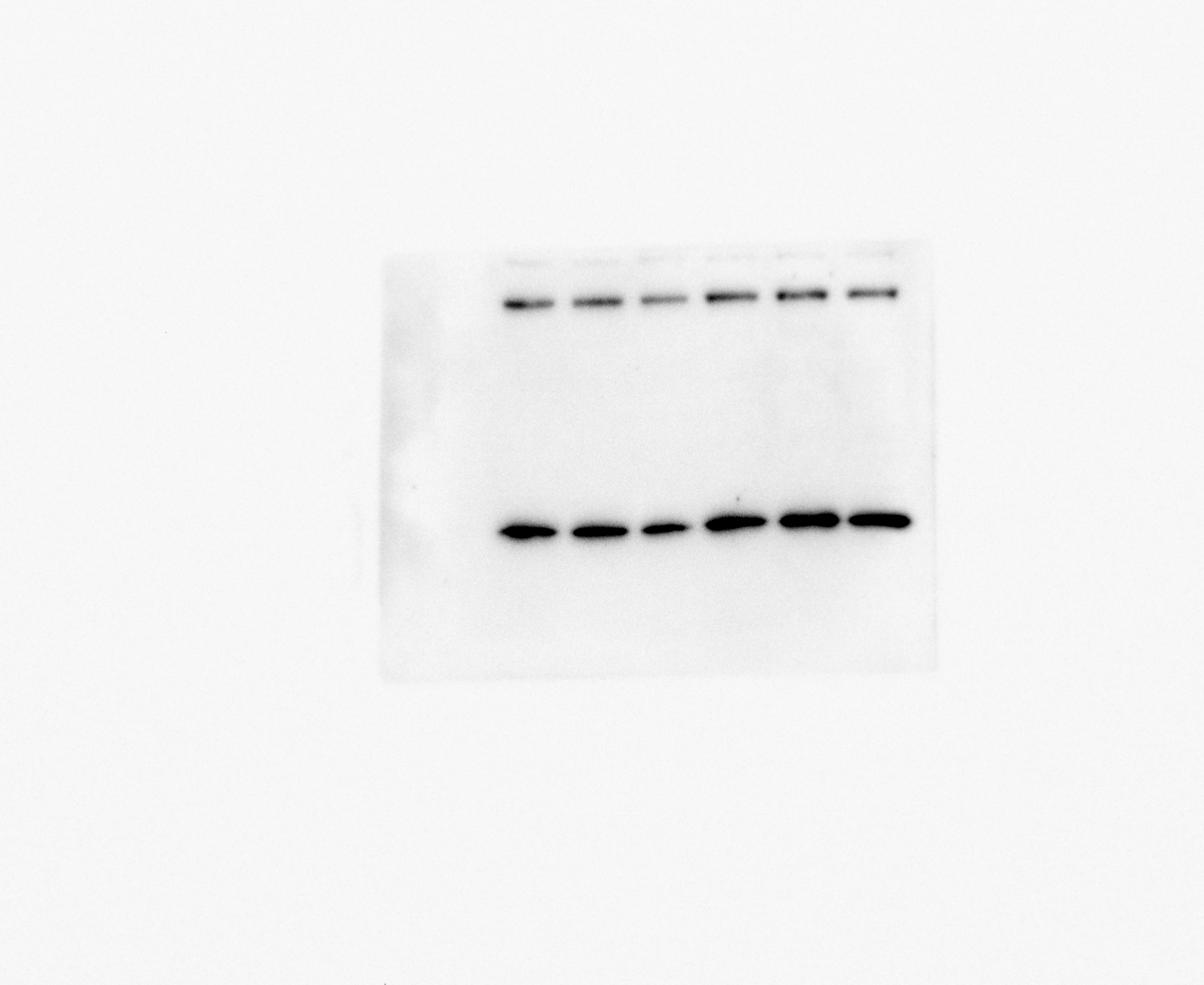

Supplement: Supplementary file 5 [file DataSheet5.ZIP › Original unedited images (western blot)/Figure1/Figure1A-Aβ.tif]

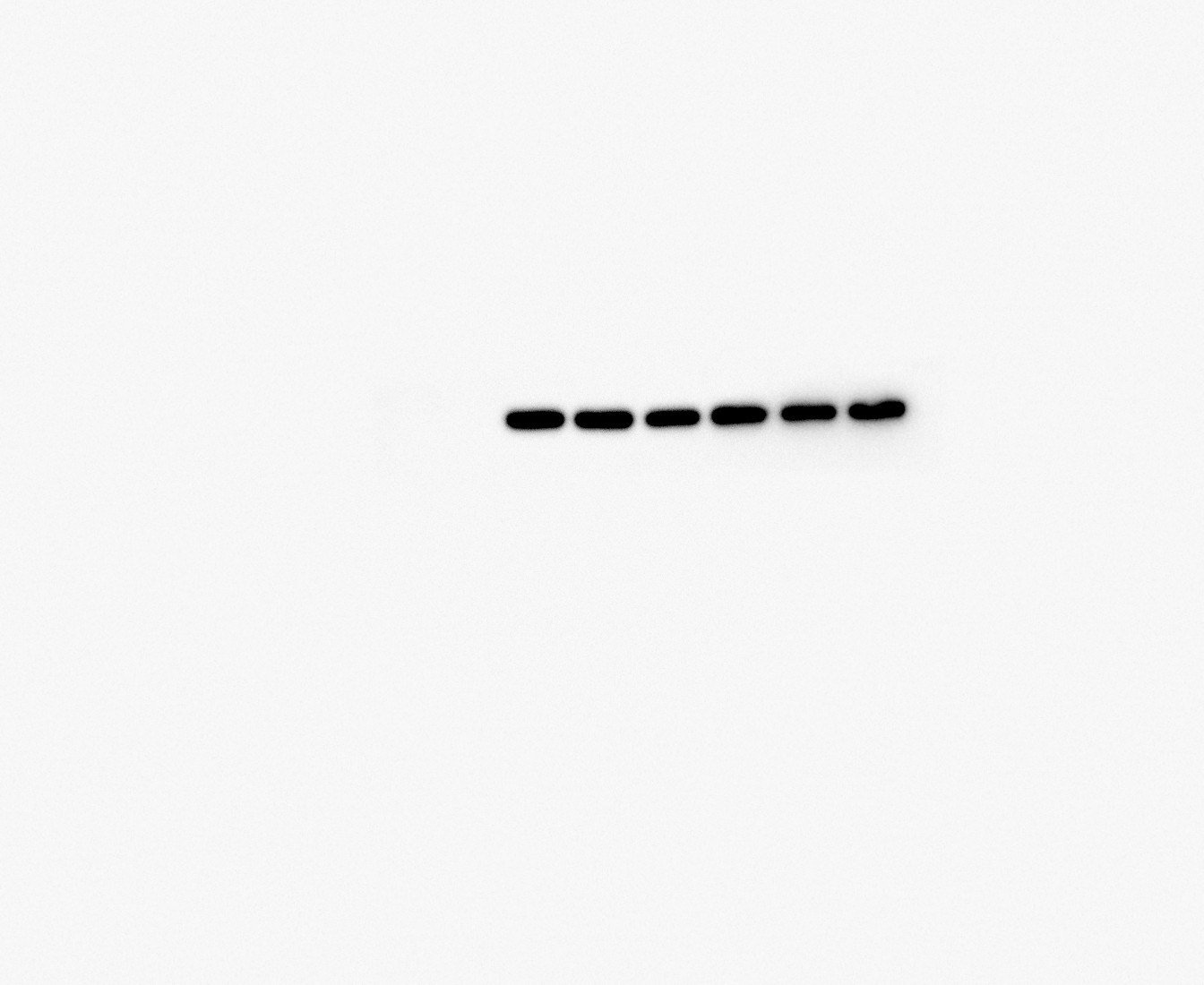

Supplement: Supplementary file 5 [file DataSheet5.ZIP › Original unedited images (western blot)/Figure1/Figure1A-GAPDH.tif]

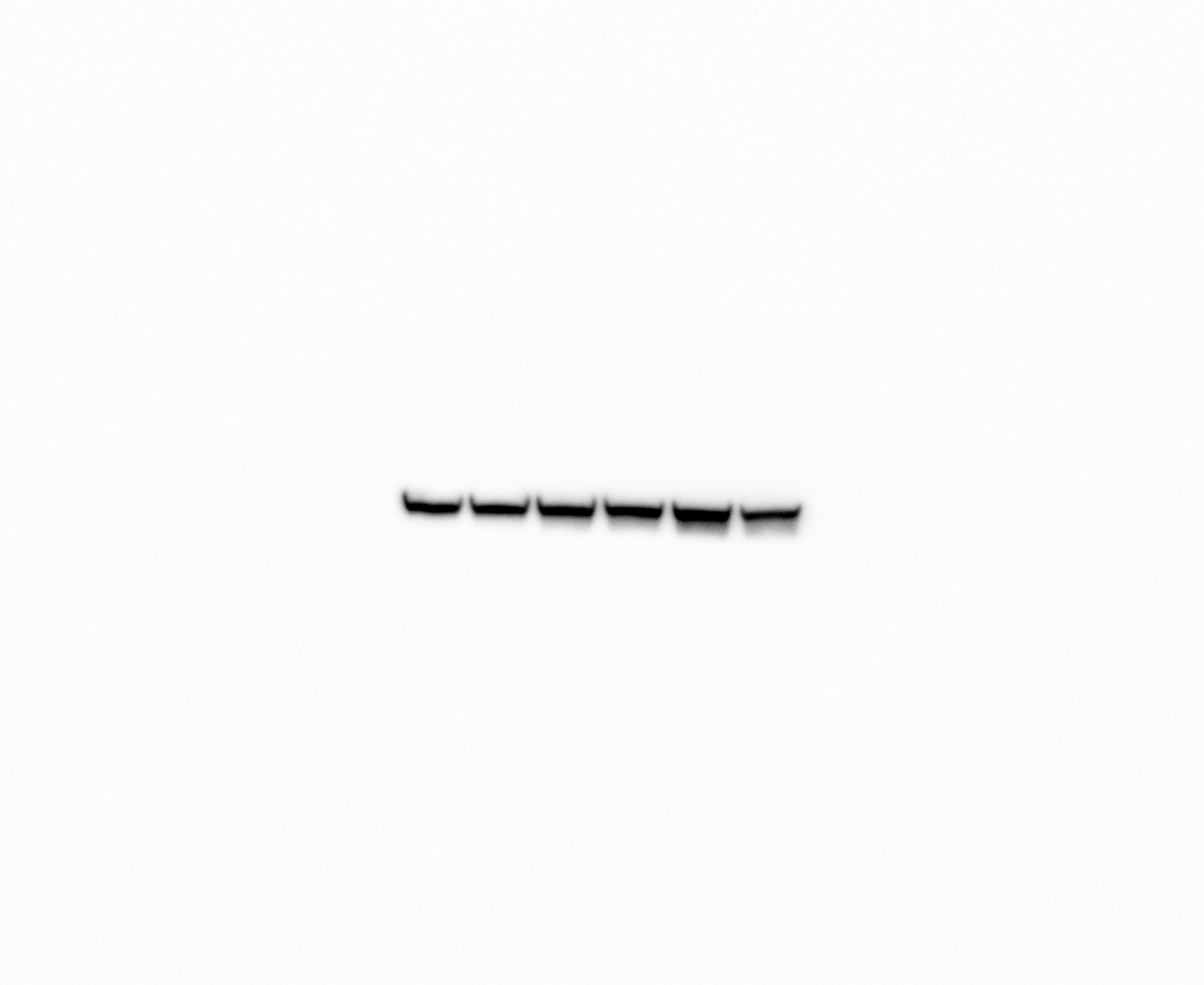

Supplement: Supplementary file 5 [file DataSheet5.ZIP › Original unedited images (western blot)/Figure1/Figure1B-CaMKII-α.tif]

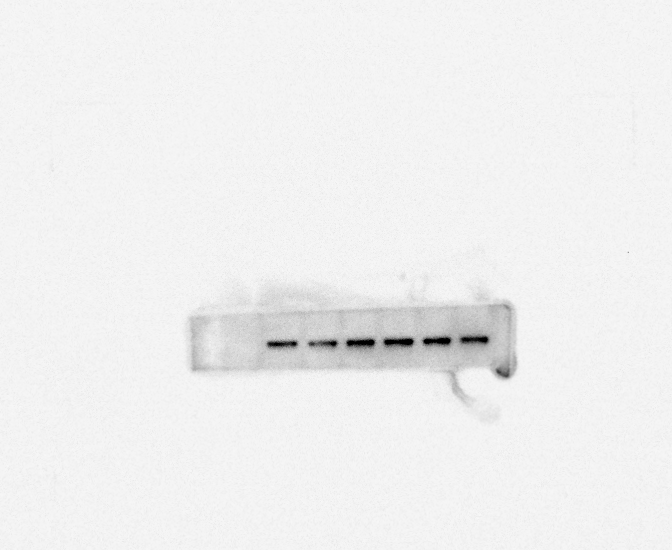

Supplement: Supplementary file 5 [file DataSheet5.ZIP › Original unedited images (western blot)/Figure1/Figure1B-demethylated PP2A subunit C.tif]

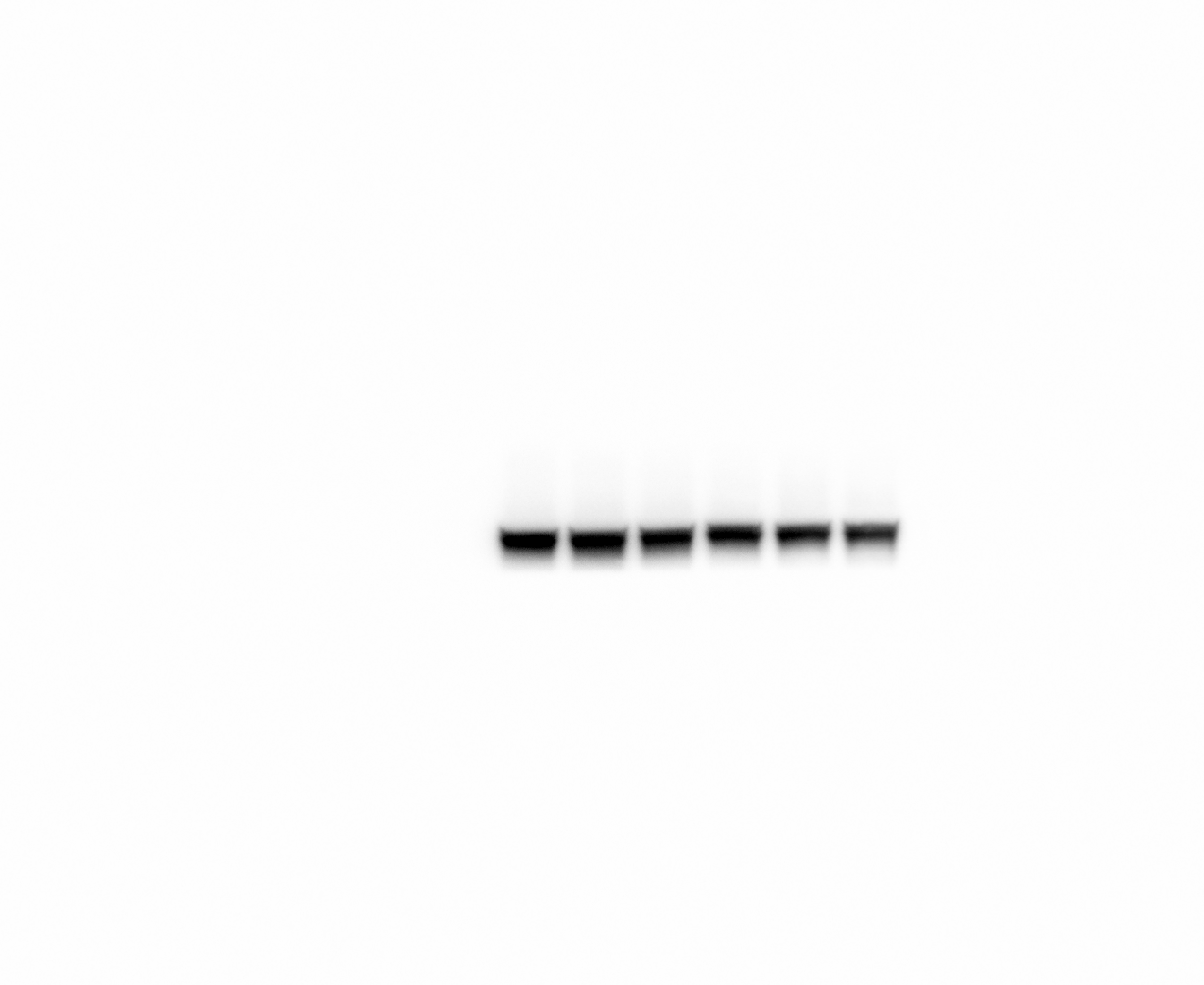

Supplement: Supplementary file 5 [file DataSheet5.ZIP › Original unedited images (western blot)/Figure1/Figure1B-GSK-3β.tif]

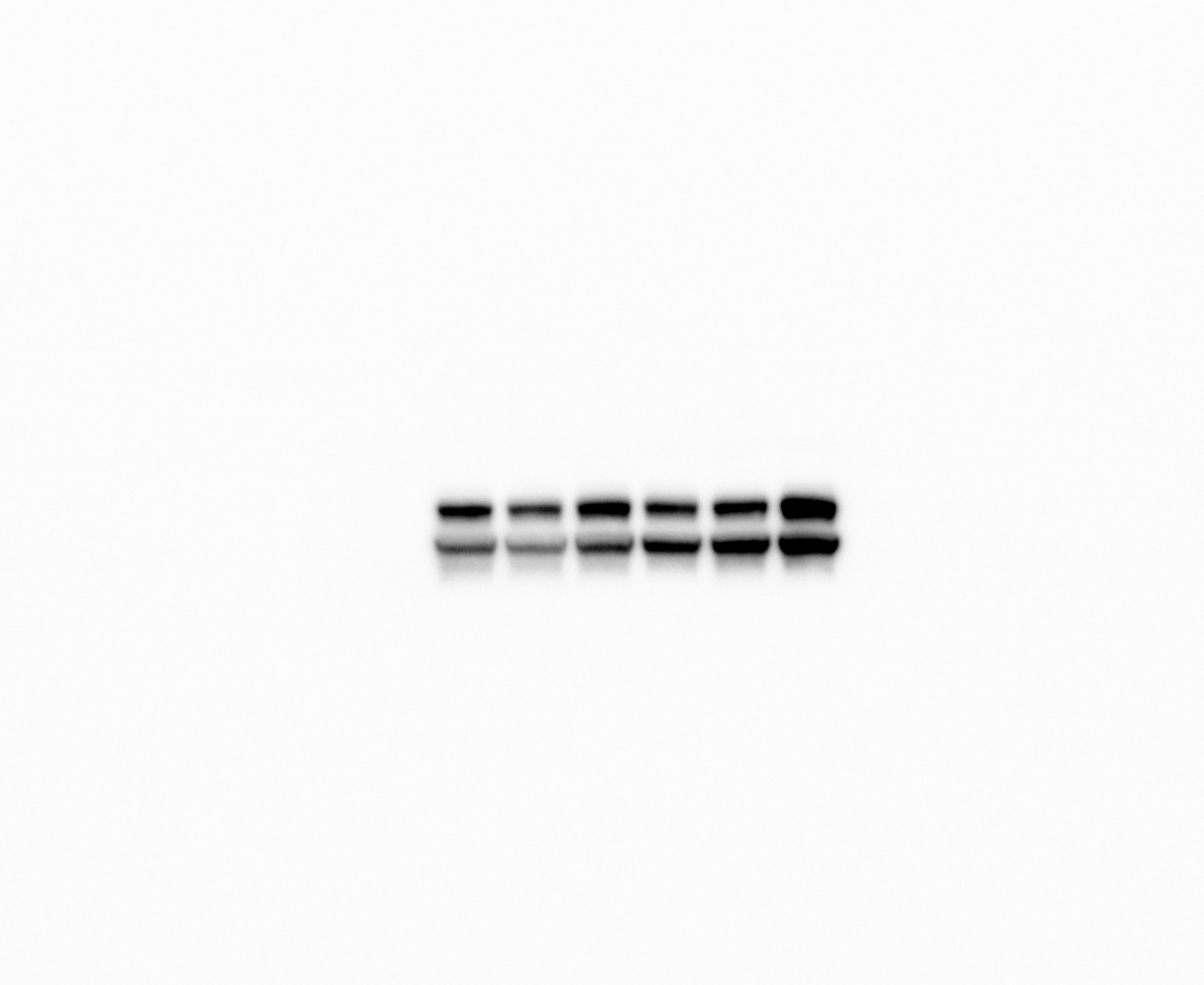

Supplement: Supplementary file 5 [file DataSheet5.ZIP › Original unedited images (western blot)/Figure1/Figure1B-p-CaMKII-α.tif]

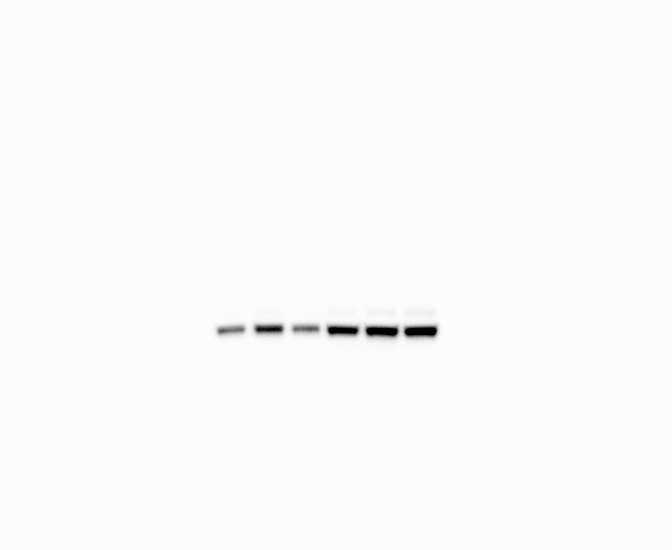

Supplement: Supplementary file 5 [file DataSheet5.ZIP › Original unedited images (western blot)/Figure1/Figure1B-p-GSK-3β.tif]

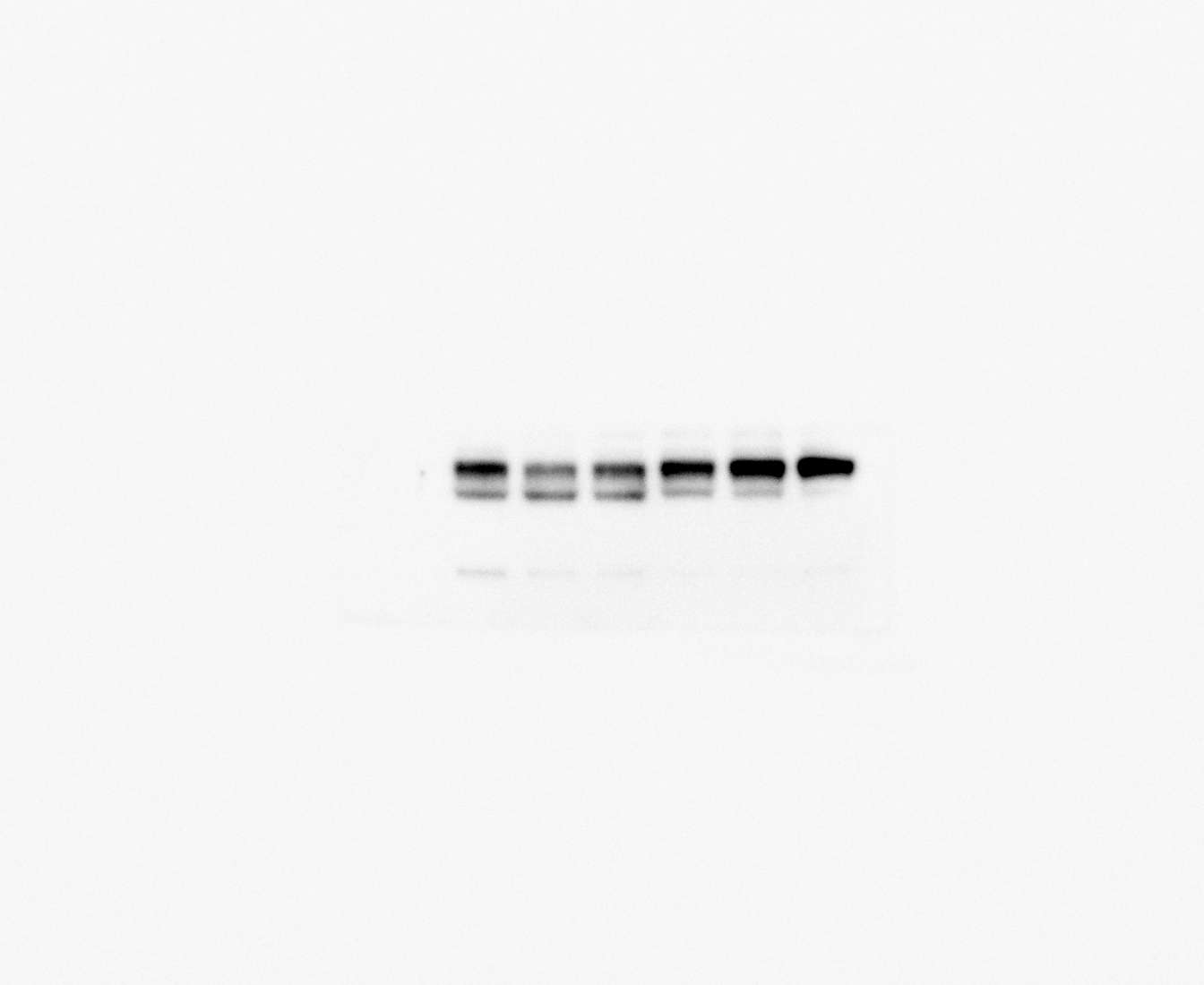

Supplement: Supplementary file 5 [file DataSheet5.ZIP › Original unedited images (western blot)/Figure1/Figure1B-P-tau.tif]

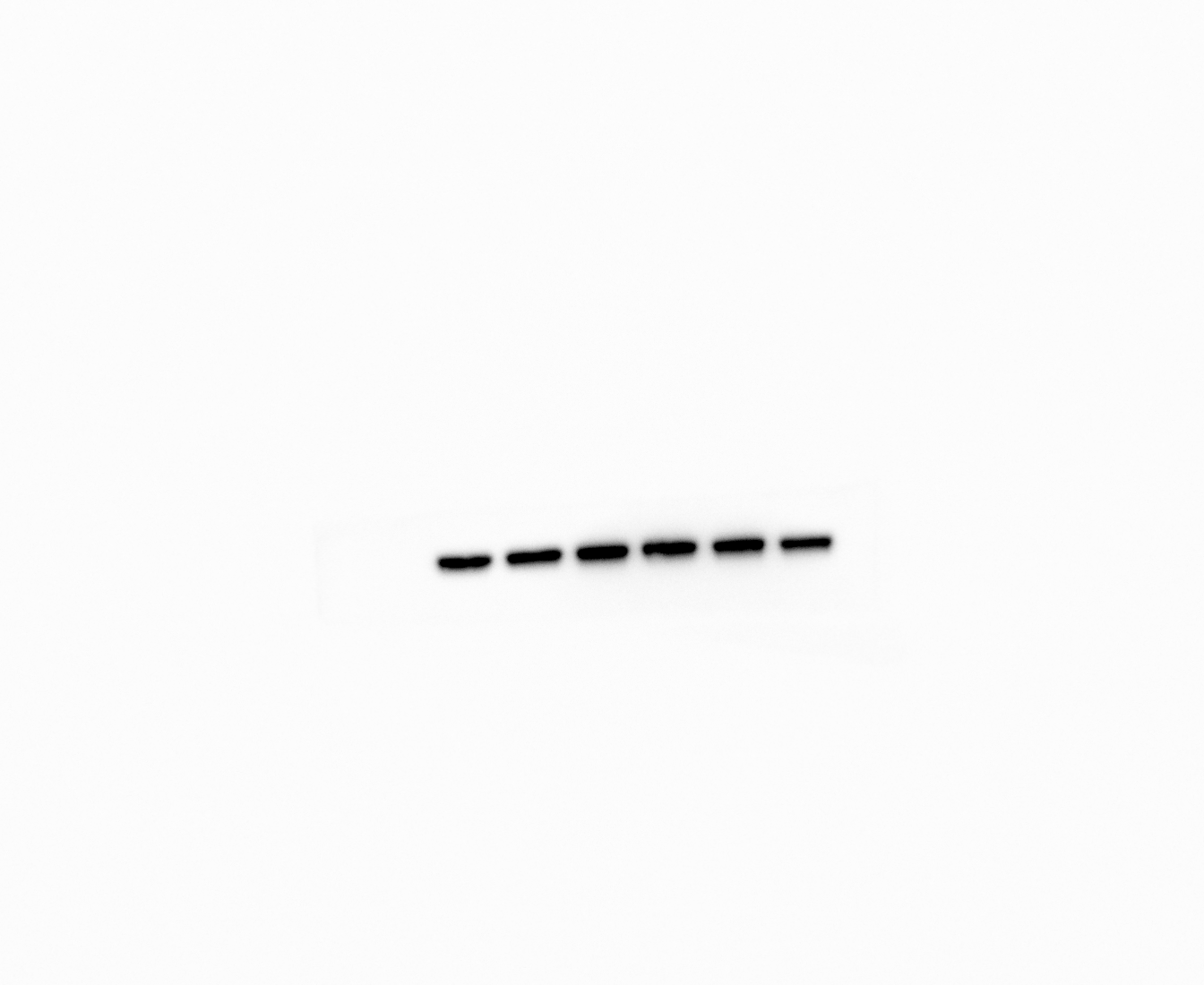

Supplement: Supplementary file 5 [file DataSheet5.ZIP › Original unedited images (western blot)/Figure1/Figure1B-PP2A.tif]

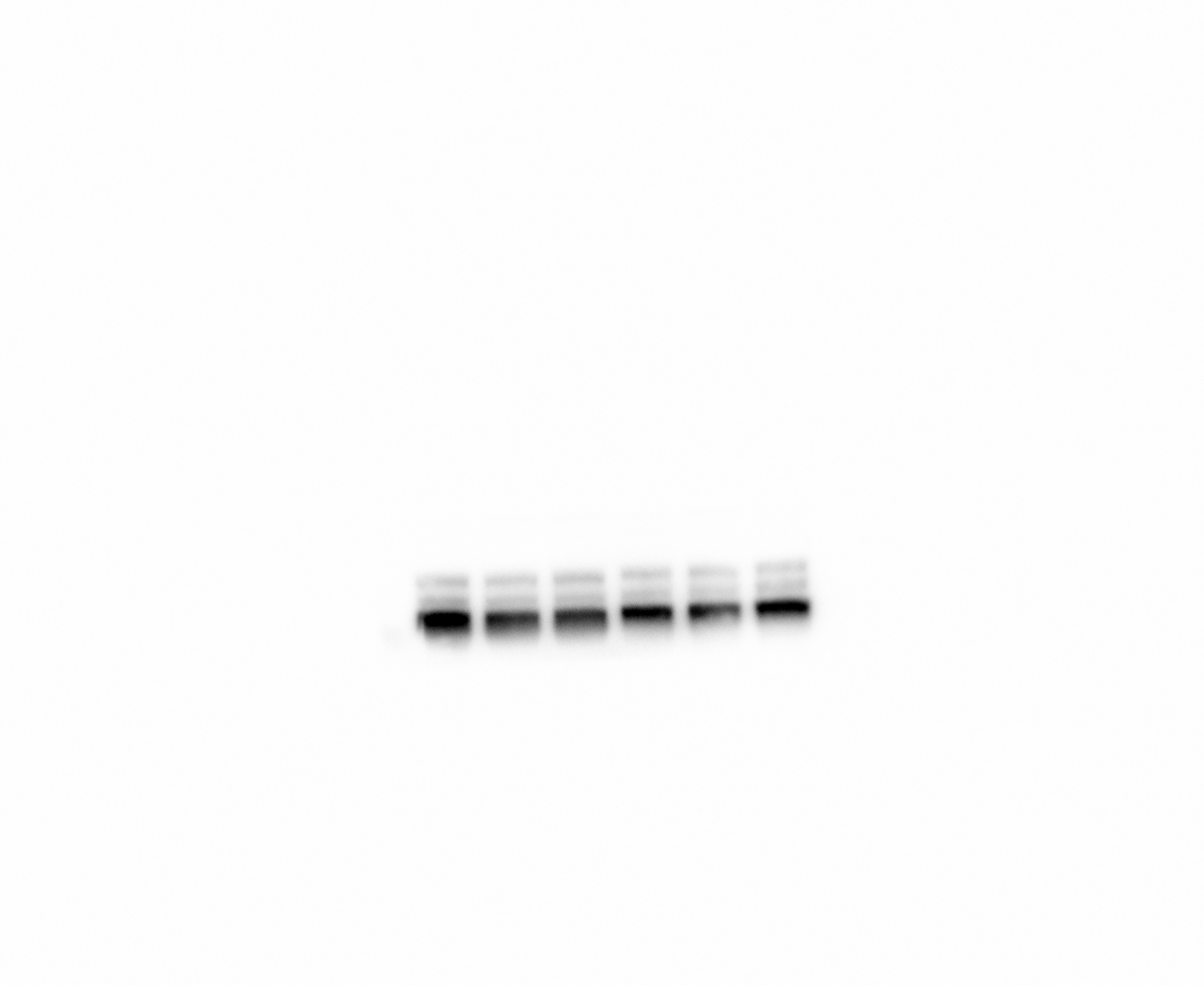

Supplement: Supplementary file 5 [file DataSheet5.ZIP › Original unedited images (western blot)/Figure1/Figure1B-Tau.tif]

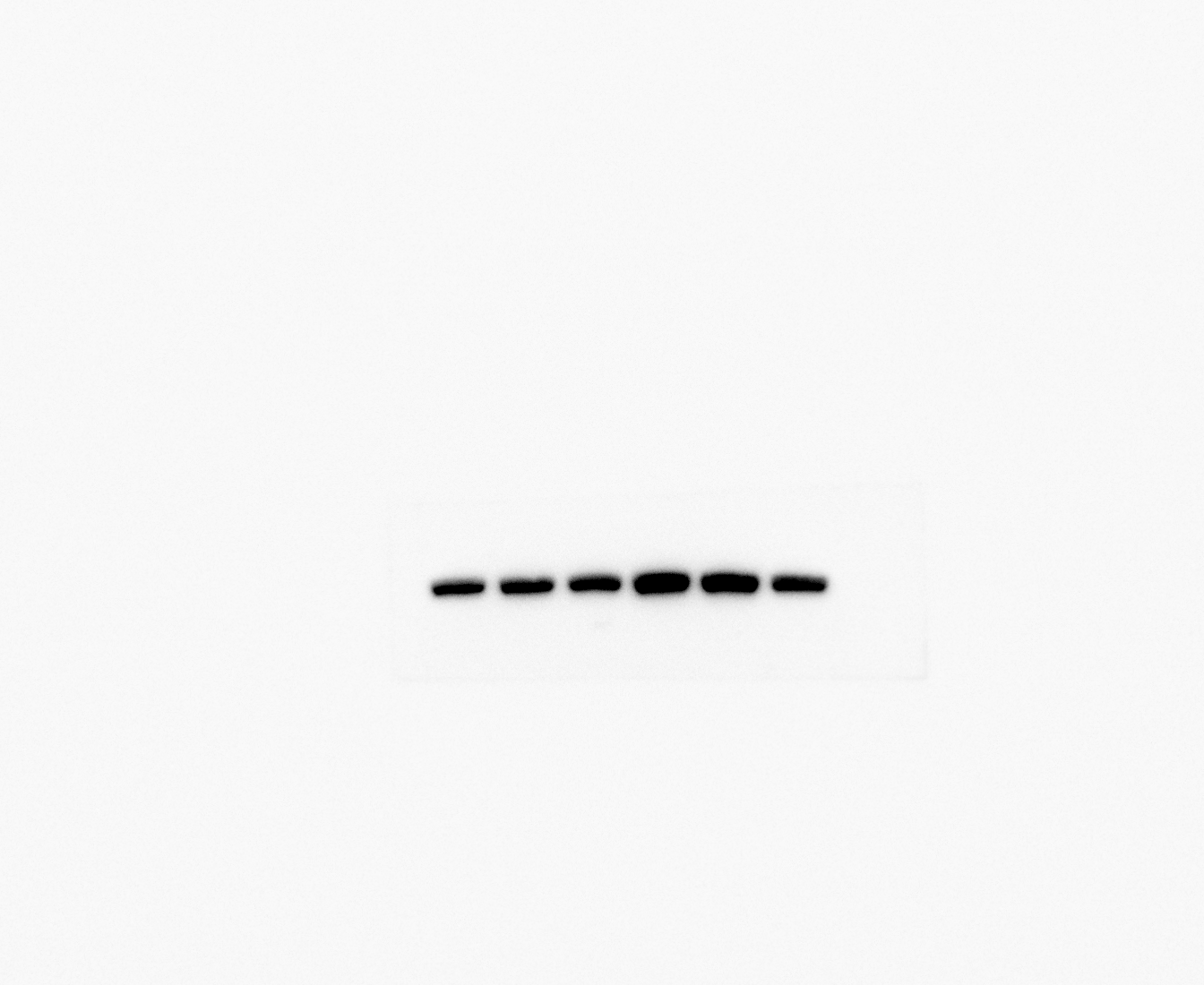

Supplement: Supplementary file 5 [file DataSheet5.ZIP › Original unedited images (western blot)/Figure1/Figure1D-AIM2.tif]

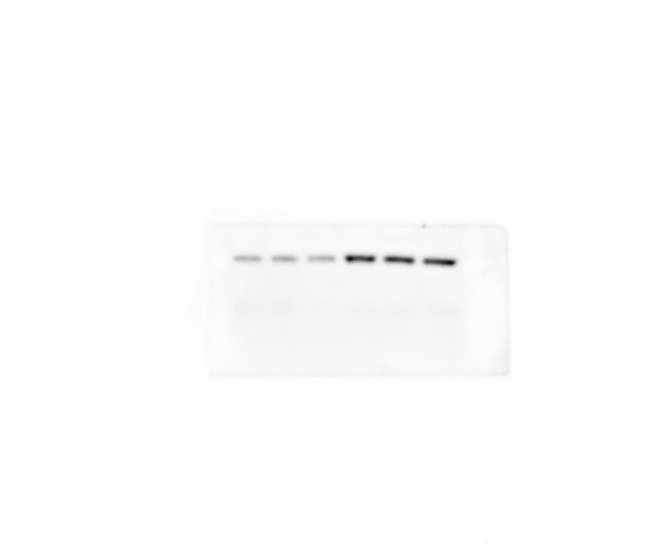

Supplement: Supplementary file 5 [file DataSheet5.ZIP › Original unedited images (western blot)/Figure1/Figure1D-ASC.tif]

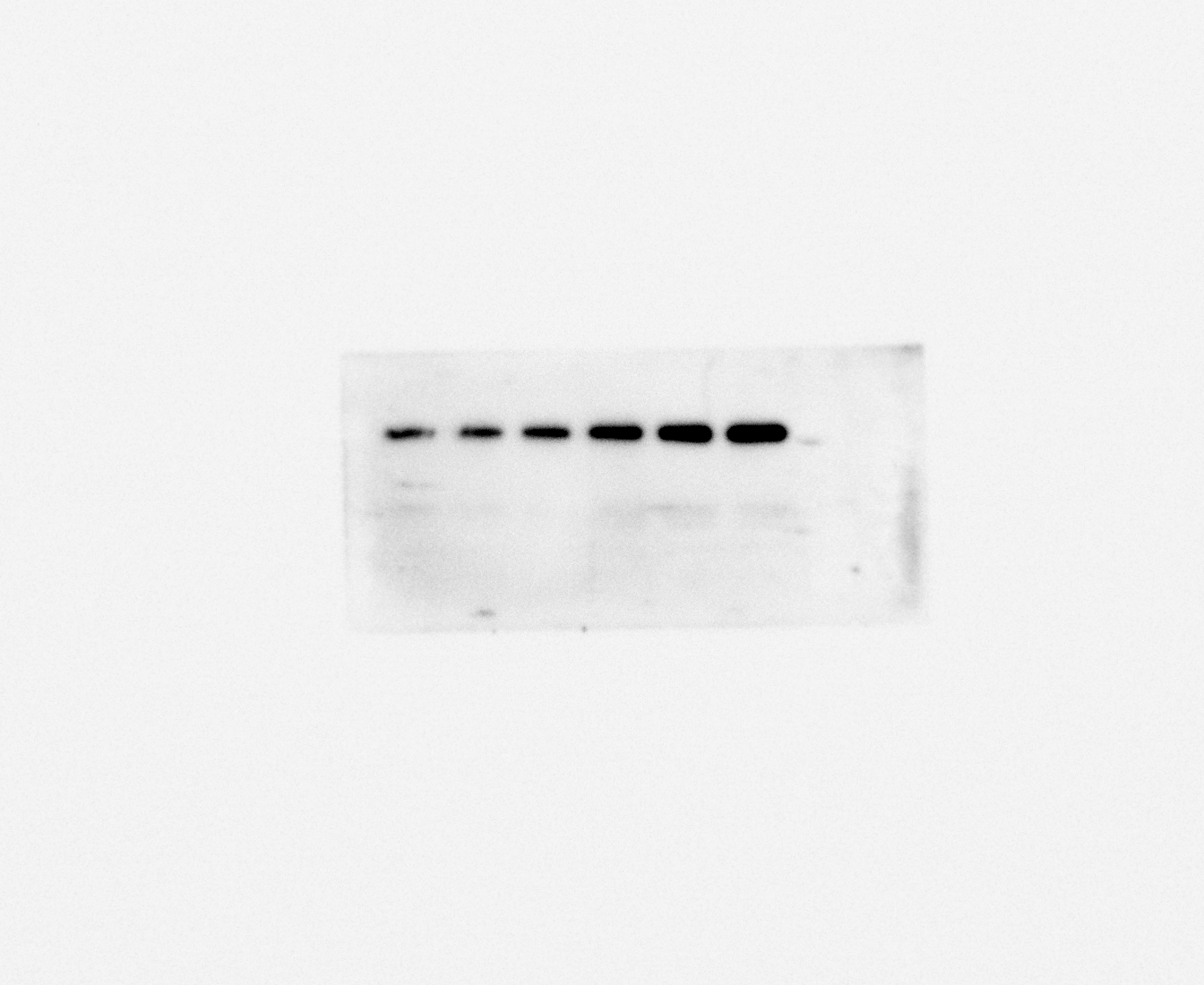

Supplement: Supplementary file 5 [file DataSheet5.ZIP › Original unedited images (western blot)/Figure1/Figure1D-cleaved caspase1.tif]

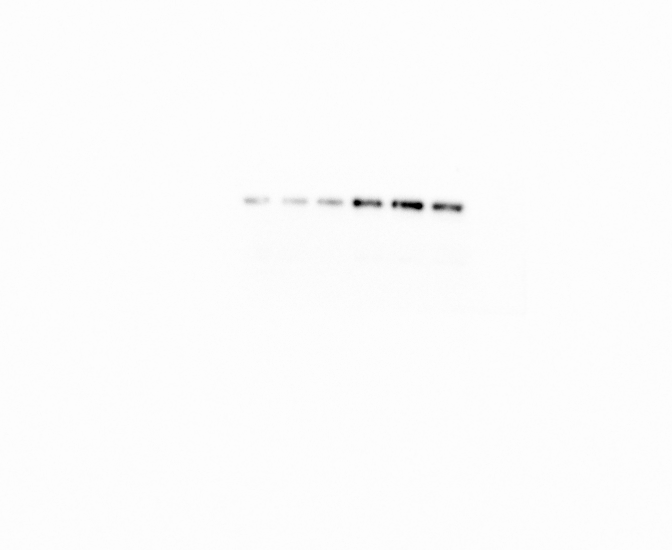

Supplement: Supplementary file 5 [file DataSheet5.ZIP › Original unedited images (western blot)/Figure1/Figure1D-cleaved GSDMD.tif]

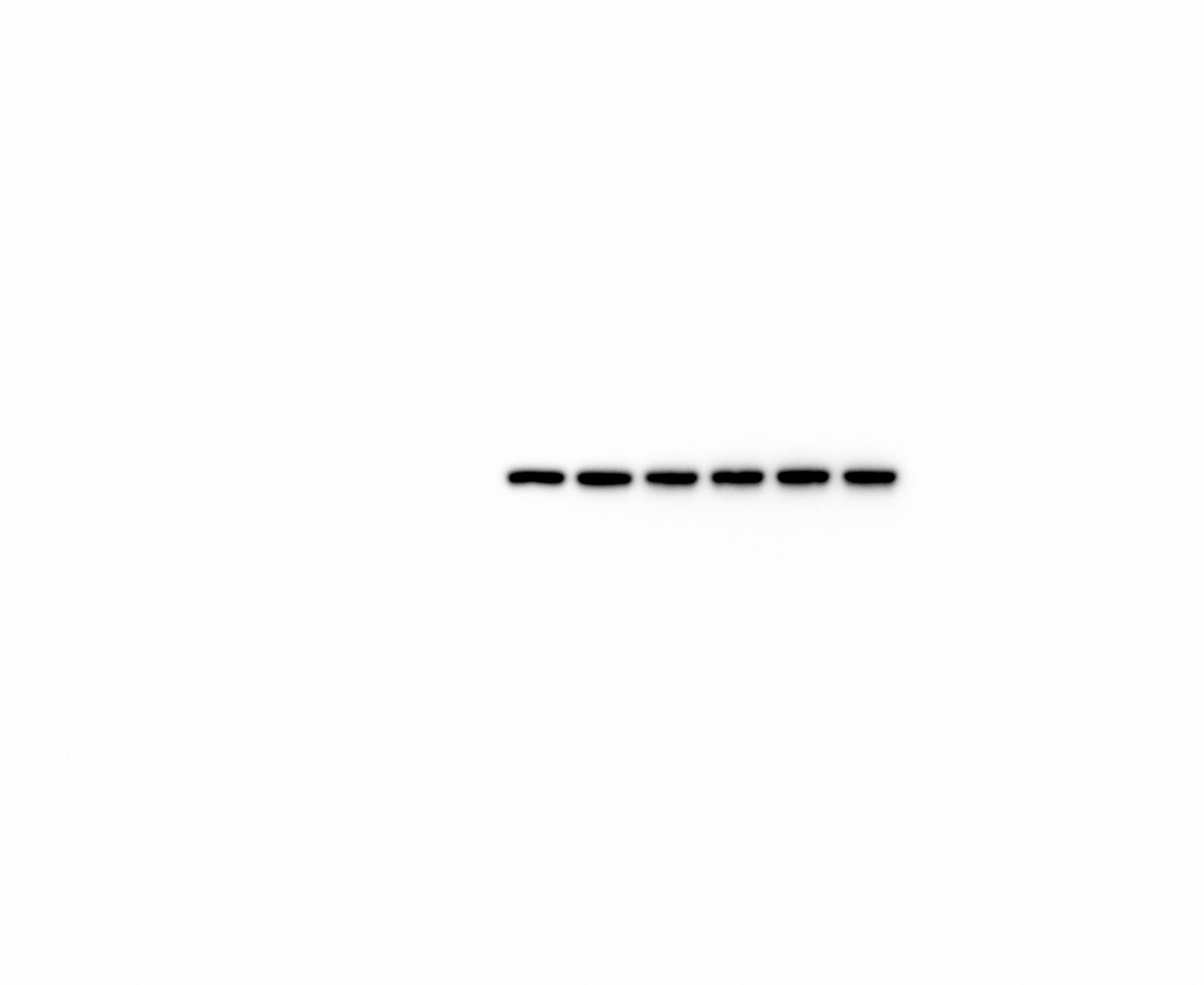

Supplement: Supplementary file 5 [file DataSheet5.ZIP › Original unedited images (western blot)/Figure1/Figure1D-GAPDH.tif]

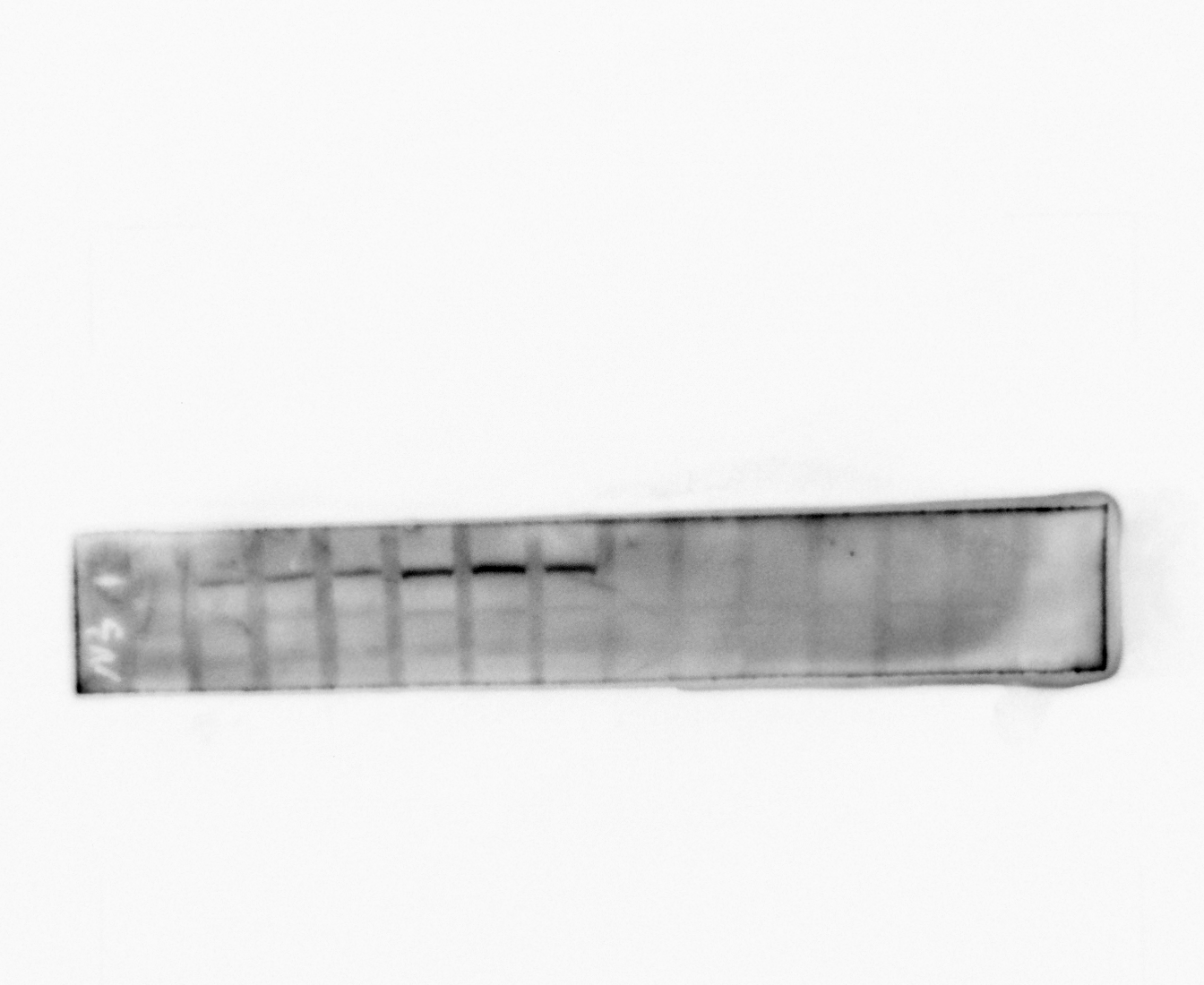

Supplement: Supplementary file 5 [file DataSheet5.ZIP › Original unedited images (western blot)/Figure1/Figure1D-NLRP3.tif]

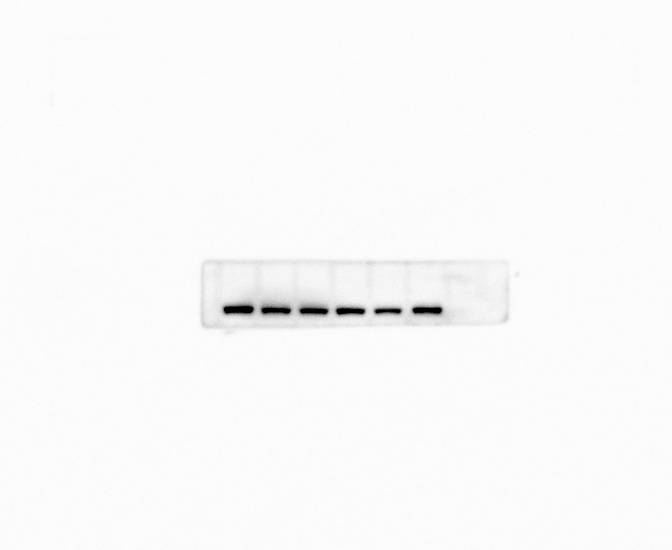

Supplement: Supplementary file 5 [file DataSheet5.ZIP › Original unedited images (western blot)/Figure1/Figure1D-Pro-caspase1.tif]

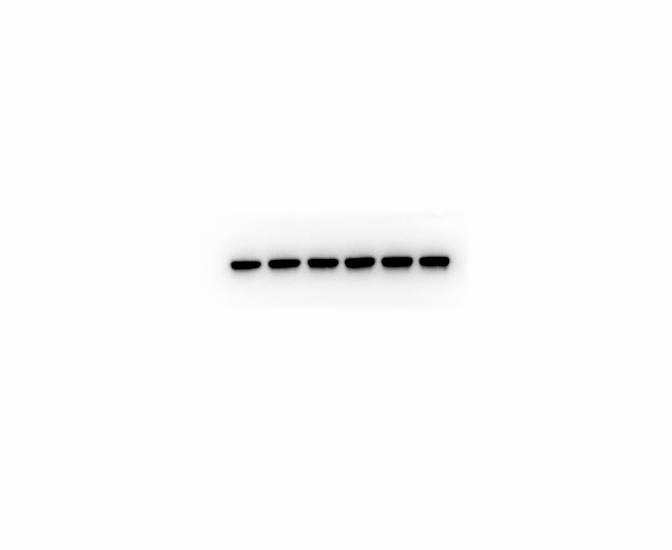

Supplement: Supplementary file 5 [file DataSheet5.ZIP › Original unedited images (western blot)/Figure1/Figure1F-GAPDH.tif]

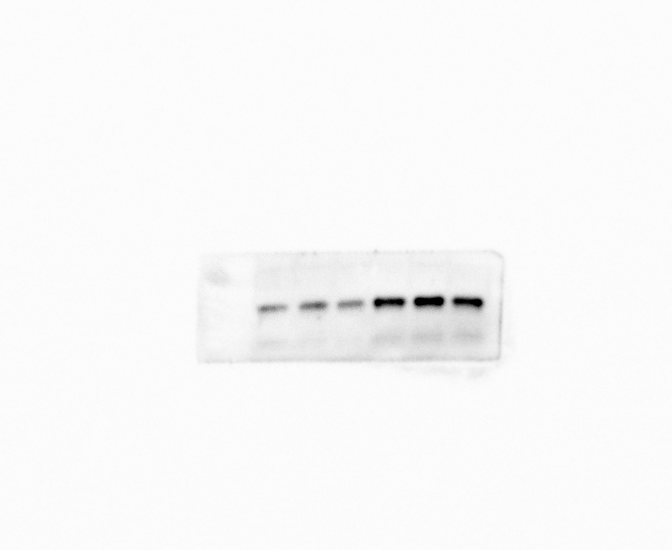

Supplement: Supplementary file 5 [file DataSheet5.ZIP › Original unedited images (western blot)/Figure1/Figure1F-IL-18.tif]

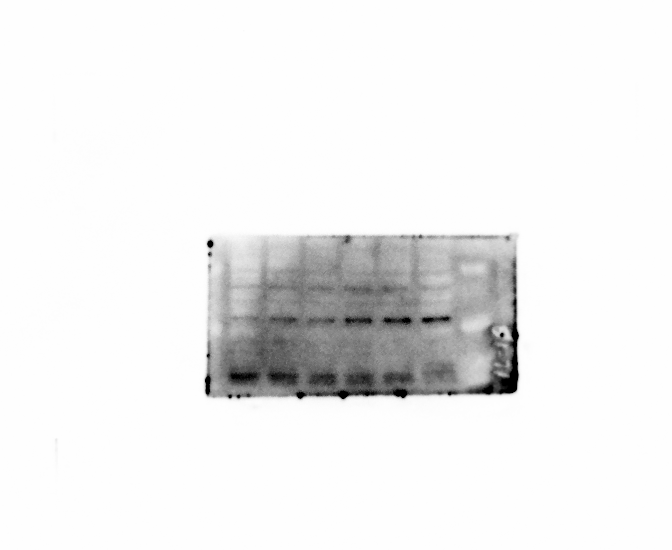

Supplement: Supplementary file 5 [file DataSheet5.ZIP › Original unedited images (western blot)/Figure1/Figure1F-IL-1β.tif]

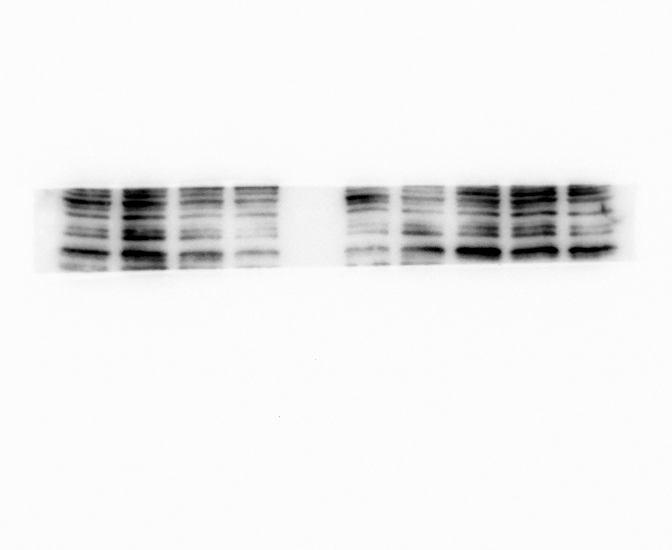

Supplement: Supplementary file 5 [file DataSheet5.ZIP › Original unedited images (western blot)/Figure2/Figure2A and 2B-ASC.tif]

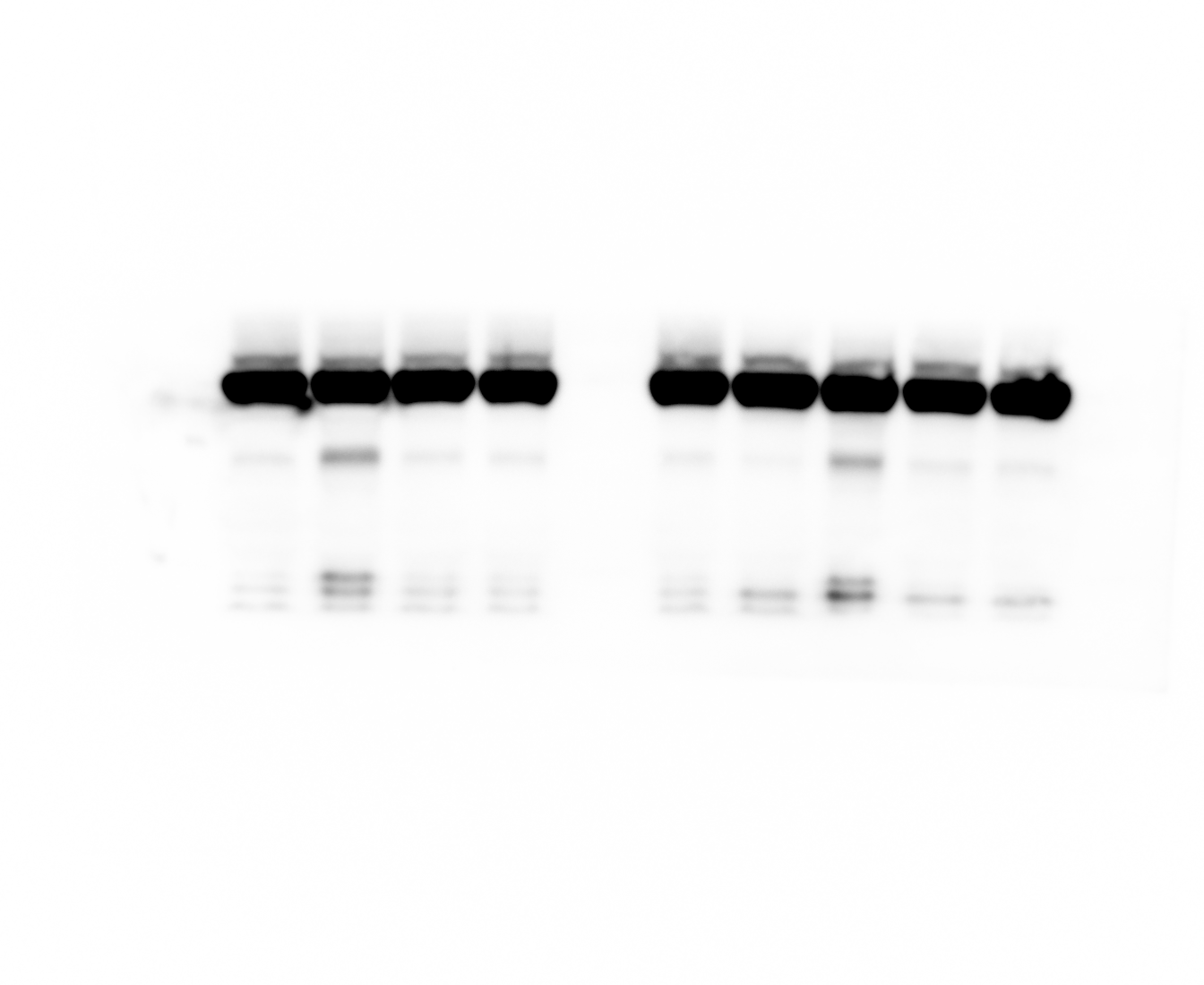

Supplement: Supplementary file 5 [file DataSheet5.ZIP › Original unedited images (western blot)/Figure2/Figure2A and 2B-casp3.tif]

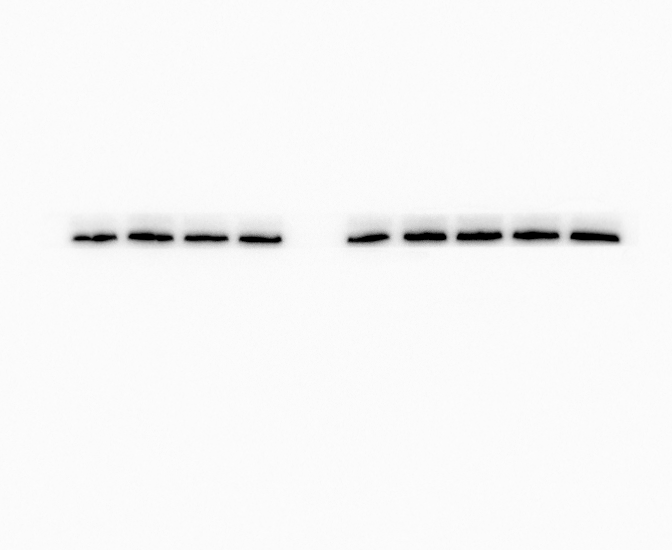

Supplement: Supplementary file 5 [file DataSheet5.ZIP › Original unedited images (western blot)/Figure2/Figure2A and 2B-GAPDH.tif]

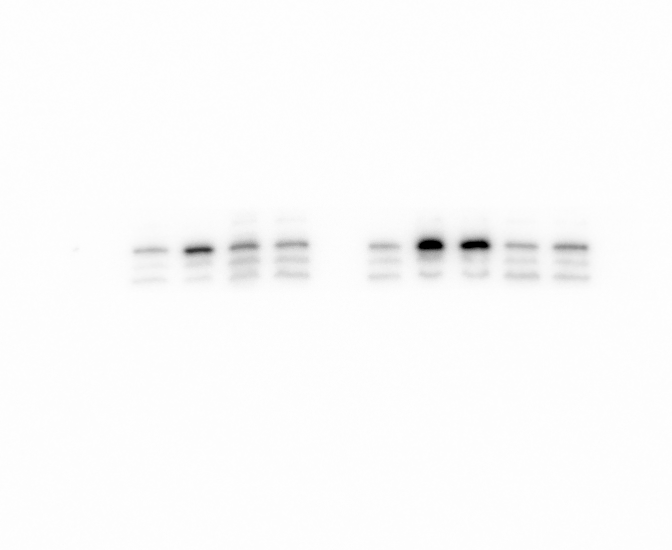

Supplement: Supplementary file 5 [file DataSheet5.ZIP › Original unedited images (western blot)/Figure2/Figure2A and 2B-IL-18.tif]

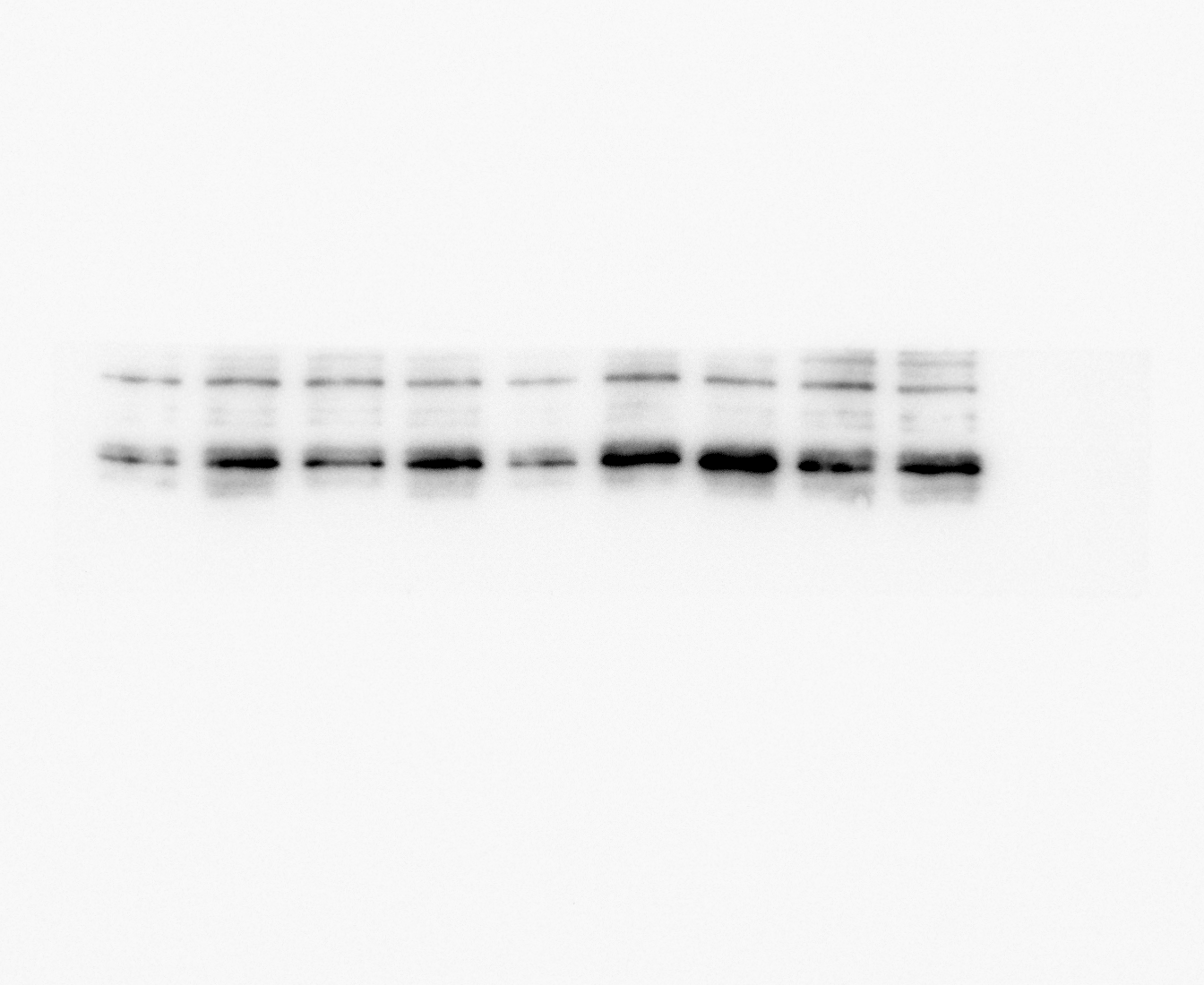

Supplement: Supplementary file 5 [file DataSheet5.ZIP › Original unedited images (western blot)/Figure2/Figure2A and 2B-IL-1β.tif]

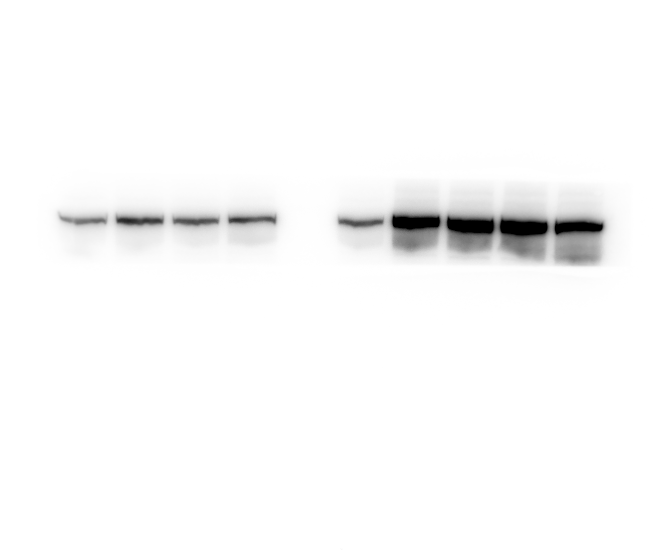

Supplement: Supplementary file 5 [file DataSheet5.ZIP › Original unedited images (western blot)/Figure2/Figure2A and 2B-NLRP3.tif]

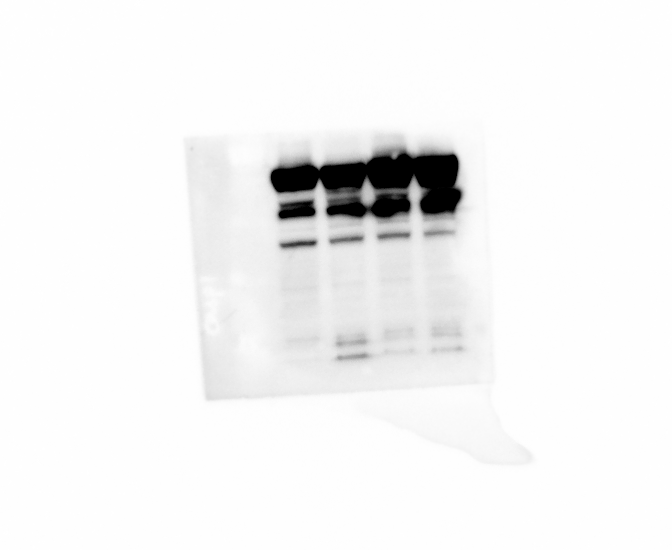

Supplement: Supplementary file 5 [file DataSheet5.ZIP › Original unedited images (western blot)/Figure2/Figure2A-CASP1.tif]

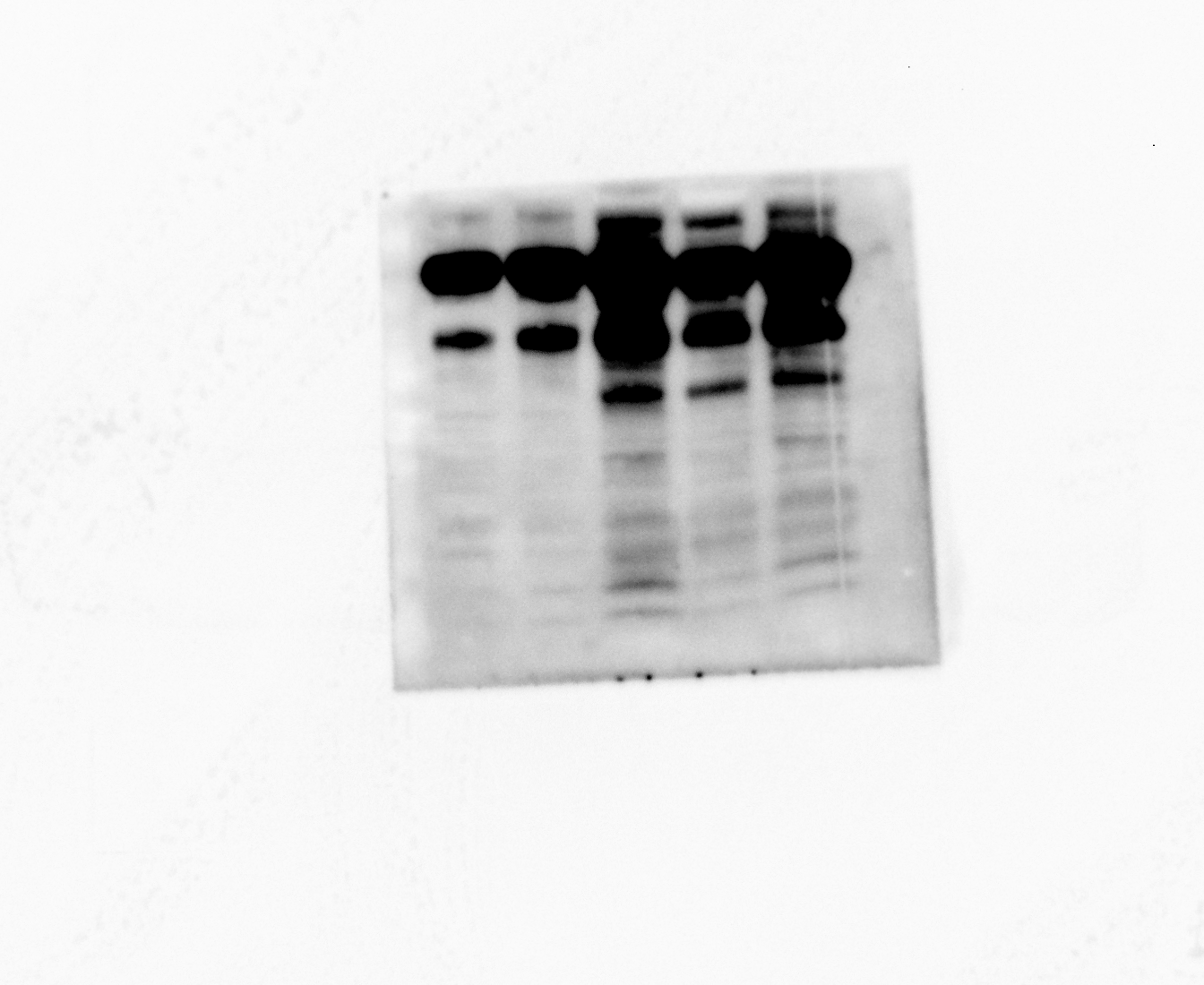

Supplement: Supplementary file 5 [file DataSheet5.ZIP › Original unedited images (western blot)/Figure2/Figure2B-casp1.tif]

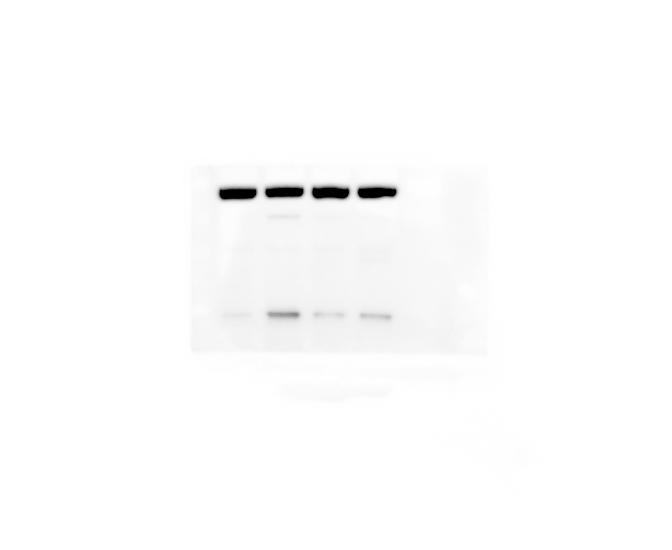

Supplement: Supplementary file 5 [file DataSheet5.ZIP › Original unedited images (western blot)/Figure2/Figure2C-GSDMD(left).tif]

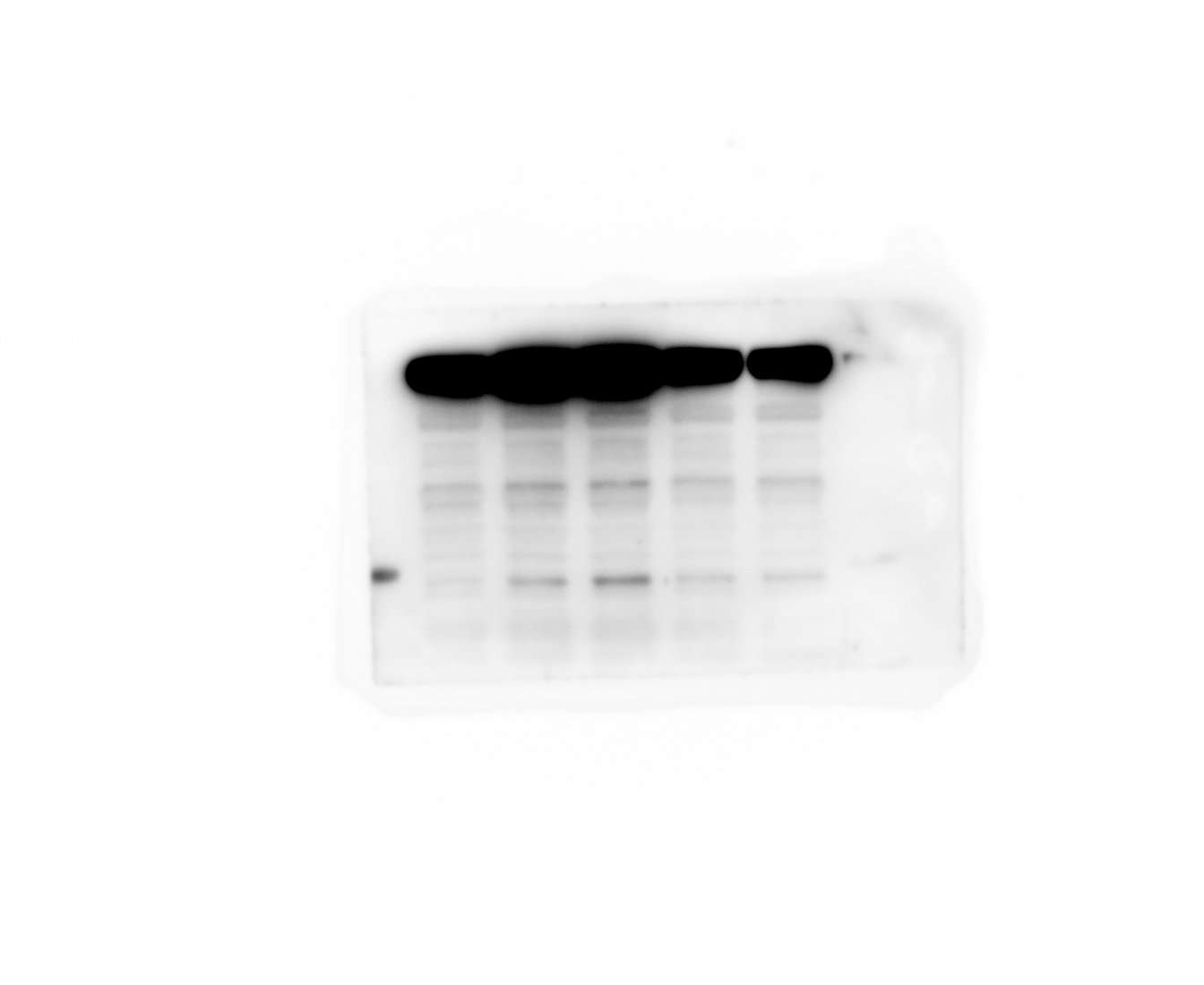

Supplement: Supplementary file 5 [file DataSheet5.ZIP › Original unedited images (western blot)/Figure2/Figure2C-GSDMD(right).tif]

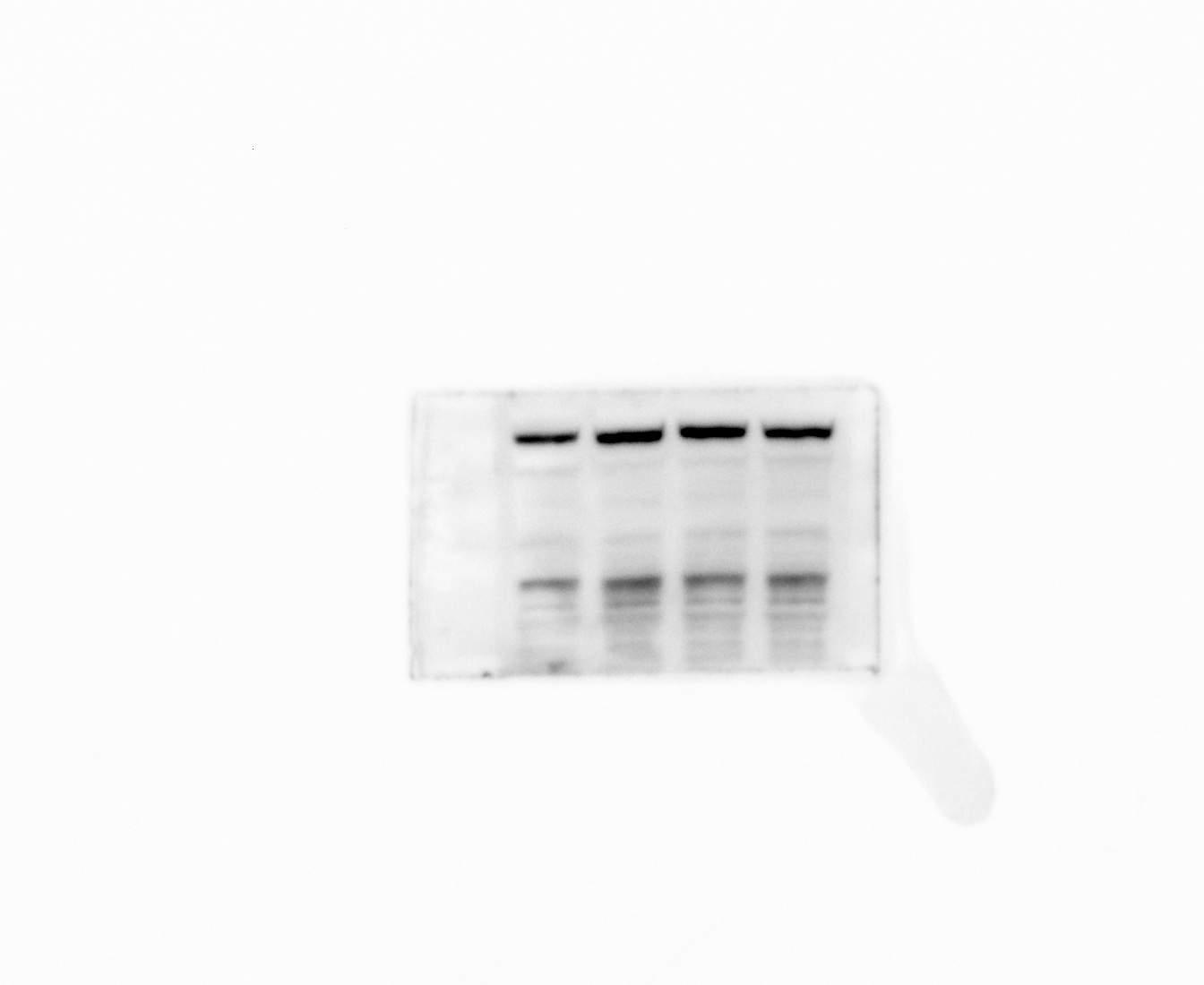

Supplement: Supplementary file 5 [file DataSheet5.ZIP › Original unedited images (western blot)/Figure2/Figure2C-GSDME(left).tif]

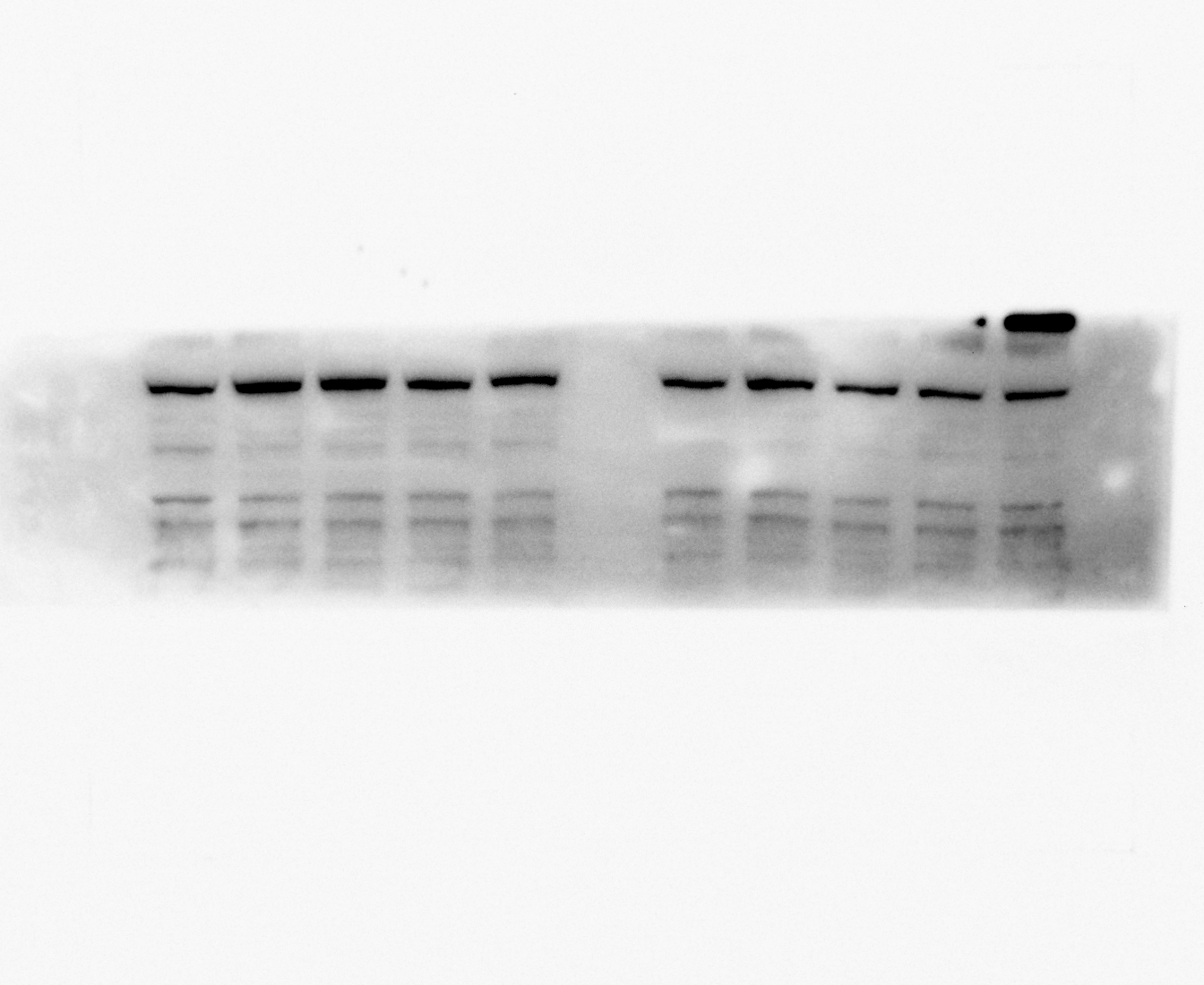

Supplement: Supplementary file 5 [file DataSheet5.ZIP › Original unedited images (western blot)/Figure2/Figure2C-GSDME.tif]

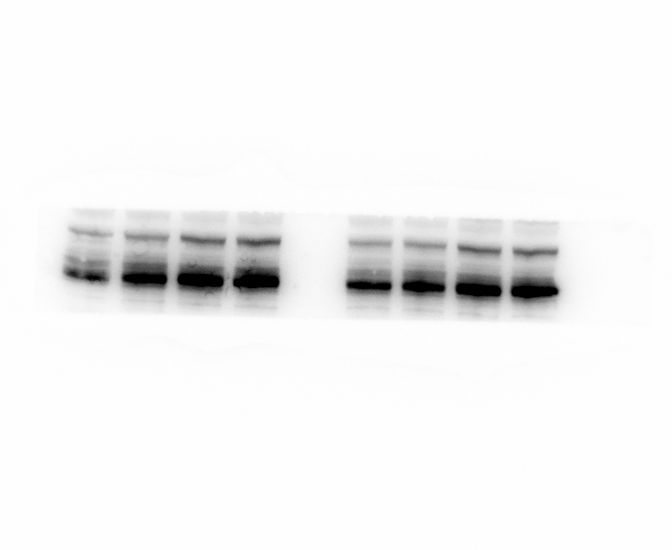

Supplement: Supplementary file 5 [file DataSheet5.ZIP › Original unedited images (western blot)/Figure3/Figure3A-AIM2.tif]

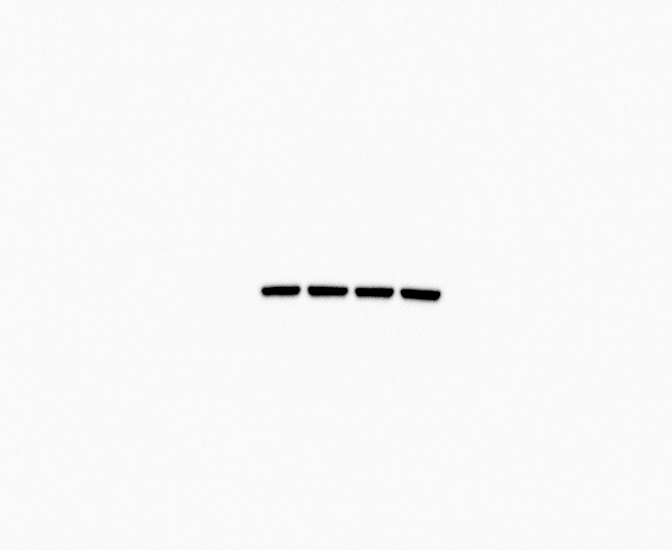

Supplement: Supplementary file 5 [file DataSheet5.ZIP › Original unedited images (western blot)/Figure3/Figure3A-GAPDH.tif]

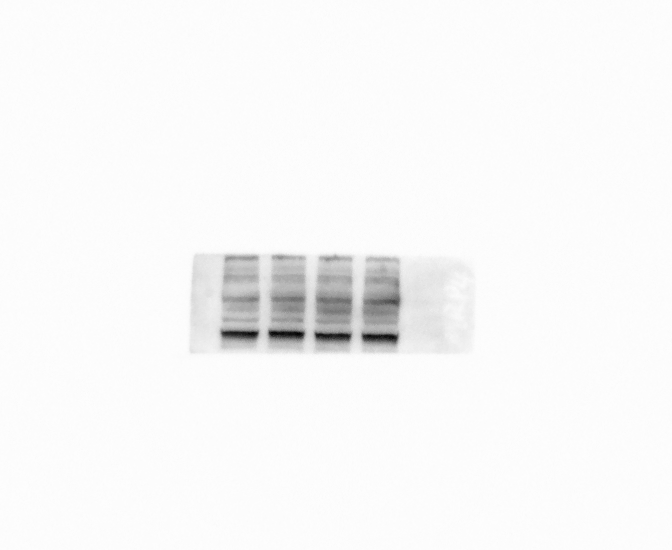

Supplement: Supplementary file 5 [file DataSheet5.ZIP › Original unedited images (western blot)/Figure3/Figure3A-NLRC4.tif]

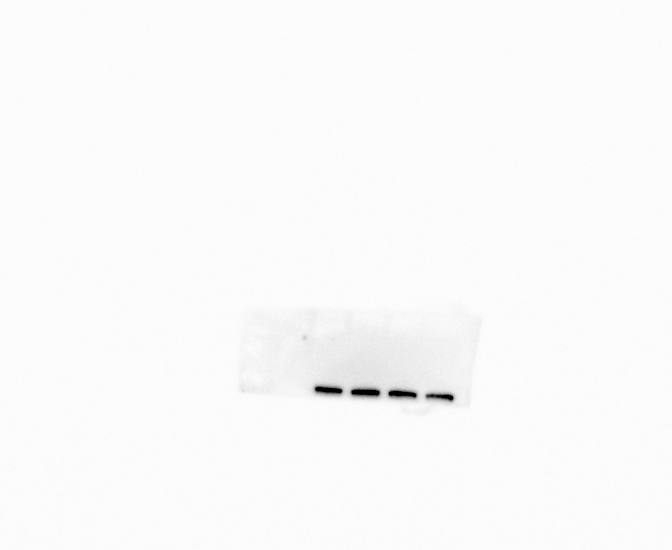

Supplement: Supplementary file 5 [file DataSheet5.ZIP › Original unedited images (western blot)/Figure3/Figure3A-NLRP1.tif]

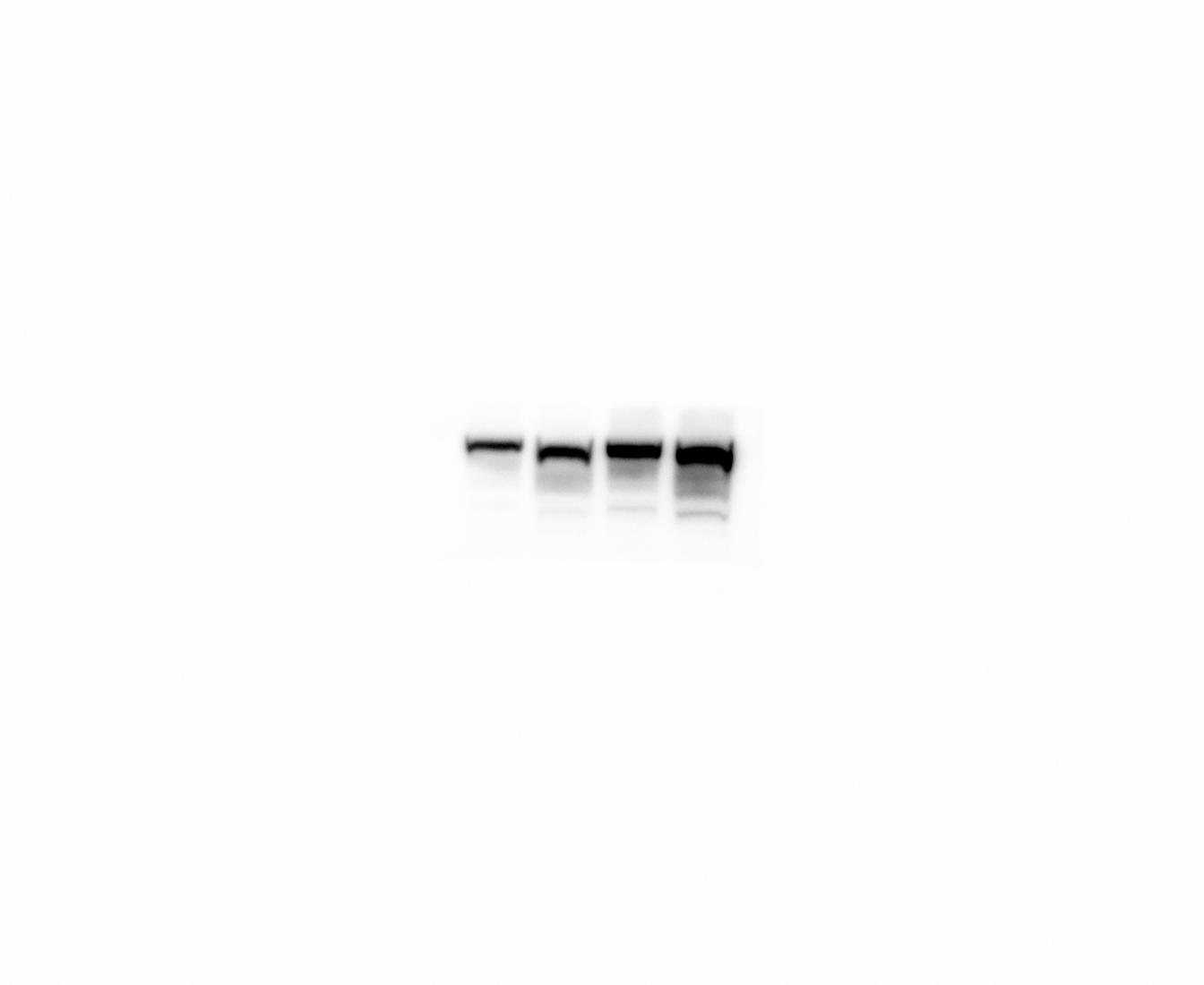

Supplement: Supplementary file 5 [file DataSheet5.ZIP › Original unedited images (western blot)/Figure3/Figure3A-NLRP3.tif]

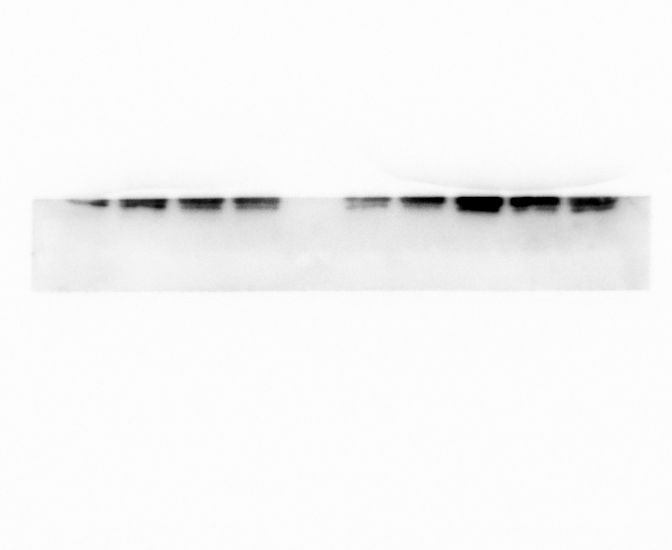

Supplement: Supplementary file 5 [file DataSheet5.ZIP › Original unedited images (western blot)/Figure4/Figure4A and 4C-Aβ.tif]

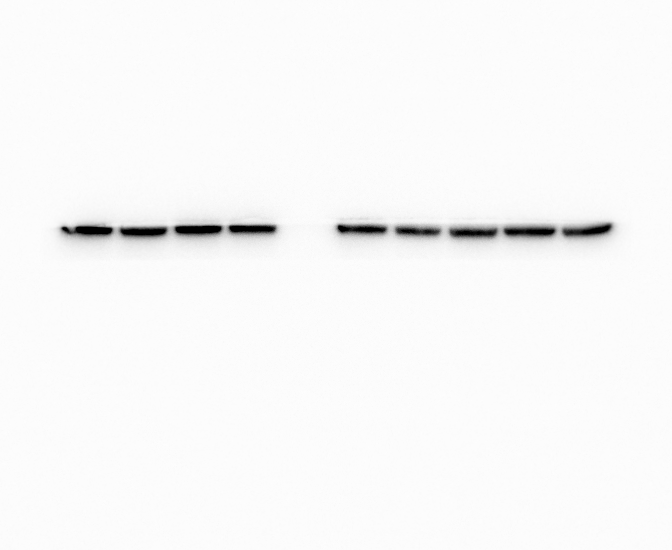

Supplement: Supplementary file 5 [file DataSheet5.ZIP › Original unedited images (western blot)/Figure4/Figure4A and 4C-GAPDH.tif]

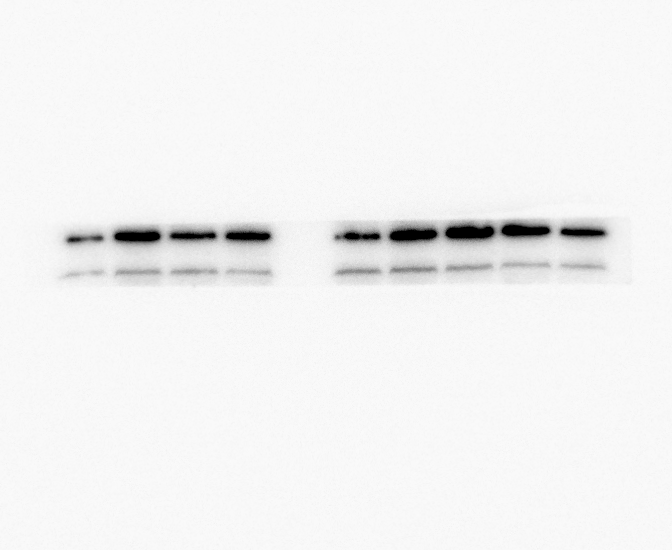

Supplement: Supplementary file 5 [file DataSheet5.ZIP › Original unedited images (western blot)/Figure4/Figure4A and 4C-P-TAU.tif]

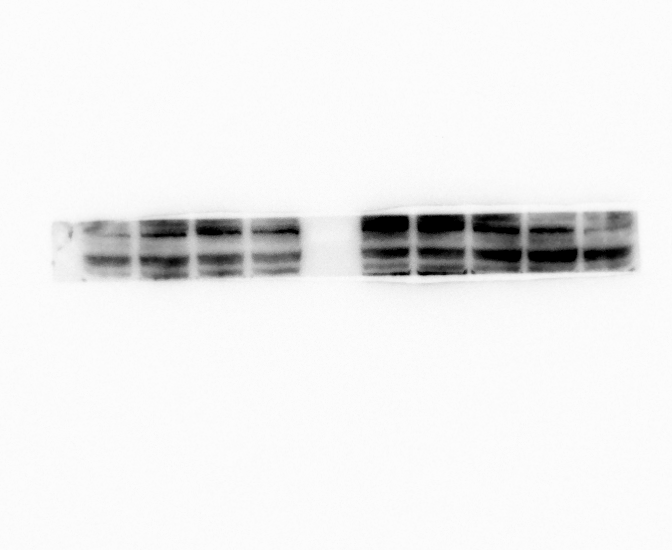

Supplement: Supplementary file 5 [file DataSheet5.ZIP › Original unedited images (western blot)/Figure4/Figure4A and 4C-Tau.tif]

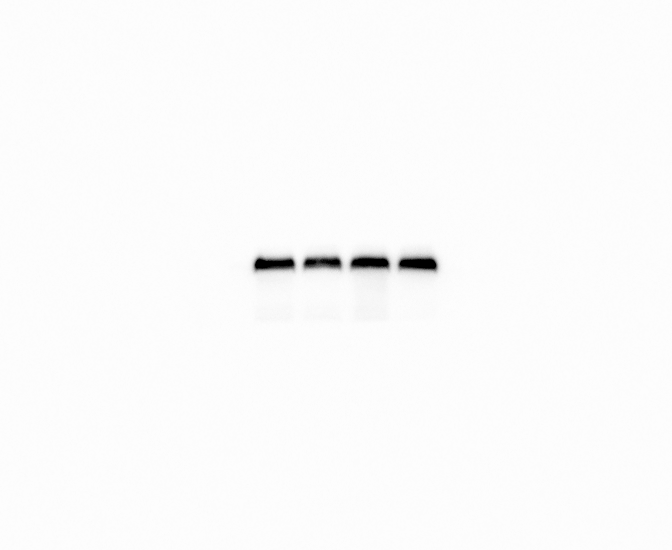

Supplement: Supplementary file 5 [file DataSheet5.ZIP › Original unedited images (western blot)/Figure4/Figure4E-Camk.tif]

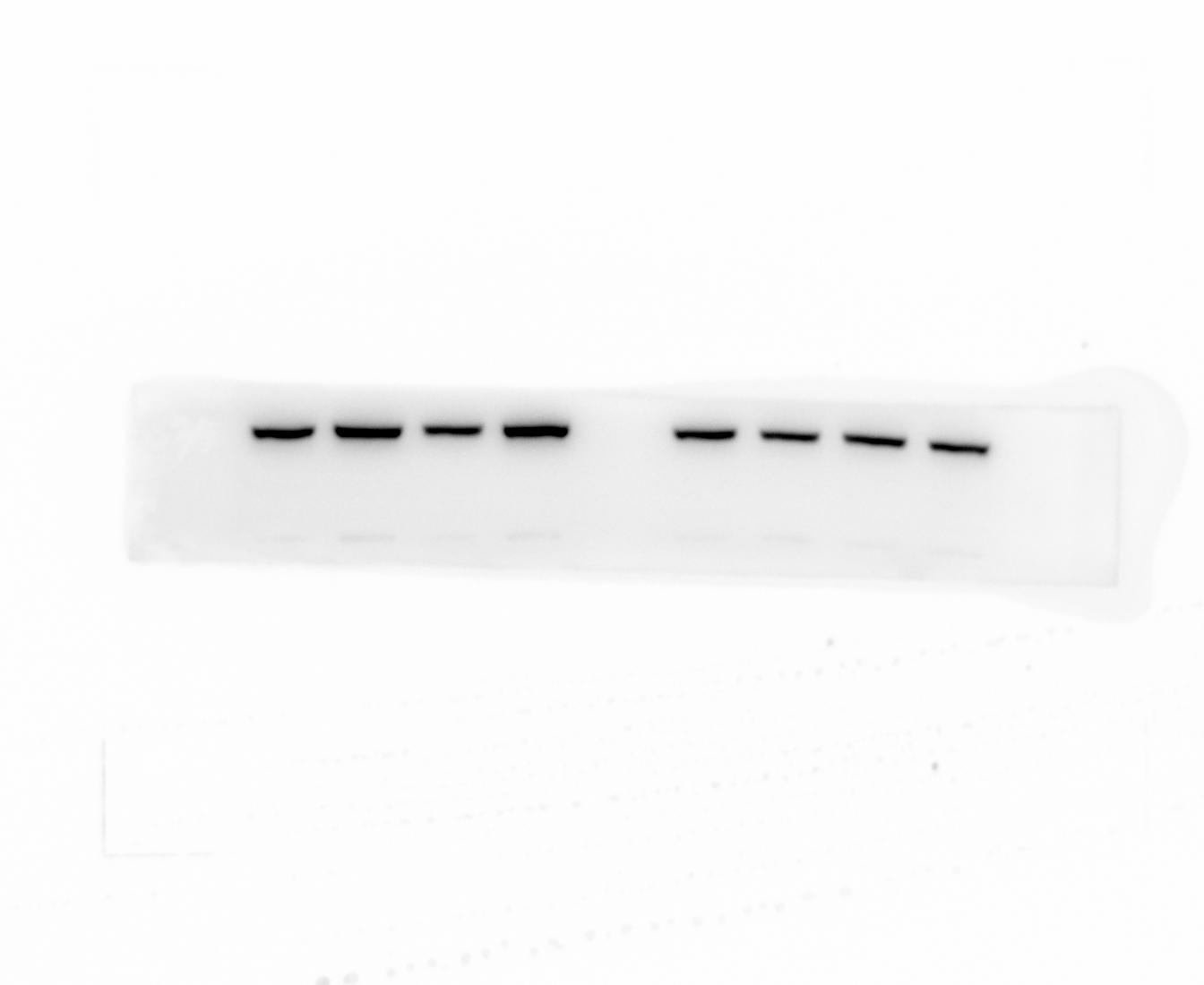

Supplement: Supplementary file 5 [file DataSheet5.ZIP › Original unedited images (western blot)/Figure4/Figure4E-D-PP2A.tif]

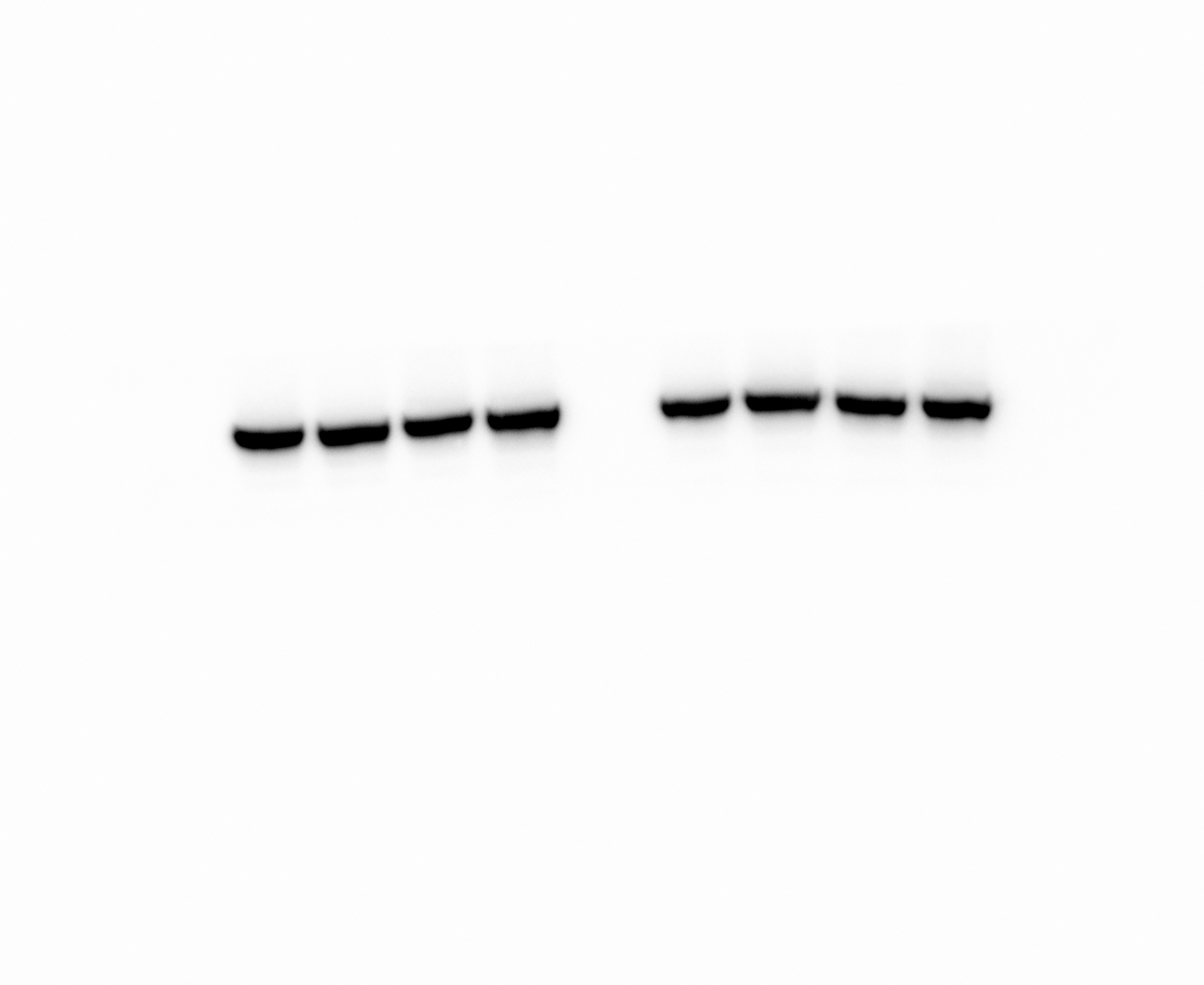

Supplement: Supplementary file 5 [file DataSheet5.ZIP › Original unedited images (western blot)/Figure4/Figure4E-GSK.tif]

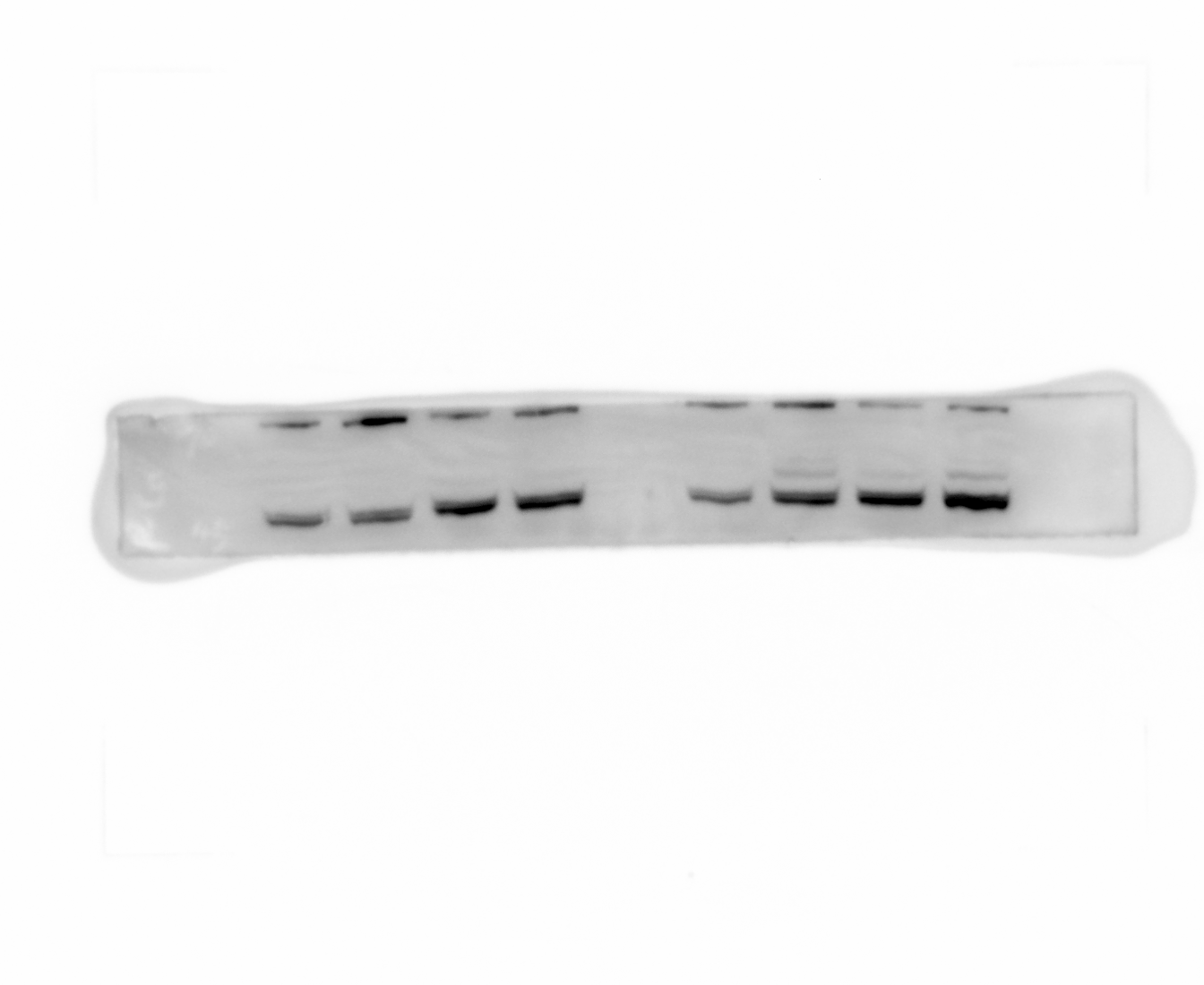

Supplement: Supplementary file 5 [file DataSheet5.ZIP › Original unedited images (western blot)/Figure4/Figure4E-P-camk.tif]

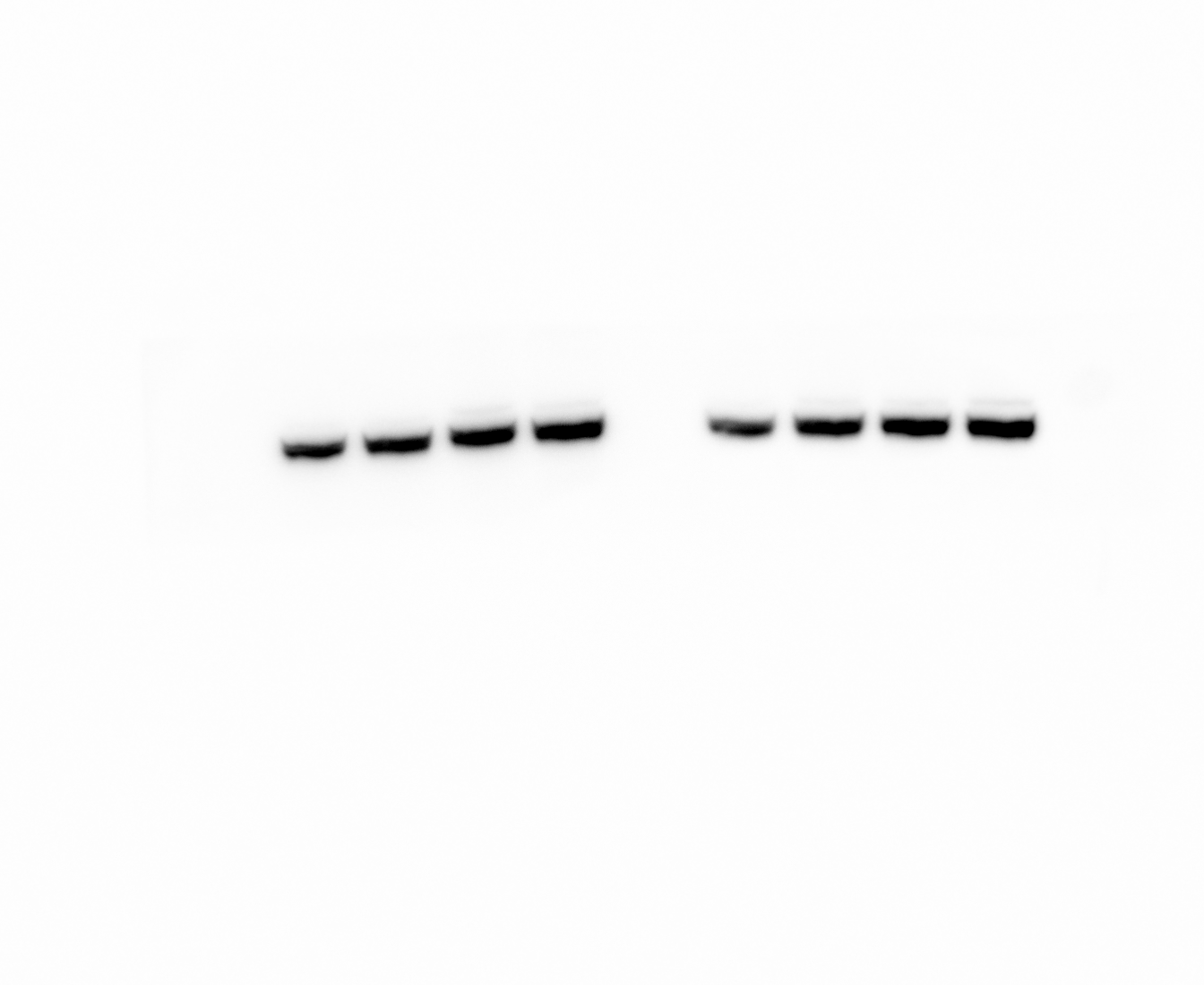

Supplement: Supplementary file 5 [file DataSheet5.ZIP › Original unedited images (western blot)/Figure4/Figure4E-P-GSK.tif]

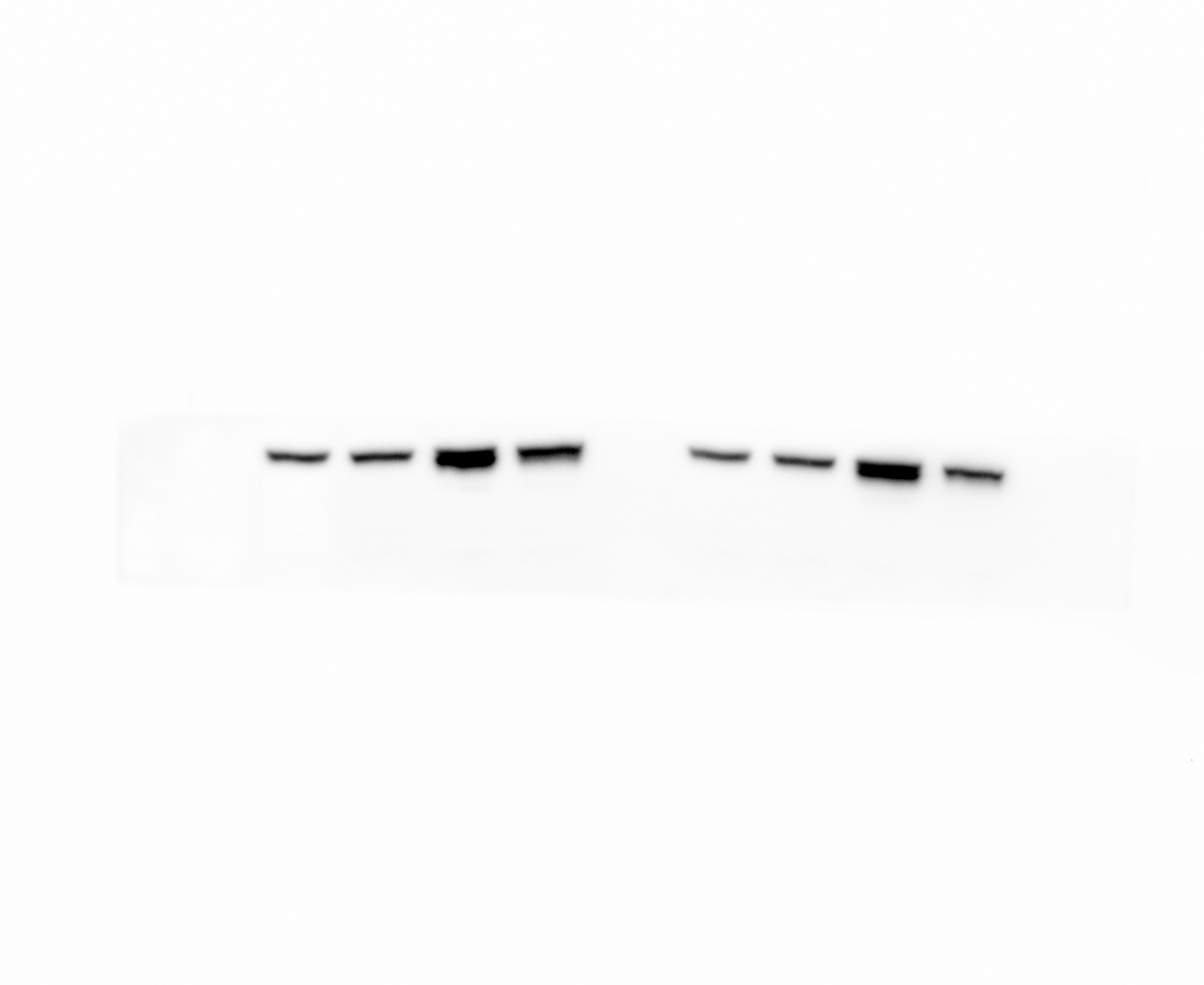

Supplement: Supplementary file 5 [file DataSheet5.ZIP › Original unedited images (western blot)/Figure4/Figure4E-pp2a.tif]

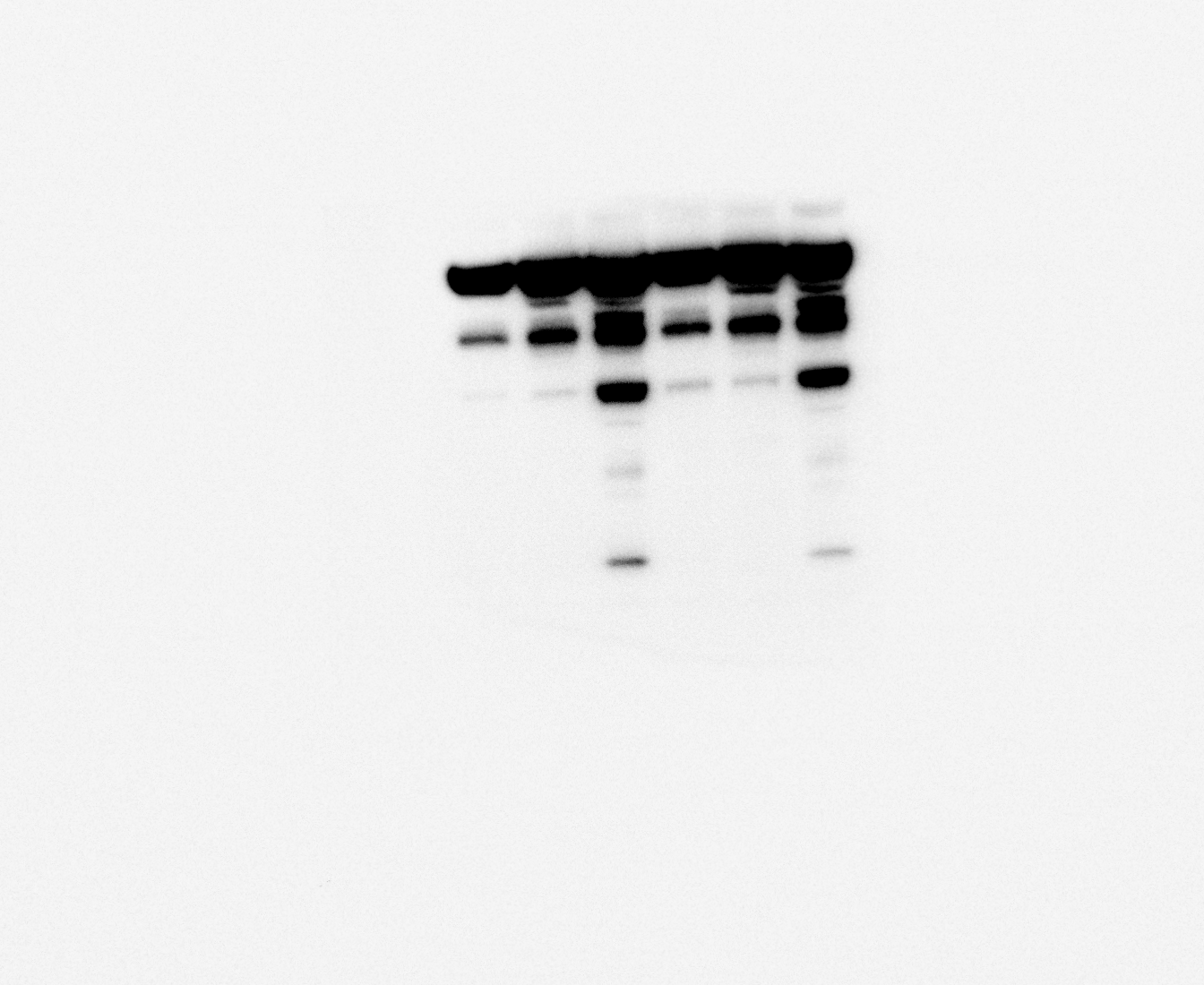

Supplement: Supplementary file 5 [file DataSheet5.ZIP › Original unedited images (western blot)/Figure5/Figure5A-Caspase1.tif]

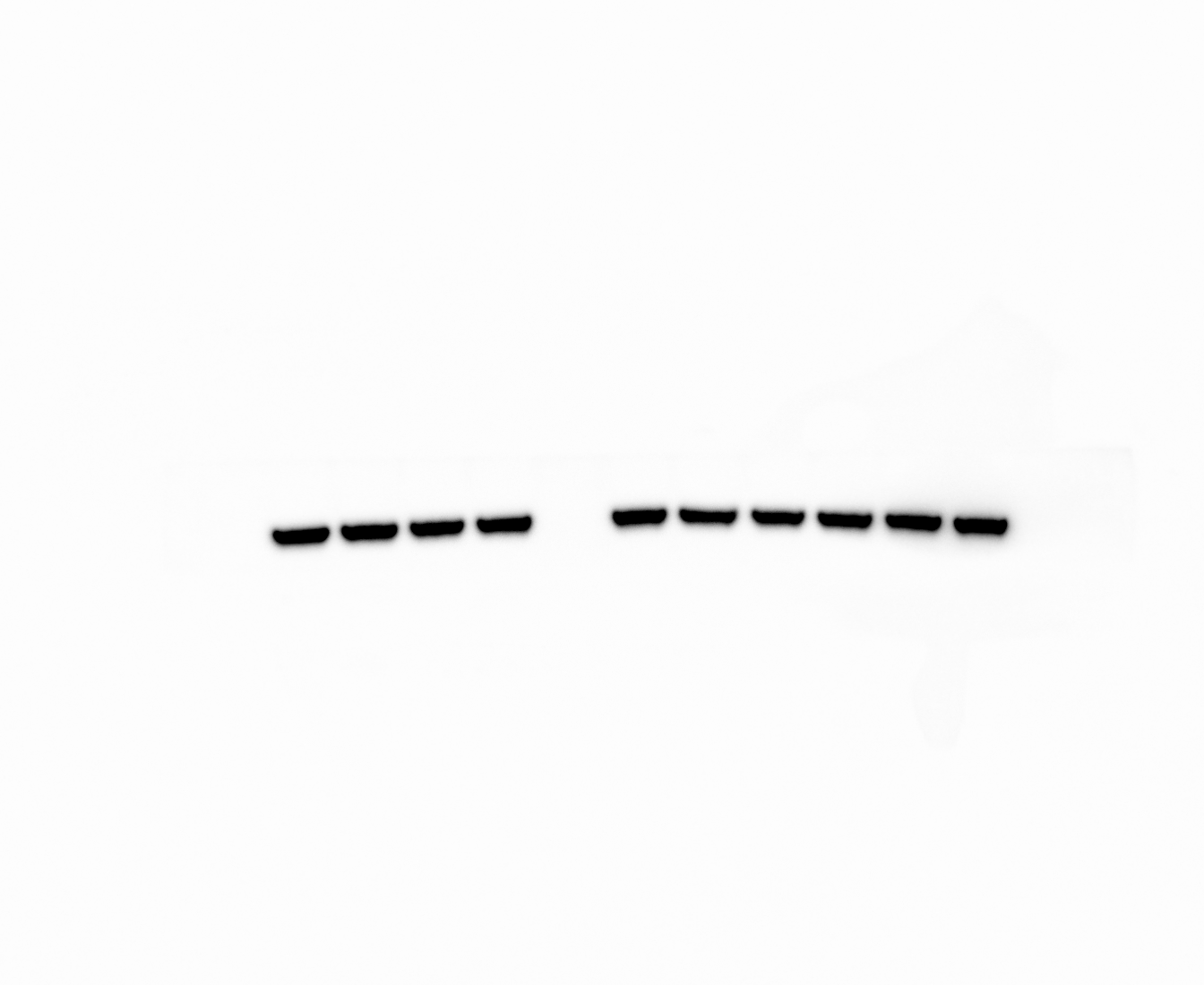

Supplement: Supplementary file 5 [file DataSheet5.ZIP › Original unedited images (western blot)/Figure5/Figure5A-GAPDH.tif]

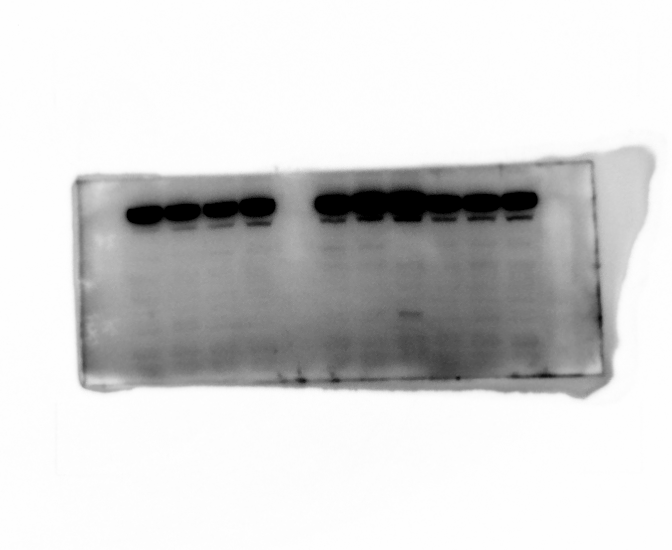

Supplement: Supplementary file 5 [file DataSheet5.ZIP › Original unedited images (western blot)/Figure5/Figure5A-GSDMD.tif]

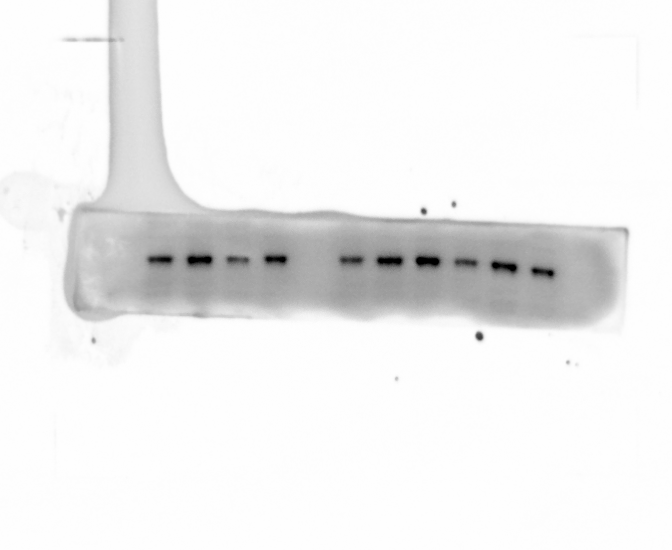

Supplement: Supplementary file 5 [file DataSheet5.ZIP › Original unedited images (western blot)/Figure5/Figure5A-IL-18.tif]

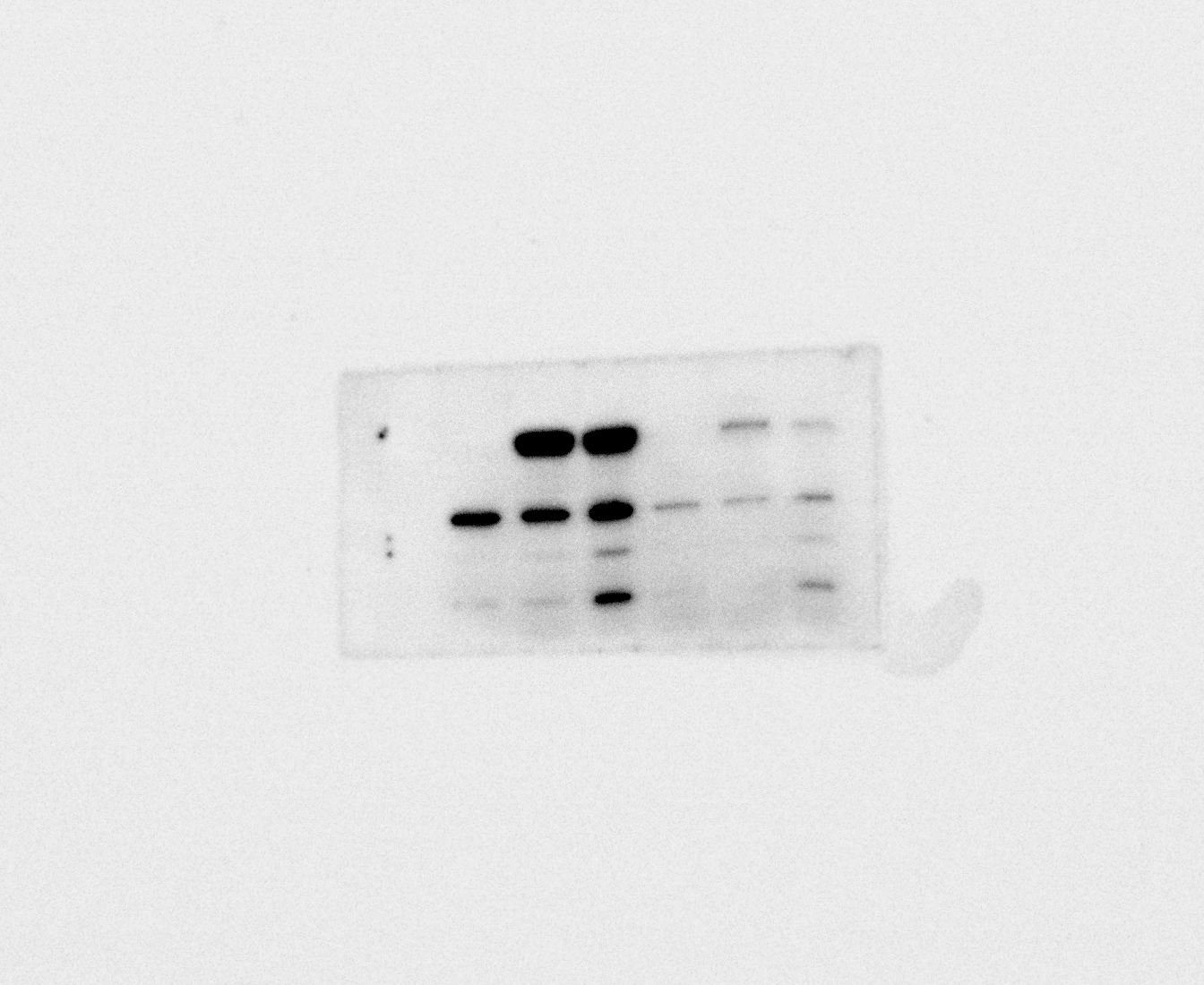

Supplement: Supplementary file 5 [file DataSheet5.ZIP › Original unedited images (western blot)/Figure5/Figure5A-IL-1β.tif]

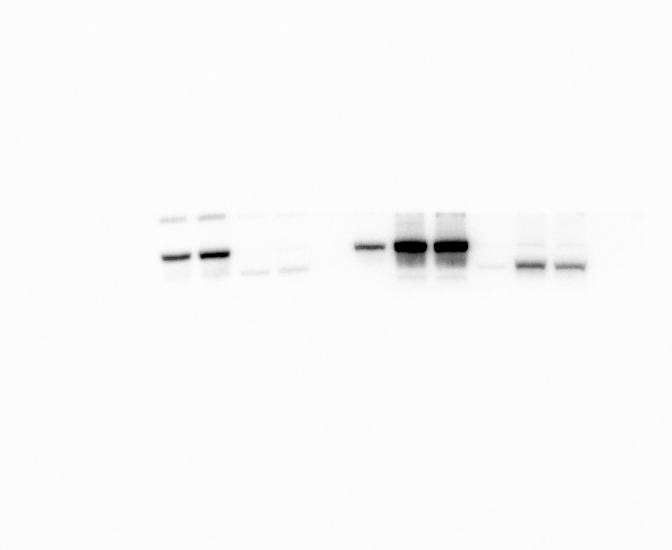

Supplement: Supplementary file 5 [file DataSheet5.ZIP › Original unedited images (western blot)/Figure5/Figure5A-NLRP3.tif]

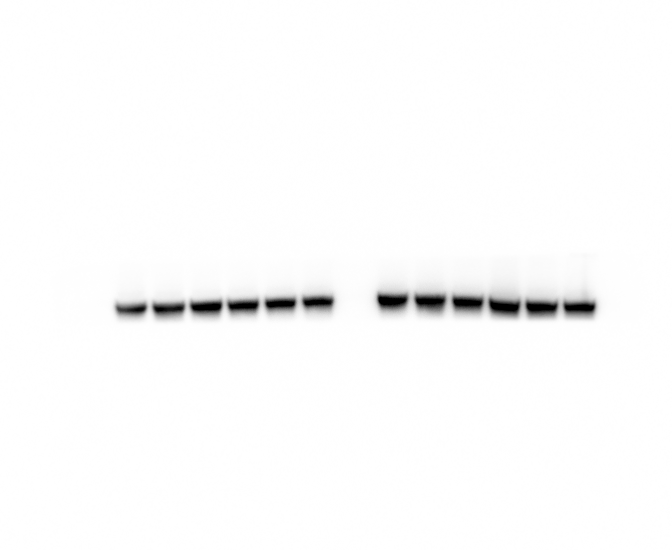

Supplement: Supplementary file 5 [file DataSheet5.ZIP › Original unedited images (western blot)/Figure5/Figure5B-Camk.tif]

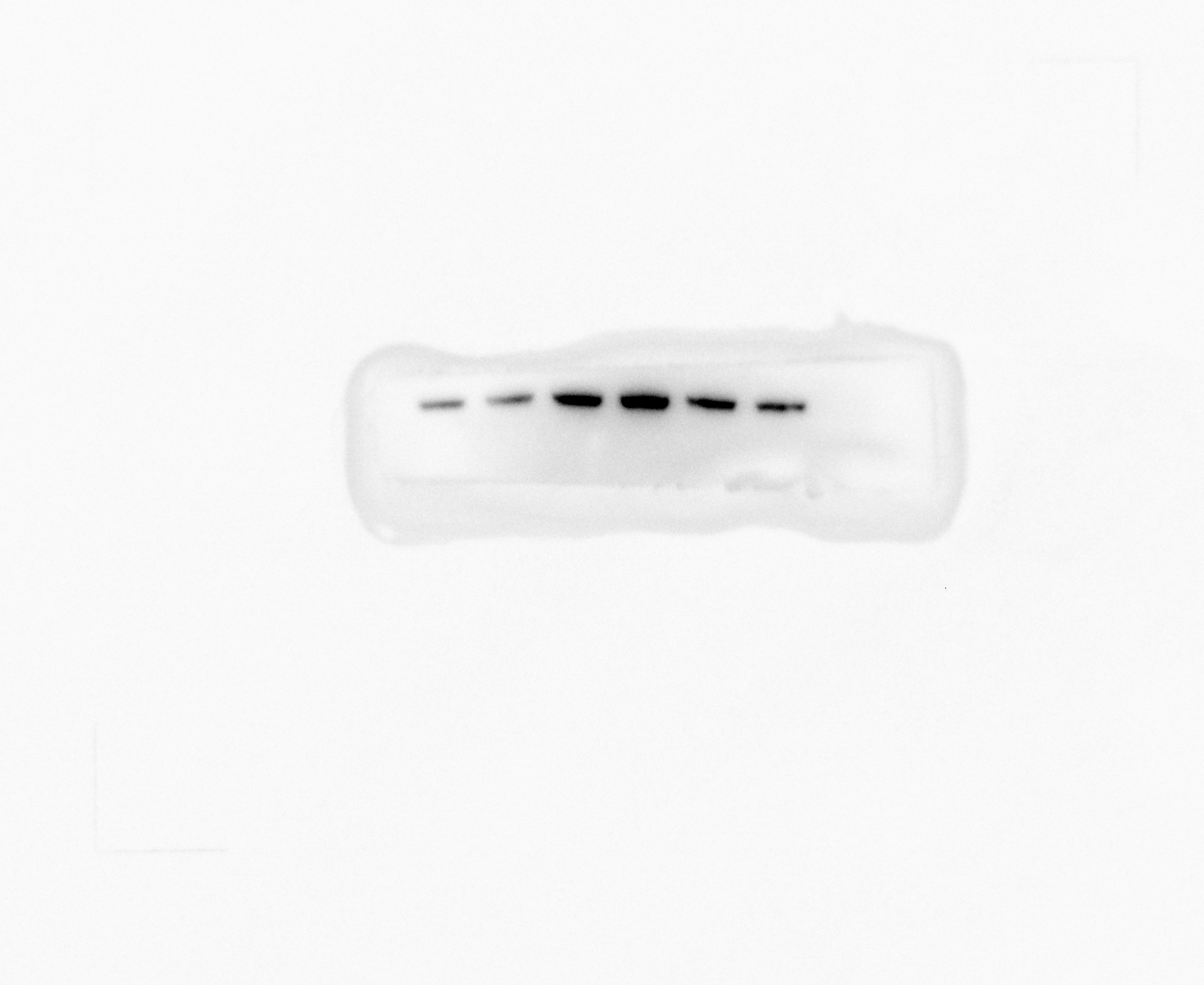

Supplement: Supplementary file 5 [file DataSheet5.ZIP › Original unedited images (western blot)/Figure5/Figure5B-D-PP2A.tif]

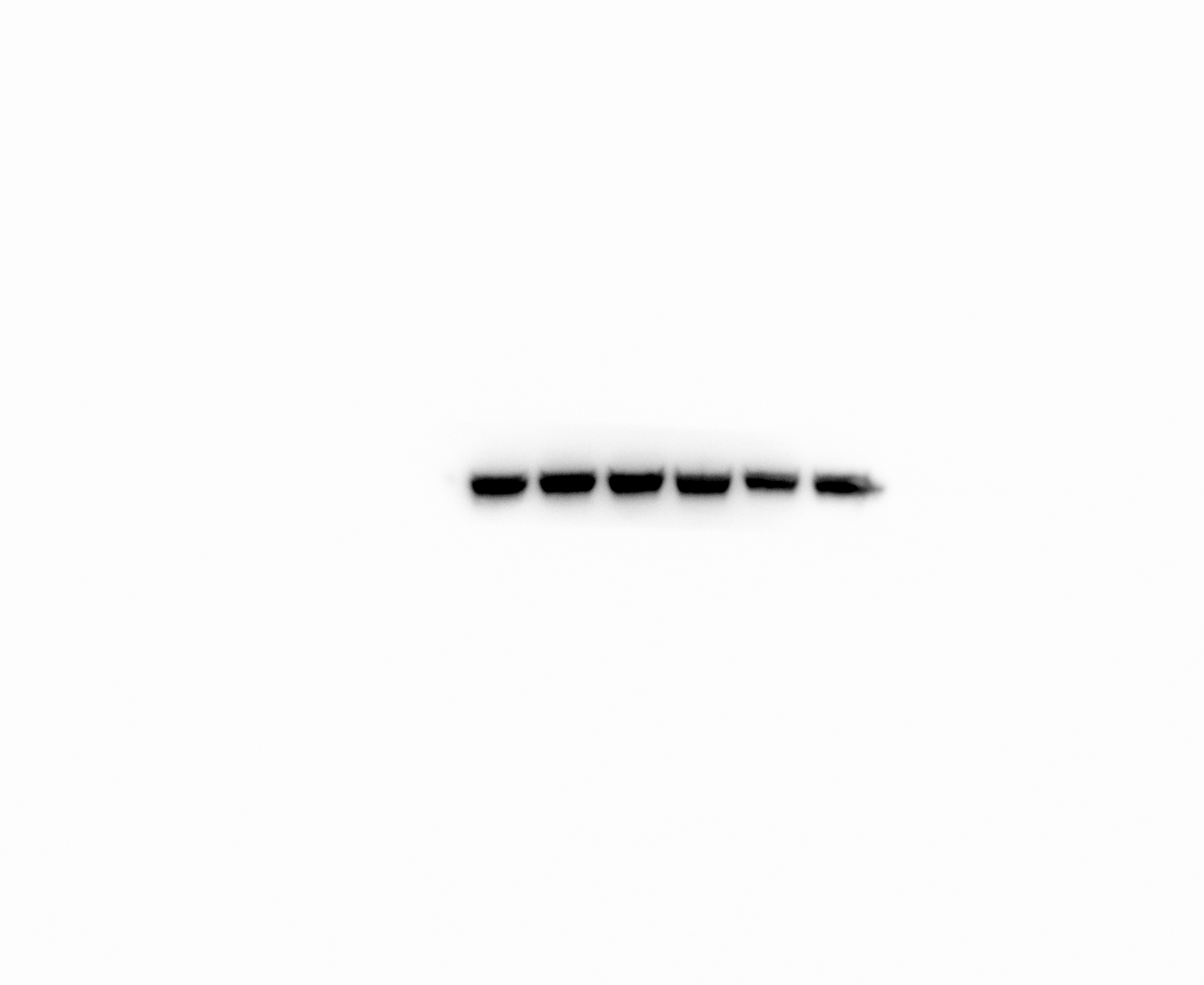

Supplement: Supplementary file 5 [file DataSheet5.ZIP › Original unedited images (western blot)/Figure5/Figure5B-GSK.tif]

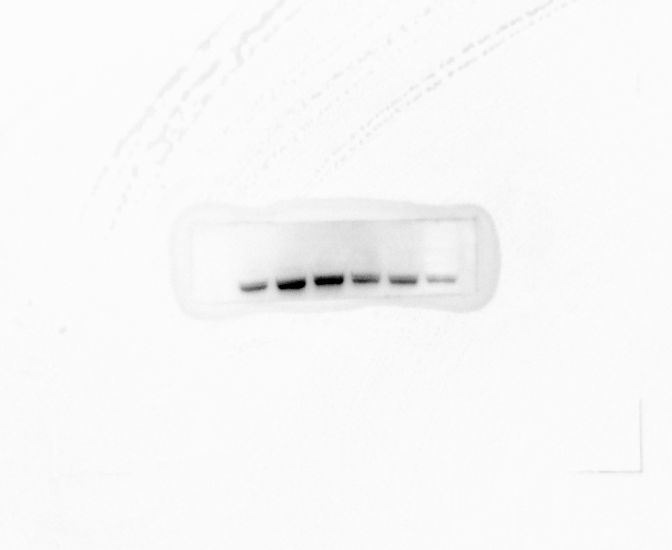

Supplement: Supplementary file 5 [file DataSheet5.ZIP › Original unedited images (western blot)/Figure5/Figure5B-P-CAMK.tif]

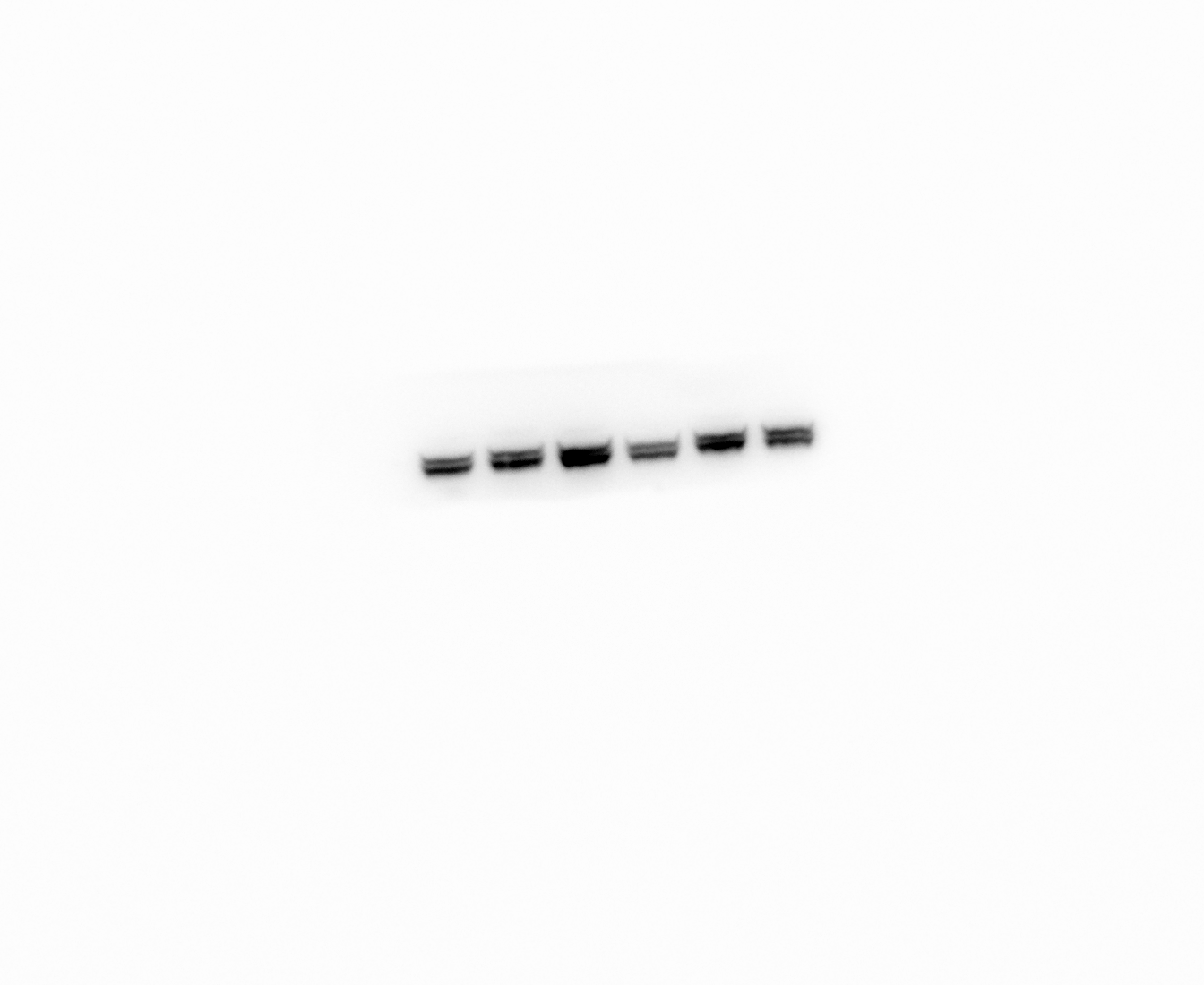

Supplement: Supplementary file 5 [file DataSheet5.ZIP › Original unedited images (western blot)/Figure5/Figure5B-P-GSK.tif]

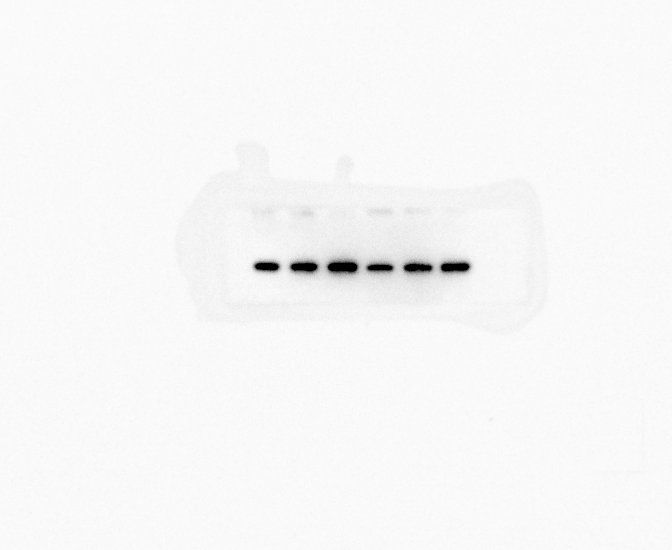

Supplement: Supplementary file 5 [file DataSheet5.ZIP › Original unedited images (western blot)/Figure5/Figure5B-P-Tau.tif]

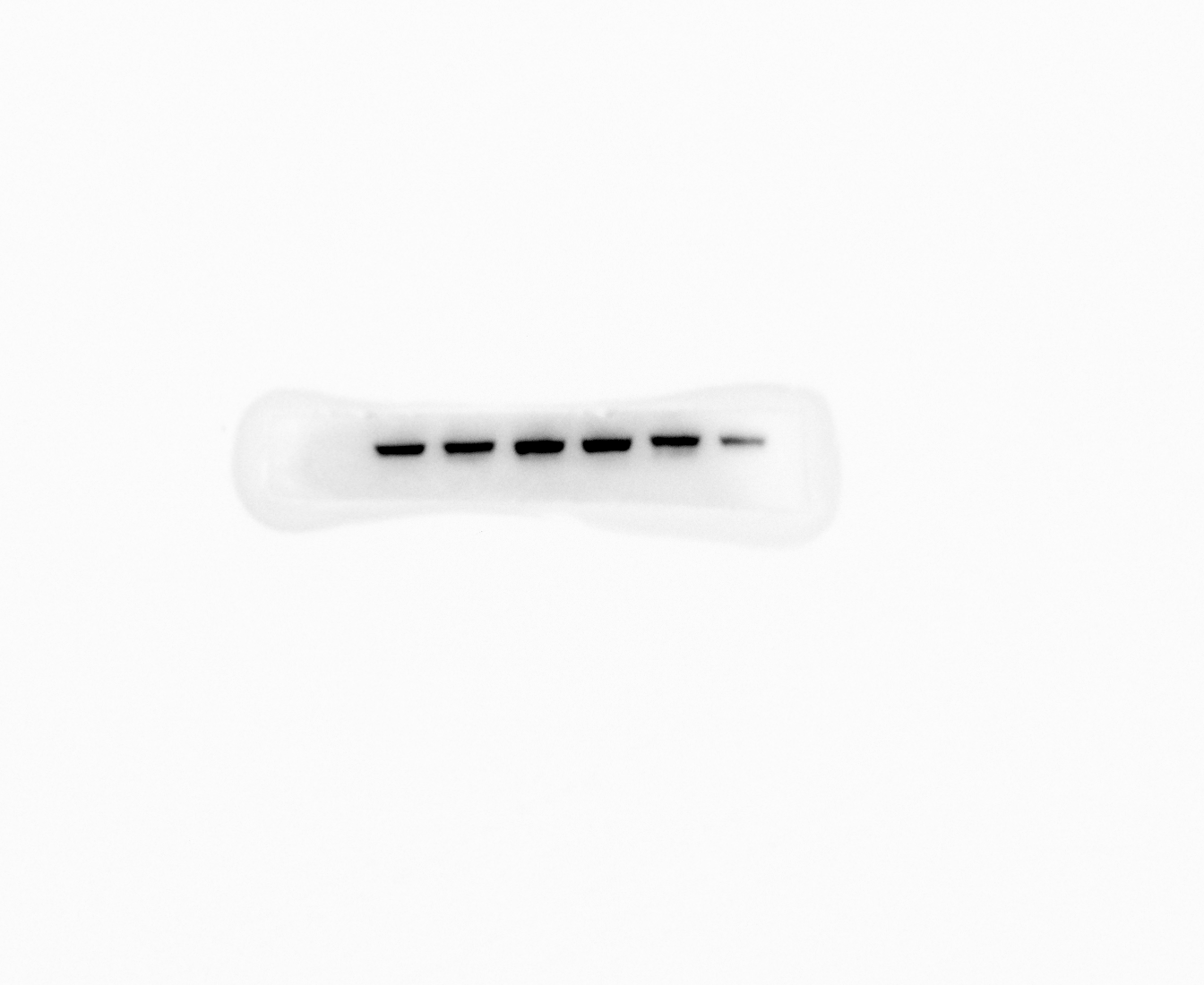

Supplement: Supplementary file 5 [file DataSheet5.ZIP › Original unedited images (western blot)/Figure5/Figure5B-PP2A.tif]

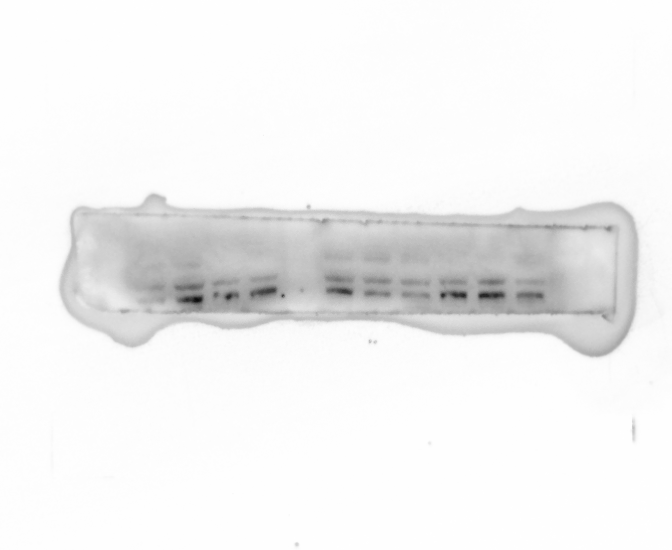

Supplement: Supplementary file 5 [file DataSheet5.ZIP › Original unedited images (western blot)/Figure5/Figure5B-Tau.tif]

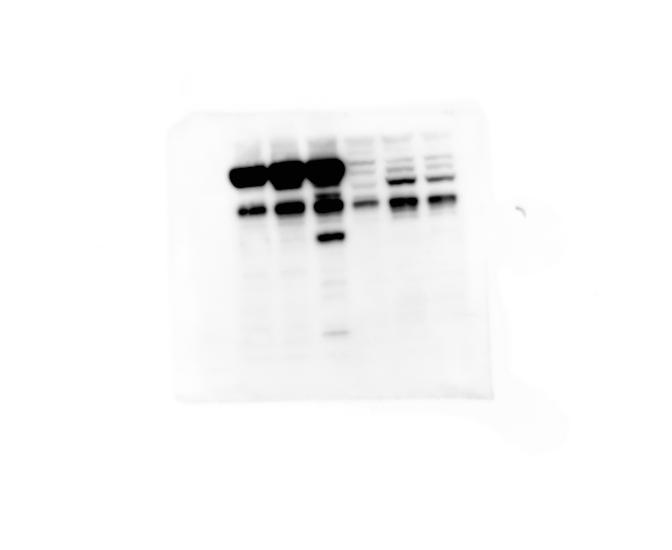

Supplement: Supplementary file 5 [file DataSheet5.ZIP › Original unedited images (western blot)/Figure6/Figure6A-caspase1.tif]

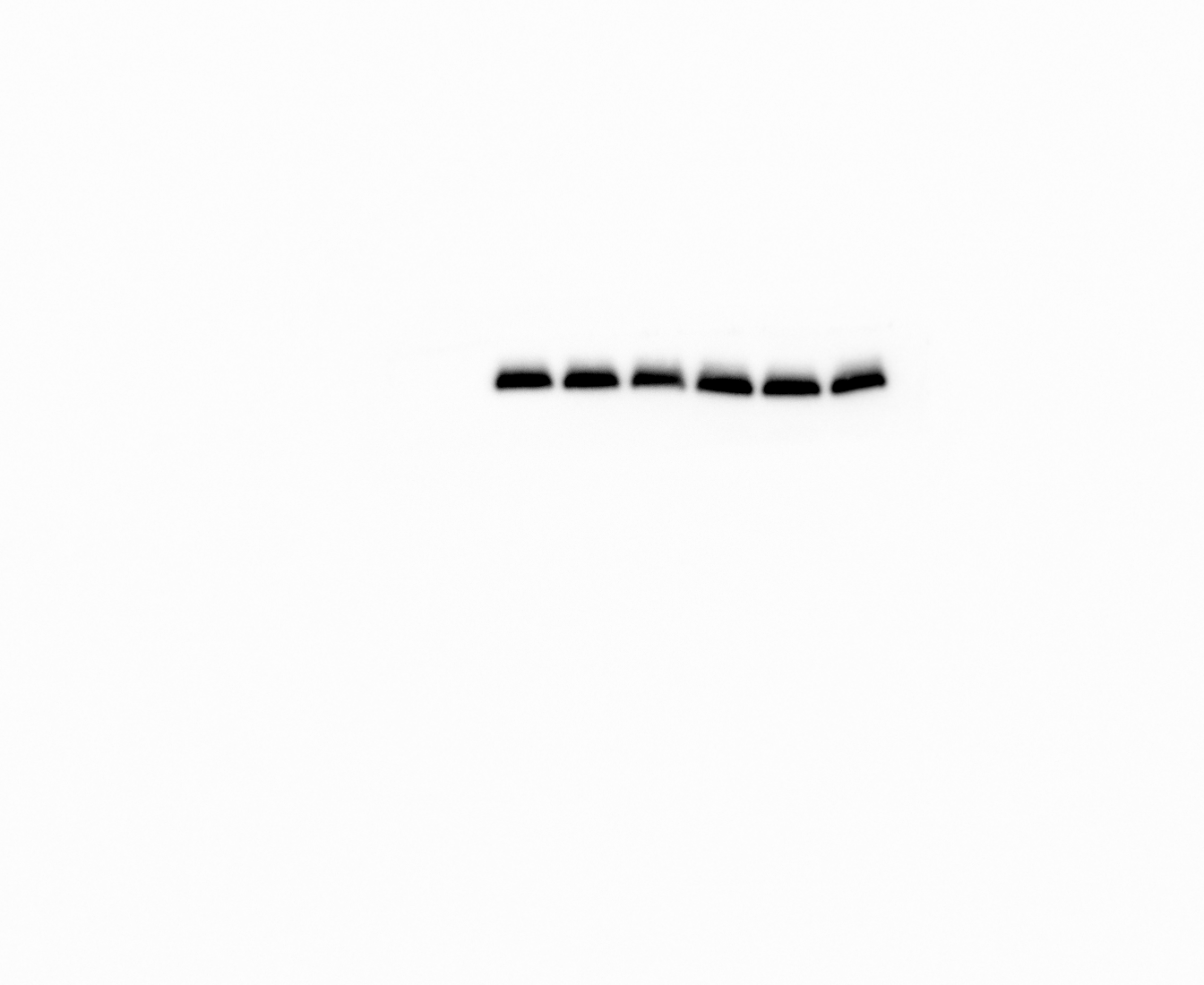

Supplement: Supplementary file 5 [file DataSheet5.ZIP › Original unedited images (western blot)/Figure6/Figure6A-GAPDH.tif]

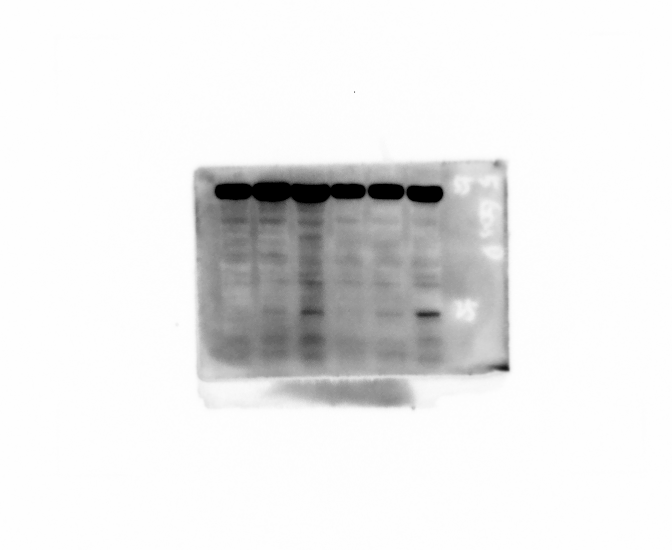

Supplement: Supplementary file 5 [file DataSheet5.ZIP › Original unedited images (western blot)/Figure6/Figure6A-Gasdermine D.tif]

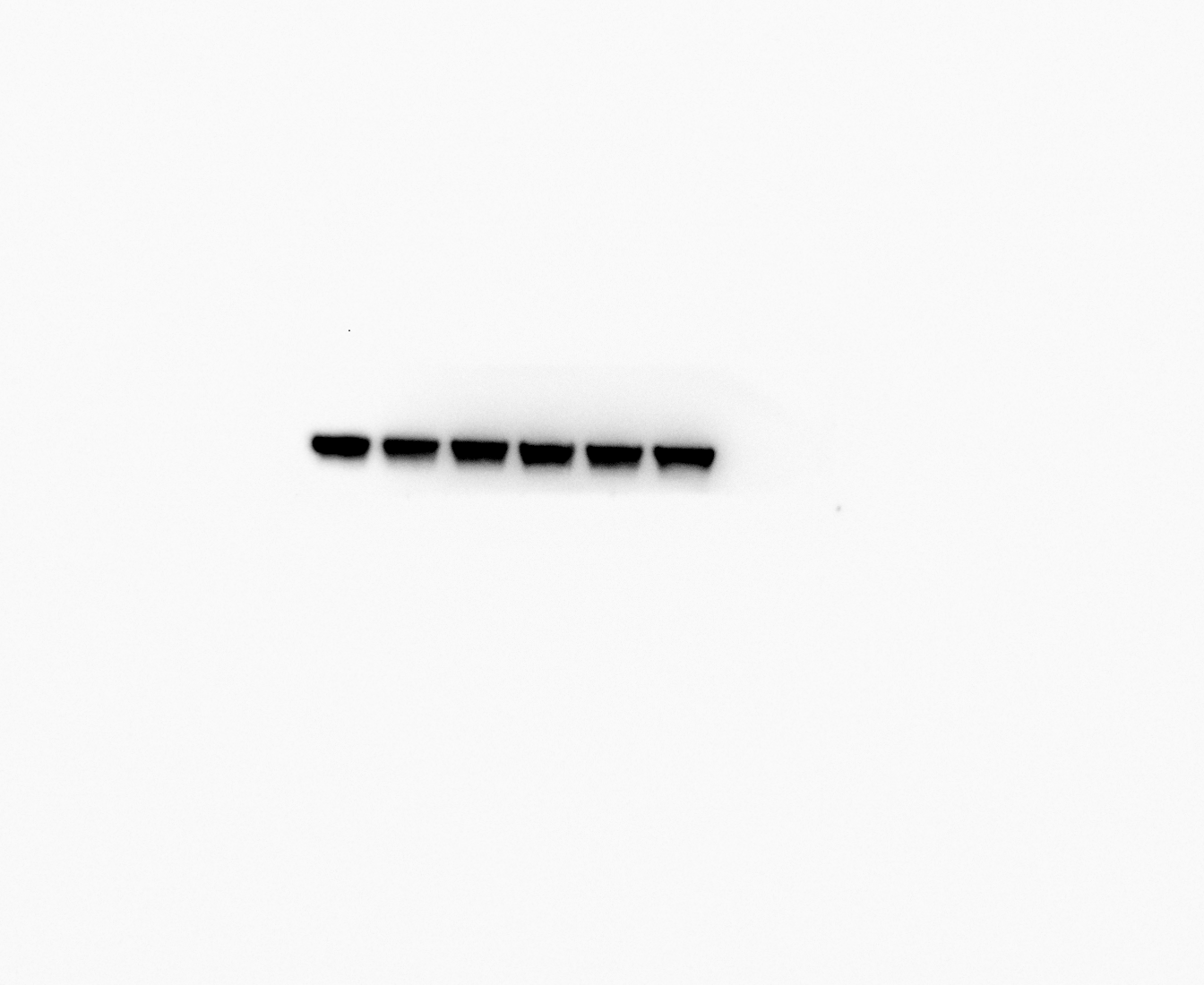

Supplement: Supplementary file 5 [file DataSheet5.ZIP › Original unedited images (western blot)/Figure6/Figure6B-GAPDH.tif]

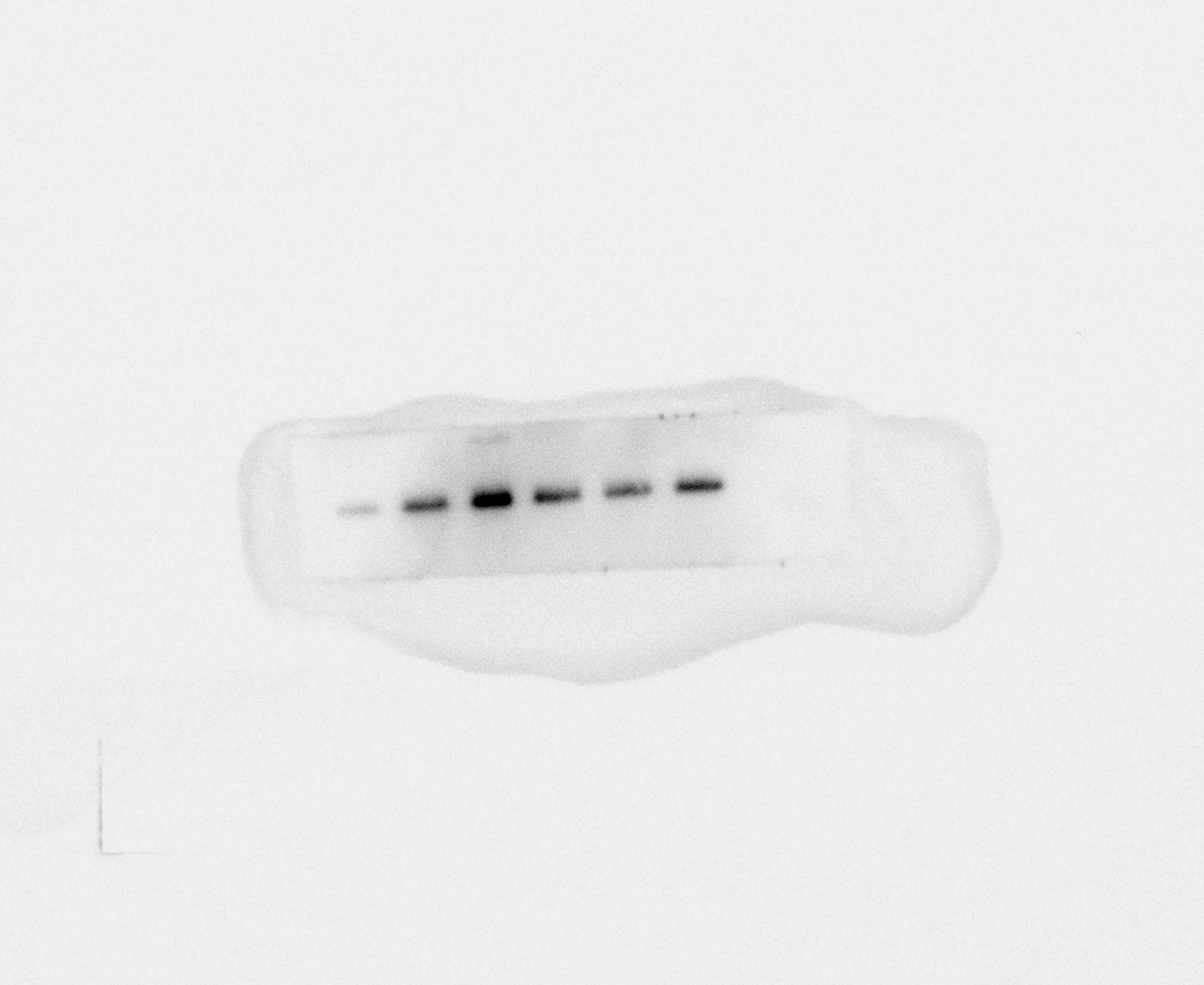

Supplement: Supplementary file 5 [file DataSheet5.ZIP › Original unedited images (western blot)/Figure6/Figure6B-IL-18.tif]

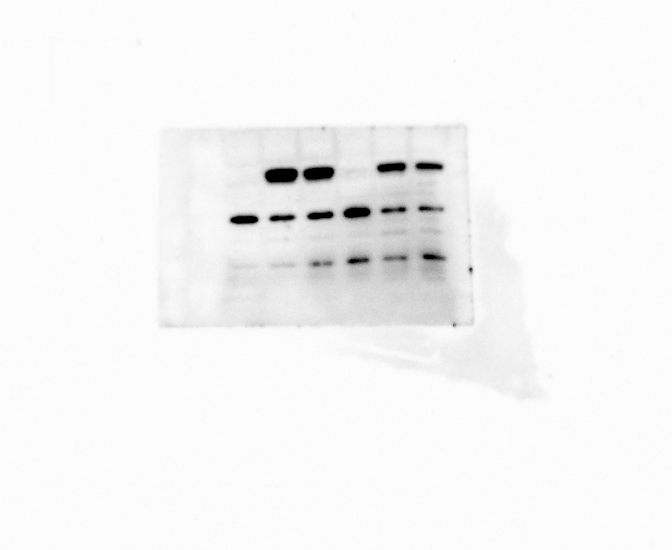

Supplement: Supplementary file 5 [file DataSheet5.ZIP › Original unedited images (western blot)/Figure6/Figure6B-IL-1β.tif]

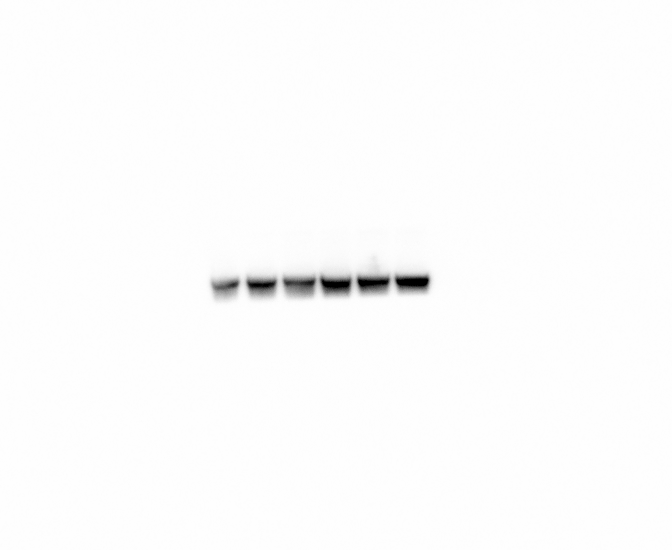

Supplement: Supplementary file 5 [file DataSheet5.ZIP › Original unedited images (western blot)/Figure6/Figure6C-camk.tif]

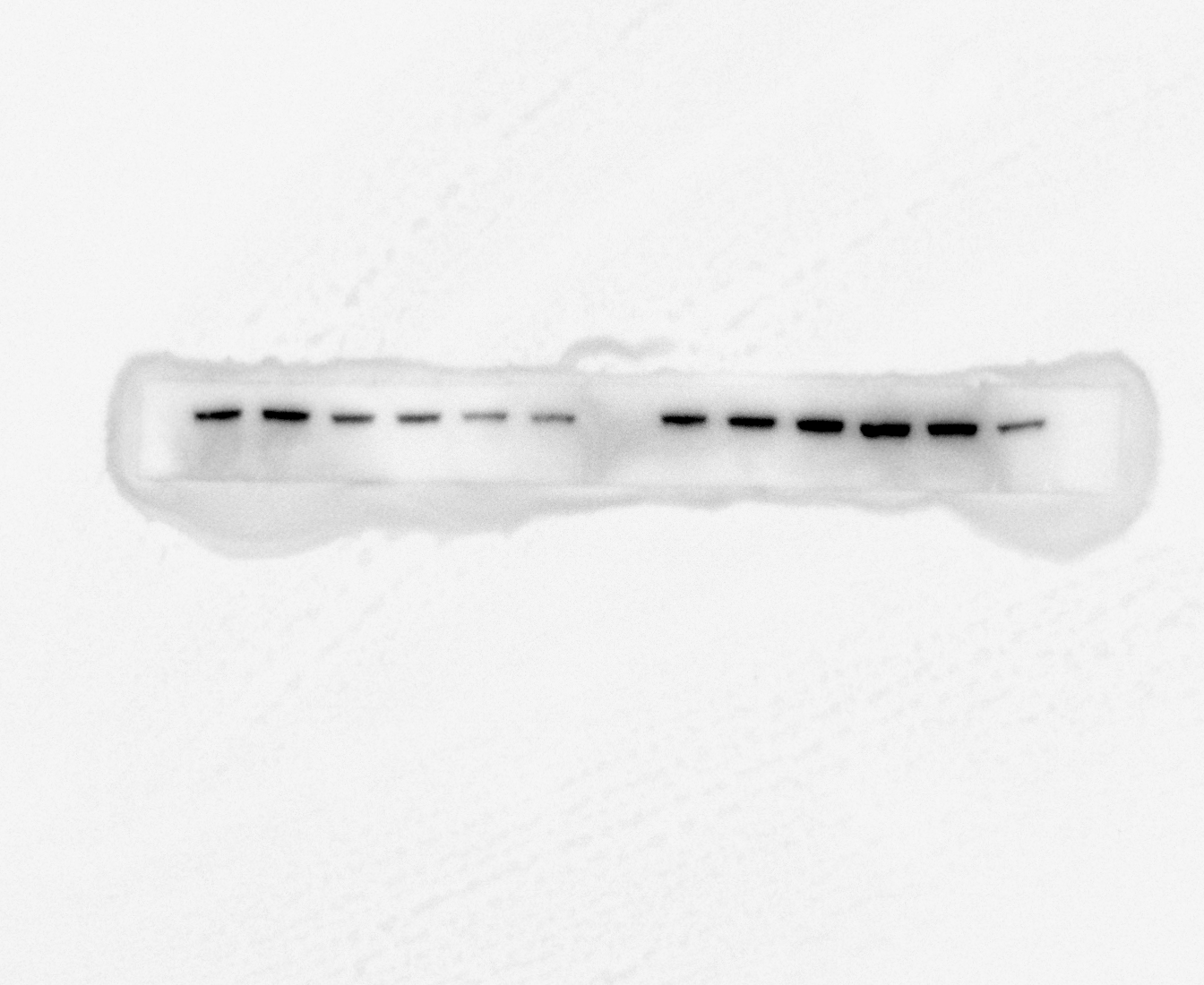

Supplement: Supplementary file 5 [file DataSheet5.ZIP › Original unedited images (western blot)/Figure6/Figure6C-D-pp2a.tif]

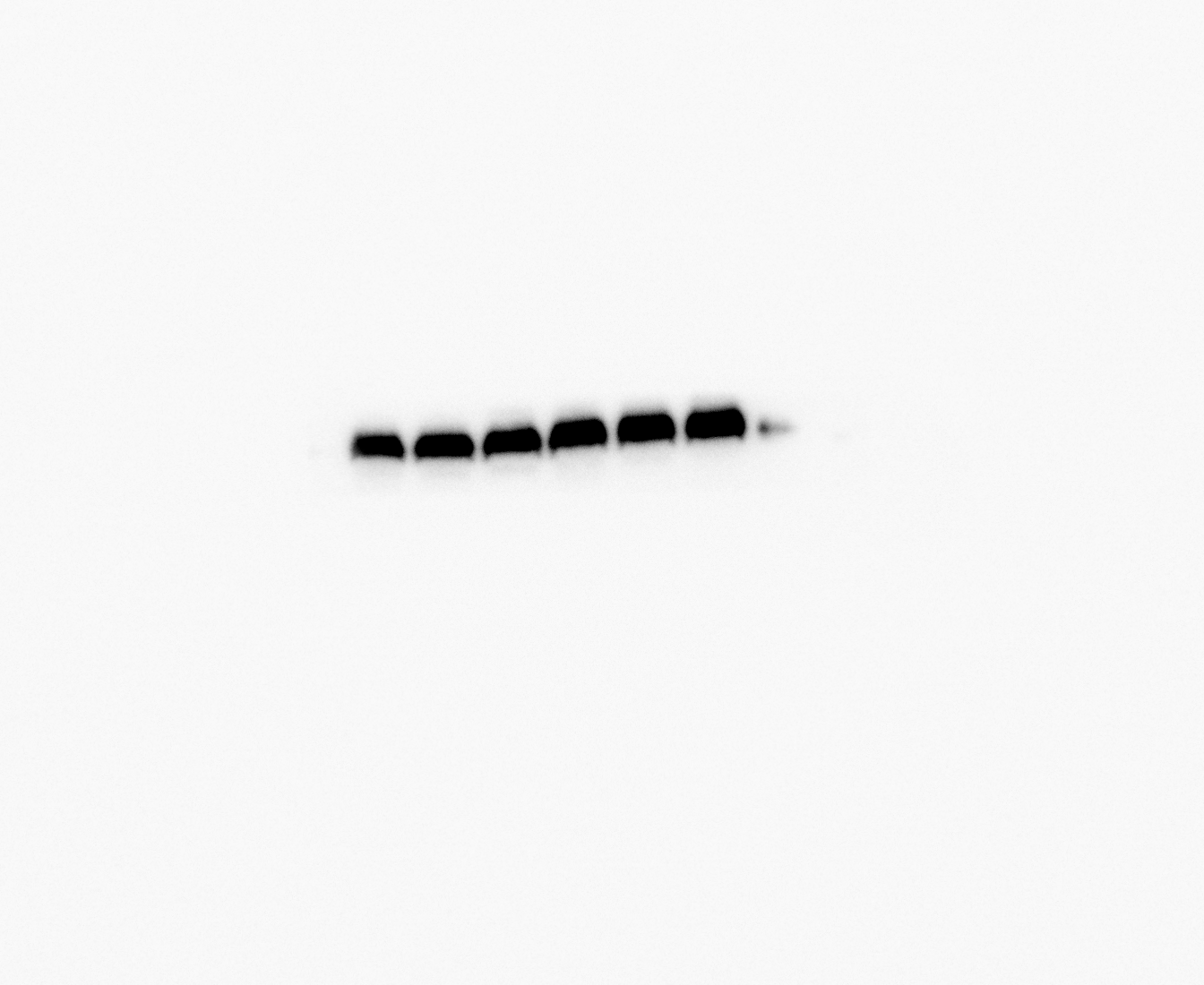

Supplement: Supplementary file 5 [file DataSheet5.ZIP › Original unedited images (western blot)/Figure6/Figure6C-GSK.tif]

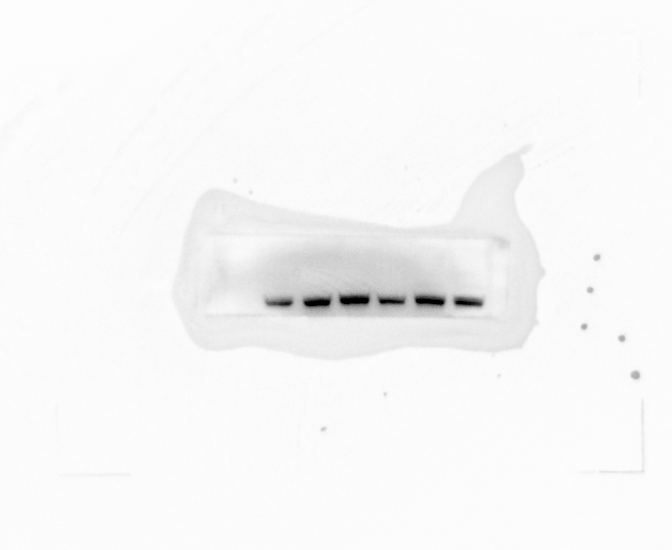

Supplement: Supplementary file 5 [file DataSheet5.ZIP › Original unedited images (western blot)/Figure6/Figure6C-p-camk.tif]

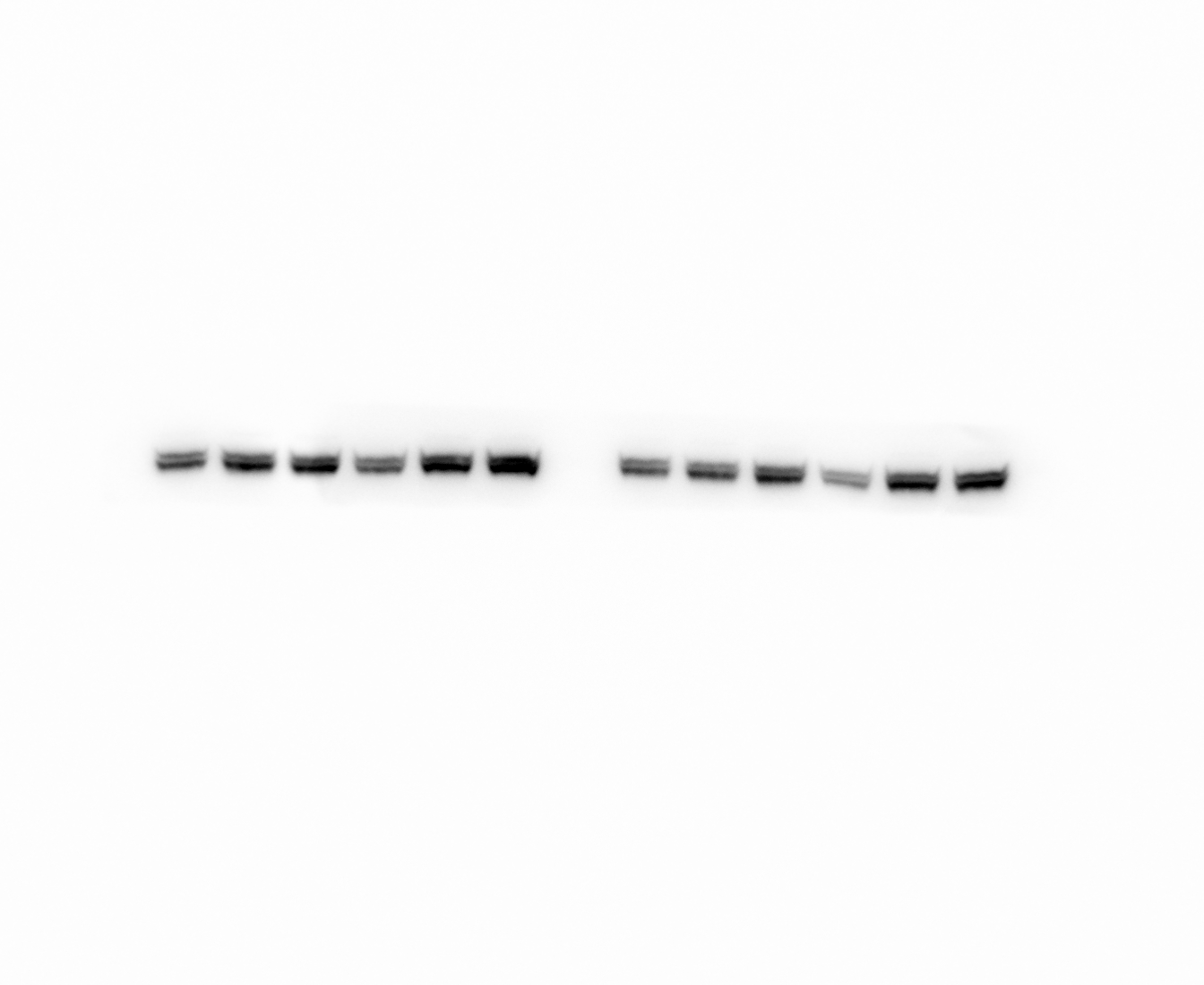

Supplement: Supplementary file 5 [file DataSheet5.ZIP › Original unedited images (western blot)/Figure6/Figure6C-P-GSK.tif]

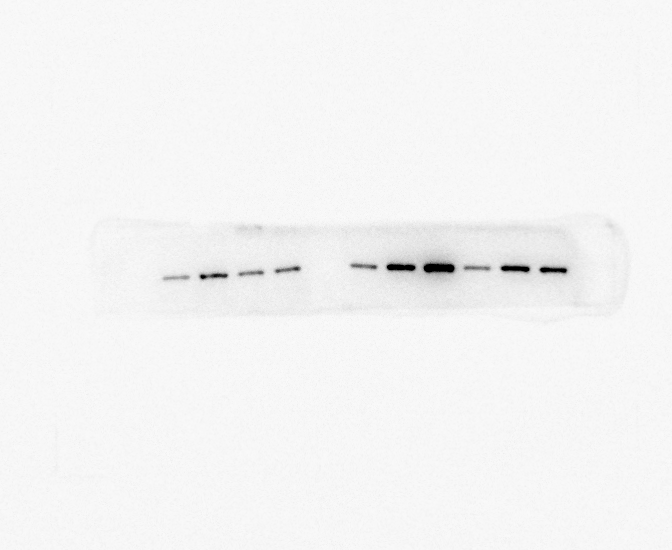

Supplement: Supplementary file 5 [file DataSheet5.ZIP › Original unedited images (western blot)/Figure6/Figure6C-p-tau.tif]

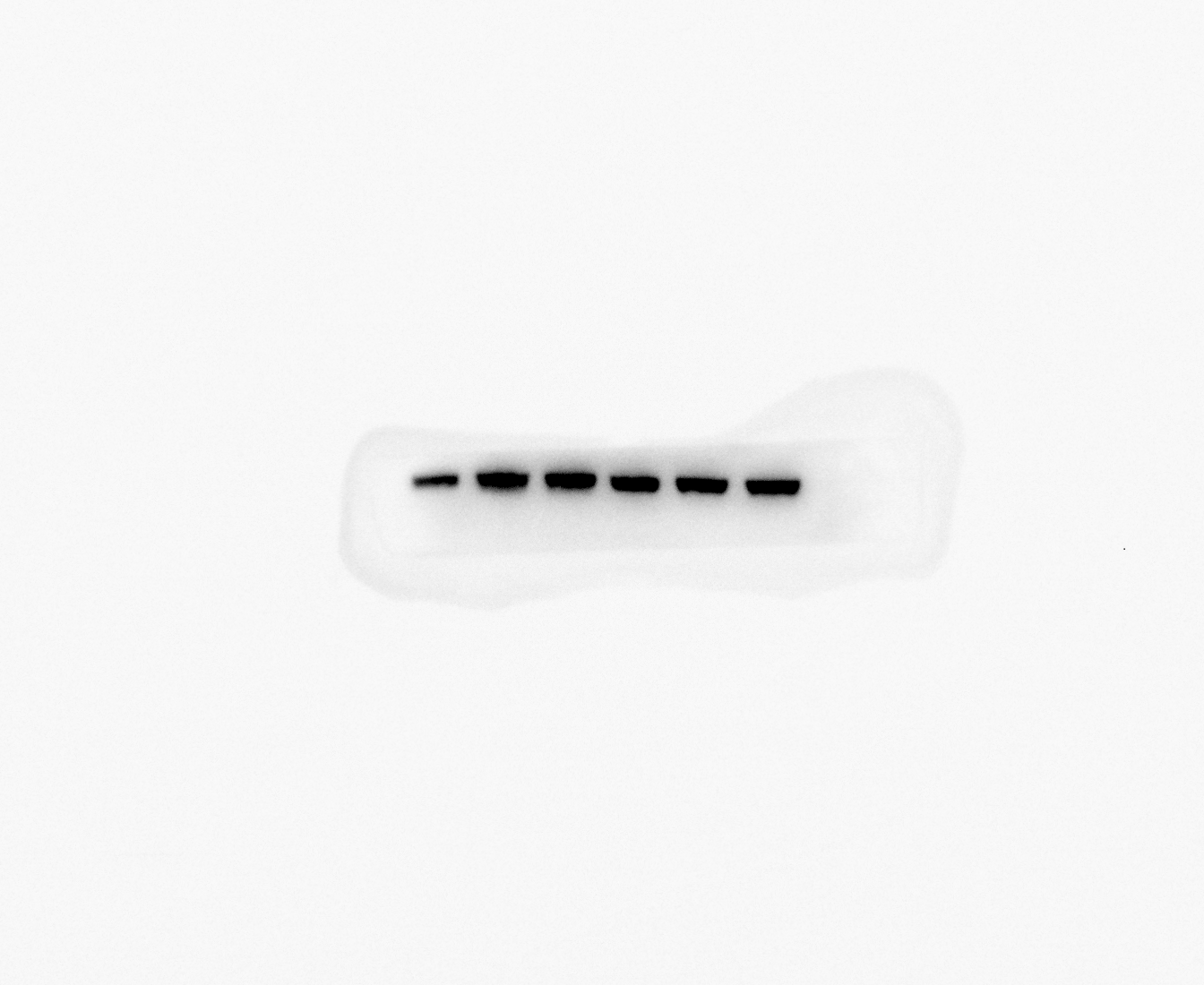

Supplement: Supplementary file 5 [file DataSheet5.ZIP › Original unedited images (western blot)/Figure6/Figure6C-pp2a.tif]

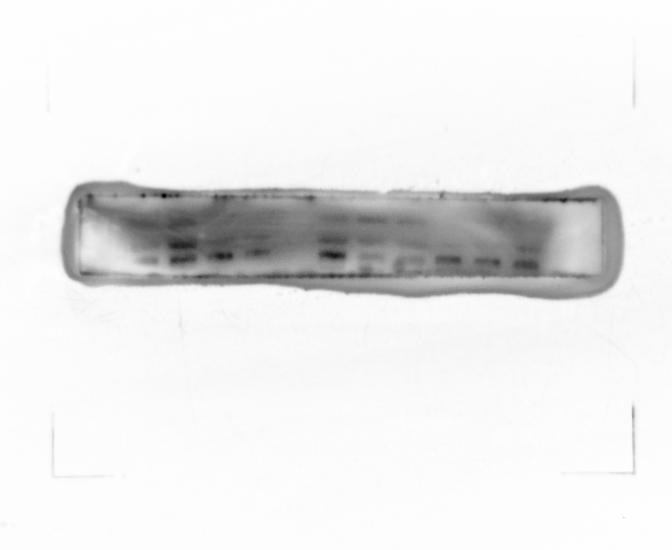

Supplement: Supplementary file 5 [file DataSheet5.ZIP › Original unedited images (western blot)/Figure6/Figure6C-tau.tif]
